# Magnetoelectrocatalysis: Evidence from the Hydrogen Evolution Reaction

**DOI:** 10.1021/acsphyschemau.3c00039

**Published:** 2024-01-03

**Authors:** Krysti L. Knoche Gupta, Heung Chan Lee, Johna Leddy

**Affiliations:** Department of Chemistry, University of Iowa, Iowa City, Iowa 52240, United States

**Keywords:** magnetoelectrocatalysis, magnetoelectrochemistry, magnetic gradients, HER, hydrogen evolution reaction, unpaired spins and kinetics, electron transfer kinetics

## Abstract

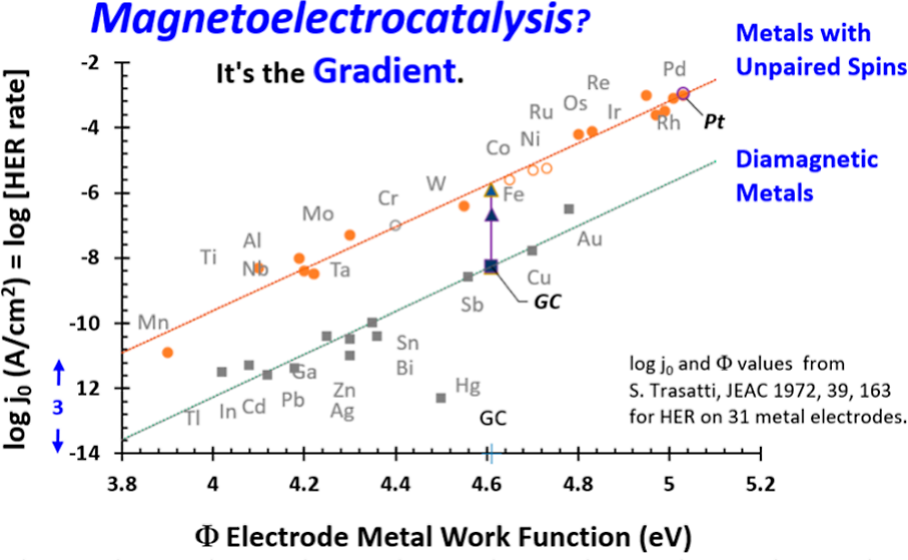

Hydrogen evolution reaction (HER) rates are higher where magnetic gradients are established at electrode surfaces. In comparison of literature data for metals with comparable work functions, we note 1000× higher rates for paramagnetic metals than diamagnetic metals. With unpaired electron spins, paramagnetic and ferromagnetic metals establish interfacial magnetic gradients. At diamagnetic electrodes, gradients are induced by addition of magnetized microparticles. Onset of hydrogen evolution for magnetized γ-Fe_2_O_3_ microparticles in Nafion on diamagnetic glassy carbon electrodes is lower by 190 mV (−18 kJ mol^–1^) relative to demagnetized microparticles. Chemically the same as demagnetized particles, the physical distinction of magnetic field and gradient at magnetized microparticles increases electron transfer rate. For magnetized Fe_3_O_4_ microparticles, the onset is lower by 280 mV (−27 kJ mol^–1^). Paramagnetic platinum electrodes are unaffected by addition of magnetized microparticles. Magnetoelectrocatalysis is established by magnetic gradients.

Electron transfer reactions ferry energy. Electrocatalysts redirect energy to increase electron transfer rates. Electron transfer rates at electrodes are conceptualized and measured in terms of potential, charge, and current. Potential measures energy; current measures rate; and potential gradients set electric fields. In addition to mass and charge, electrons have spin that sets magnetic properties. Given long established electromagnetic theory that entwines current with electric and magnetic fields and gradients, magnetic effects on electron transfer are perhaps anticipated, but not established.

Here, the magnetic gradient is critical to enhance electron transfer rates. Magnetic effects on electron transfer trigger new fundamental research in kinetics and electron transfer theory and inform electrocatalyst design from the novel perspective of magnetoelectrocatalysis.

Coupled questions that arise. Are there inherent magnetic effects on electron transfer? And, if so, can magnetic effects be exploited to facilitate electron transfer for better electrocatalysis? The answer to both questions is yes. Here, we first identify a substantial magnetic effect on the electrocatalysis of hydrogen evolution (Figure 1). In experimental studies, we then exploit the magnetoelectrocatalytic effect to markedly increase H_2_ evolution rates on diamagnetic glassy carbon cathodes. Magnetized microparticles deployed on electrodes induce magnetic gradients that increase the rate, as charted by arrows in Figure 1. The observation of inherent magnetic effects on HER electrocatalysis and its experimental exploitation, mutually substantiate magnetic impacts on electron transfer and provide rudimentary perspective on magnetoelectrocatalysis.

The hydrogen evolution reaction (HER) is an interfacial electron transfer reaction with the rate dependent on the electrode metal.
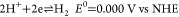
1

Reported as the exchange current density *j*_0_ (A/cm^2^), the interfacial electron transfer rate is measured from the electrode current and potential. In 1972, Trasatti critically culled the literature to determine best measured values of *j*_0_ at pH 0 for 31 metal electrodes (Table SI.1).^1^ Trasatti demonstrated log *j*_0_ varies linearly with work function Φ, the energy to remove an electron from the metal surface. In Figure 1, data fall into two parallel lines. For a given Φ, HER rates *j*_0_ on the upper line are 1000 times higher than *j*_0_ on the lower line.

Here, we note segregation of the high and low log *j*_0_ lines depends starkly on electrode magnetic properties. In Figure 1, the absolute and binary segregation of rates on the 31 electrodes are set by the unpaired electron spins inherent to the metal. All metals on the lower rate line are diamagnetic with no unpaired electrons; metals on the higher rate line are either paramagnetic or ferromagnetic with unpaired electrons. Unpaired electron spins set magnetic properties. The stark, binary discrimination of HER rates by the electron spin of the metal identifies a substantial magnetic effect on electron transfer and electrocatalysis.

A magnetic effect on HER electrocatalysis is further vetted experimentally. Magnetized microparticles deployed on diamagnetic electrodes impose steep magnetic gradients at the electrode electrolyte interface. Electrodes are modified with either films of the diamagnetic cation exchange polymer Nafion or composites of Nafion and siloxane-coated iron oxide microparticles. Magnetized composites on diamagnetic glassy carbon, gold, and mercury electrodes and n-GaAs and p-Si photocathodes establish substantially higher *j*_0_ as compared to Nafion films. In all cases, magnetized composites on diamagnetic electrodes amplify electrocatalytic rates, typically in the range of 10–1000 times.^4,5^

Notably, composites formed with demagnetized microparticles evolve H_2_ at rates comparable to or slightly lower than Nafion films. Chemical composition of magnetized and demagnetized composites is the same, but the physical distinction of magnetic gradients drives substantially higher HER rates on diamagnetic electrodes. In this initial report, we focus on magnetic effects at diamagnetic glassy carbon (GC) and paramagnetic platinum electrodes.

**Figure 1:**
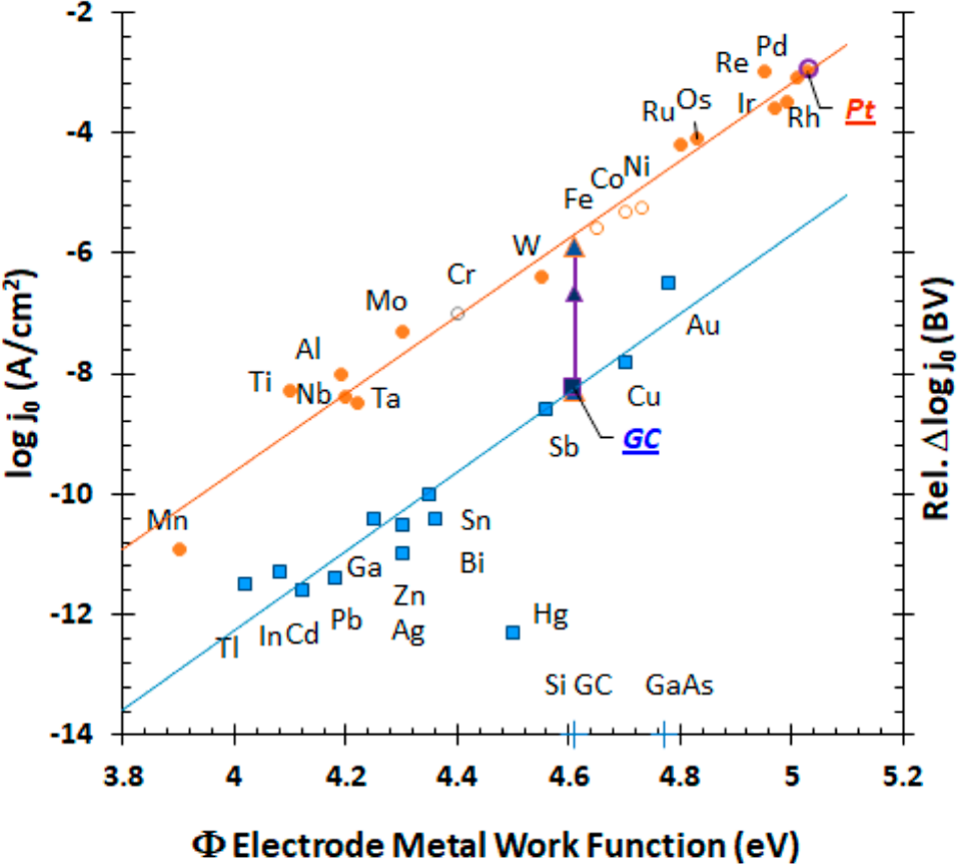
Log *j*_0_ versus Φ for HER. Exchange current density *j*_0_ is the rate constant. Work function Φ is the energy to remove an electron from the surface of a material to infinity in vacuum. Trasatti compiled *j*_0_ and Φ for 31 metal electrodes at pH 0 to yield linear Eyring plots of log *j*_0_ versus Φ that fall in two parallel lines.^1–3^ Here, we note electrodes with inherent electron spin [paramagnetic (filled circles), ferromagnetic (Fe, Ni, Co, open circles), and antiferromagnetic (Cr, gray open circle) metals] fall on the upper high rate line (log *j*_0_^spin^) and diamagnetic electrodes without inherent electron spin (squares) fall on the lower line (log *j*_0_^diam^). The binary discrimination of rates based on inherent electron spin of metal electrodes is without exception. For a given Φ, HER rates *j*_0_ are 1000 times higher at electrodes with spin than without spin. Magnetic properties of electrodes impact electrocatalysis. Magnetic microparticles introduce magnetic gradients to electrode surfaces that increase *j*_0_ from 10 to 1000 times. Magnetized microparticles on diamagnetic glassy carbon (GC) electrodes increase HER rates, shown as arrows for siloxane-shrouded, magnetized 1 μm γ-Fe_2_O_3_ (C1) microparticles (blue arrow) and 5 μm siloxane coated, magnetized Fe_3_O_4_ microparticles (gray arrow). Microparticles are held to electrodes in composites with Nafion. Increases in log *j*_0_ are relative to diamagnetic Nafion films. Magnetized Fe_3_O_4_ composites increase HER rates from the lower log *j*_0_^diam^ (diamagnetic) line to the upper log *j*_0_^spin^ line for metals with unpaired electrons. On paramagnetic Pt, HER rate is not altered by addition of magnetized microparticles (open blue circle).

Experiments identify HER electrocatalysis as critically dependent on magnetic gradients. An externally applied strong but uniform magnetic field does not alter voltammetric responses for Nafion films and magnetized composites on electrodes. The magnetic gradient about magnetized microparticles enhances current and rate.

Concisely, electrocatalytic rates for HER are impacted by inherent magnetic properties of the electrode. From Figure 1, the magnetic effect is binary, on or off. For diamagnetic electrodes with no net electron spin, slow electron transfer rates increase substantially on introduction of magnetized microparticles. Magnetized microparticles establish magnetic gradients at the electrode. Voltammetry for hydrogen evolution at diamagnetic GC electrodes is markedly enhanced at magnetized composites; at paramagnetic Pt electrodes, voltammetry is unchanged. Fundamental and technological implications are significant. Magnetic effects on electron transfer are an as yet unexplored opportunity to probe and advance electron transfer theory, to further understanding of kinetics, and to inform design of magnetoelectrocatalysts.

## Hydrogen Evolution on Metal Electrodes

Hydrogen evolution is an exemplar of electron transfer reactions (eq 1). HER on platinum defines the standard potential for the normal hydrogen electrode (NHE), against which thermodynamic values are reported. HER is important in water splitting, electrolysis, and other energy significant reactions. Fundamentally, HER is critical to understanding electrode reactions and electrocatalysis.

However, HER is not simple kinetically. Proton adsorbs to the metal; the elementary electron transfer is between metal and adsorbed cation H^+^ and atom H^•^; two adsorbed H^•^ form adsorbed H_2_; and H_2_ desorbs.^6^ Electrochemical rate constants of standard heterogeneous rate *k*^0^, exchange current density *j*_0_, and transfer coefficient α are determined from experimental current voltage data as detailed in SI.2.1.^7^

Electron transfer reactions at the electrode electrolyte interface (O + e ⇄ R) are characterized by potential dependent interfacial rate constants *k*_f_(*E*) and *k*_b_(*E*) for the reduction and oxidation. Current density *j*(*E*) (A/cm^2^) is measured as a function of potential applied to the electrode, *E* (V). *E* is reported relative to the standard potential *E*^0^, formal potential *E*^0^′ for specified nonstandard conditions, or equilibrium potential *E*_eq_. *E*_eq_ is measured at open circuit potential (OCP) where there is no net current flow. For O + e → R, *k*_f_(*E*) = *k*^0^ exp[−α*f*(*E* – *E*^0^′)]. For the oxidation, *k*_b_(*E*) = *k*^0^ exp[(1 – α)*f*(*E* – *E*^0^′)], where *f* = *F*/*RT*.^7^ For only O present at concentration c* (mol cm^–3^) and small voltage perturbations where *E* – *E*^0^′ ≳ 0, eq 2 applies to the onset potentials at low current density. The current is set by electron transfer kinetics with no mass transport limitations.
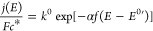
2*k*^0^ (cm/s) is measured at *E* = *E*^0^′. The free energy of activation to form the transition state is Δ*G*^⧧^. The transfer coefficient α = *F*^–1^ ∂Δ*G*^⧧^/∂*E* partitions the electrical component of the Δ*G*^⧧^ between O + e and R; 0 ≤ α ≤ 1 with α typically ≈0.5.

*j*(*E*) is often more easily measured relative to *E*_eq_. Analogous to eq 2, the rate constant is the exchange current density *j*_0_.
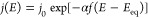
3*j*_0_ (A cm^–2^) is measured at *E* = *E*_eq_. Equating *j*(*E*) of eqs 2 and 3, electrocatalytic rates *k*^0^ and *j*_0_ are related.
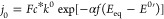
4

### HER Rates on 31 Metal Electrodes: Log *j*_0_ Versus Φ

Trasatti compiled *j*_0_ and Φ for HER at 31 metal electrodes (SI.1 and Table SI.1).^1–3^ Rates were measured near *E*_eq_ (eq 3) at room temperature with polycrystalline metal electrodes in aqueous strong acids at pH 0. Work function is the energy to remove an electron from the surface of a material far from the surface into vacuum. Trasatti’s estimate of Φ as the energy to move an electron from the metal electrode surface to a redox species immediately at the electrode electrolyte interface yields the two parallel lines in Figure 1. Trasatti labeled the upper line for d metals and the lower line for sp metals. Platinum, an excellent electrocatalyst for HER, has the highest *j*_0_ at the largest Φ. The lowest rate, off line, is for mercury, historically favored as a polarographic electrode in water because poor HER rates on Hg widen the assessable voltammetric window. Gold is a good electrode for HER, but 200-fold lower *j*_0_ than ruthenium with comparable Φ.

In Figure 1, only diamagnetic metals with no unpaired electrons are on the lower line. The upper line captures only metals with unpaired electrons and inherent spin. Paramagnetic, ferromagnetic (Fe, Co, and Ni), and antiferromagnetic (Cr) metals have unpaired electrons. Paramagnetic platinum group metals (PGMs) are the best HER electrocatalysts. Au, the best diamagnetic electrocatalyst, has a 10^3^ lower rate than paramagnetic PGMs.

#### Linearity and Regressions of Log *j*_0_ with Φ

The exponential increase of *j*_0_ with Φ arises because Φ directly lowers Δ*G*^⧧^ for HER.^8^ This accounts for the linearity of Figure 1 and explains why highest log *j*_0_ is at highest Φ. Electrode metals with unpaired electrons and net spin have higher rates, *j*_0_^spin^. Linear regression for the higher rate line yields log *j*_0_^spin^ = (6.4_4_ ± 0.2_4_)Φ(eV) – (35._4_ ± 1._1_) with *R*^2^ = 0.98. Diamagnetic metals with no unpaired electrons have lower rates, where log *j*_0_^diam^ = (6.5_6_ ± 0.5_6_)Φ(eV) – (38._5_ ± 2._4_) with *R*^2^ = 0.93 (excludes Hg). With 95% confidence, the slopes do not differ.

The difference in the intercepts yields Δlog *j*_0_ = *j*_0_^spin^ – *j*_0_^diam^ of 3._1_ ± 2._6_. For a given Φ, *j*_0_^spin^ = (1_.26_ × 10^3^)*j*_0_^diam^, ±32%. Alternatively, for a given log *j*_0_, the difference in the work functions Φ^spin^ – Φ^diam^ is −0.48 eV or ≈ −46 kJ mol^–1^. The energy difference of Δlog *j*_0_ is not negligible. Viewed as an Eyring rate expression (*k* = *A*′ exp[−Δ*G*^⧧^(*RT*)^−1^], eq SI.1) with comparable pre-exponential factors (SI.2.1), Δlog *j*_0_ ≈ 3 for HER suggests that Δ*G*_spin_^⧧^ is ≈17 kJ mol^–1^ lower energy than Δ*G*_diam_^⧧^. As Δ*G* = −*F*Δ*E*, this corresponds to a potential shift of ≈+180 mV (≈180 mV lower overpotential). This estimates that electrodes with electron spin magnetoelectrocatalyze HER at approximately 17 kJ mol^–1^ or 0.18 eV lower energy than diamagnetic electrodes for the same Φ. This energy is well above Zeeman energies for spectral line splittings in a magnetic field and comparable to the energy of the hydrogen bond in the Zundel cation (H_5_O_2_^+^) of 18.4 kJ mol^–1^.^9^ The energy difference between the upper and lower rate lines and the intercepts of Figure 1 arises from the magnetic properties of the electrode.

Of the 31 metals, one case of three metals with a common Φ is found. Three metals Ag, Zn, and Mo with Φ = 4.3 eV yield linear correlation (*R*^2^ = 0.997) of log *j*_0_ with molar magnetic susceptibility χ_m_ of the metal (SI.1.2, Figure SI.1). Paramagnetic Mo sustains higher *j*_0_ than diamagnetic Ag and Zn. For common Φ, *j*_0_^spin^ ≈ 10^3^*j*_0_^diam^.

#### Binary Segregation with Magnetic Properties of Electrode Metals

Electron spin and so magnetic properties of the electrode segregate HER electron transfer rates into two groups (Figure 1). Magnetic impact of the metal is binary, either on or off. If the metal has inherent electron spin, the rate is 10^3^ higher than a metal with the same Φ but all electrons paired (diamagnetic). No obvious dependence on the number of unpaired electrons is noted. The stark, binary segregation of the data in Figure 1, is without exception and substantiates a magnetic effect on electron transfer.

## Magnetized Microparticles on Diamagnetic GC and Paramagnetic Pt Electrodes

To vet the magnetic impact on electrocatalysis and electron transfer rate, diamagnetic GC electrodes are modified with magnetic microparticles. Experimental outcomes substantiate that there is a magnetic effect on electrocatalysis; that magnetoelectrocatalysis can be induced at diamagnetic electrodes; that HER at paramagnetic Pt electrodes is not enhanced with magnetized microparticles; and that magnetic gradients rather than uniform magnetic fields induce enhanced HER kinetics.

### Experimental Methods

Diamagnetic GC and paramagnetic Pt disks are modified with Nafion films or composites of Nafion and iron oxide microparticles. All particles are rendered chemically and electrochemically inert by thin siloxane coatings (SI.3.1.2). Ferrimagnetic microparticles can be magnetized and demagnetized. Magnetized and demagnetized composites are chemically the same but differ physically in the presence and absence of magnetic fields and gradients. Magnetized and demagnetized composites and diamagnetic Nafion films are compared. Linear sweep voltammetry (LSV) in strong acid tracks HER onset at low current densities where eq 3 applies. See SI.3 and SI.4.

#### Iron Oxide Microparticles

Iron oxide microparticles (SI.3.1.2) are either commercially available in 1 μm microspheres (Chemicell, SiMag-CX®) that contain maghemite (γ-Fe_2_O_3_) nanoparticles or larger 5 μm in-house, solid core magnetite (Fe_3_O_4_) microparticles (SI.3.1.2).^5,10^ γ-Fe_2_O_3_ and Fe_3_O_4_ microparticles are magnetized inside a hollow cylinder rare earth magnet and demagnetized on mild agitation (SI.3.1.2). Bulk ferrimagnet properties are shown in Table SI.2. SiMag-CX particles are γ-Fe_2_O_3_ nanoparticles in alkylsiloxane. CX denotes the number of alkyl carbons (i.e., C1 methyl, C3 propyl, and C8 octyl). CX microspheres and Nafion are measured in a Guoy balance (SI.3.1.2 and Table SI.3). Nafion is diamagnetic. CX magnetic content is reported as volume magnetic susceptibility χ_v_. For γ-Fe_2_O_3_ microspheres, χ_v_ ranks as C1 > C3 > C8. Smaller 1 μm γ-Fe_2_O_3_ microparticles contain less iron than the 5 μm solid core Fe_3_O_4_ microparticles and sustain a weaker field.

#### Nafion Films and Microparticle Composites

GC and Pt disk electrodes (Pine Instruments, 0.45 cm^2^) are polished with successive grit alumina and modified with either Nafion films or composites of Nafion and iron oxide microparticles (SI.3.1.3).^4,5^ Nafion is a nanostructured, biphasic cation exchange polymer that concentrates proton but contains no bulk solvent domains (SI.3.1.1).^11^ Nafion films are cast from a Nafion suspension (Aldrich). Composites are cast from a mixture of Nafion suspension and microparticles (SI.3.1.3). After casting, solvents evaporate, modifying layers are 5-7 μm thick. Composites contain 15 or 20% (v/v) particles, as noted in Table 1. Microparticles are magnetized in a hollow cylinder rare earth ring magnet during casting; microparticles are demagnetized before casting.

**Table 1:** Summary of the Experimental Results Shown for Electrodes Modified with Nafion and Microparticles in Nafion^d^

Electrode	Microparticles^a^	*E*_*Naf*_ (V vs SCE)	*E*_*mag*_ (V vs SCE)	Δ*E*(V) = *E*_*mag*_–*E*_*Naf*_	Δ*G* = −*F*Δ*E* (kJ/mol)	j_0_^mag^ / j_0_^Naf^ | _α = 0.5_^b^
GC	γ -Fe_2_O_3_: C1, 15%	-(0.82_0_ ± 0.01_9_)	–0.629 ± 0.001	0.19_1_ ± 0.01_9_	–18._4_	40.
GC	γ -Fe_2_O_3_: C3, 15%		–0.69_7_ ± 0.01_3_	0.12_3_ ± 0.02_3_	–11._9_	11.
GC	γ -Fe_2_O_3_: C8, 15%		–0.70_9_ ± 0.02_1_	0.11_1_ ± 0.02_8_	–10._7_	8.7
GC	Fe_3_O_4_: 5 μm, 15%	–0.7_5_	–0.4_3_	0.28 ±0.01	–27	230
Pt/N_2_	γ -Fe_2_O_3_: C1, 15%	-(0.257 ±0.005)	-(0.250 ±0.002)	0.007 ± 0.005		
Pt/H_2_	Fe_3_O_4_: 5 μm, 20%	–0.252	–0.251	0.001		1
Pt/H_2_^OCP,c^	Fe_3_O_4_: 5 μm, 20%	–0.255^c^	–0.256^c^	-(0.001 ± 0.001)		1

a Microparticles magnetized; microparticle composition is % v/v.

b Calculated for α = 0.5

c Open circuit potential measured in H_2_ purged 1.0 M HNO_3_; precise to ±1 mV.

d LSV for HER onset Potentials for Nafion films and magnetized composites *E*_*Naf*_ and *E*_*mag*_ are measured at current density of 0.4 mA/cm^2^. Potentials are reported vs SCE. All microparticles are magnetized. Uncertainties for diamagnetic electrodes are standard deviations for at least three replicate electrodes. Electrolyte is HNO_3_ at 0.10 M for the γ-Fe_2_O_3_ composites and 1.0 M for the Fe_3_O_4_ composites.

#### Linear Sweep Voltammetry

Electrochemical measurements (SI.3.2) are performed in a three electrode cell. The counter electrode is a high surface area platinum mesh and the reference electrode is saturated calomel electrode (SCE). For γ-Fe_2_O_3_ (CX) composites, the electrolyte is 0.10 M nitric acid sparged with N_2_ gas for 20 min before analysis of each electrode and maintained under a N_2_ blanket; electrodes are equilibrated in solution for 24 h prior to the first scan and re-equilibrated for 1 h between each subsequent scan; and LSV is performed at scan rate 50 mV/s from 0 to −1.0 V versus SCE. Three electrodes of each type are analyzed, each with triplicate LSV measurements.

Conditions are similar for Fe_3_O_4_ microparticles on GC and Pt except that scans are at 100 mV/s in 1.0 M HNO_3_. At GC, the electrolyte is not degassed. At Pt, the electrolyte is degassed with H_2_.

**Figure 2:**
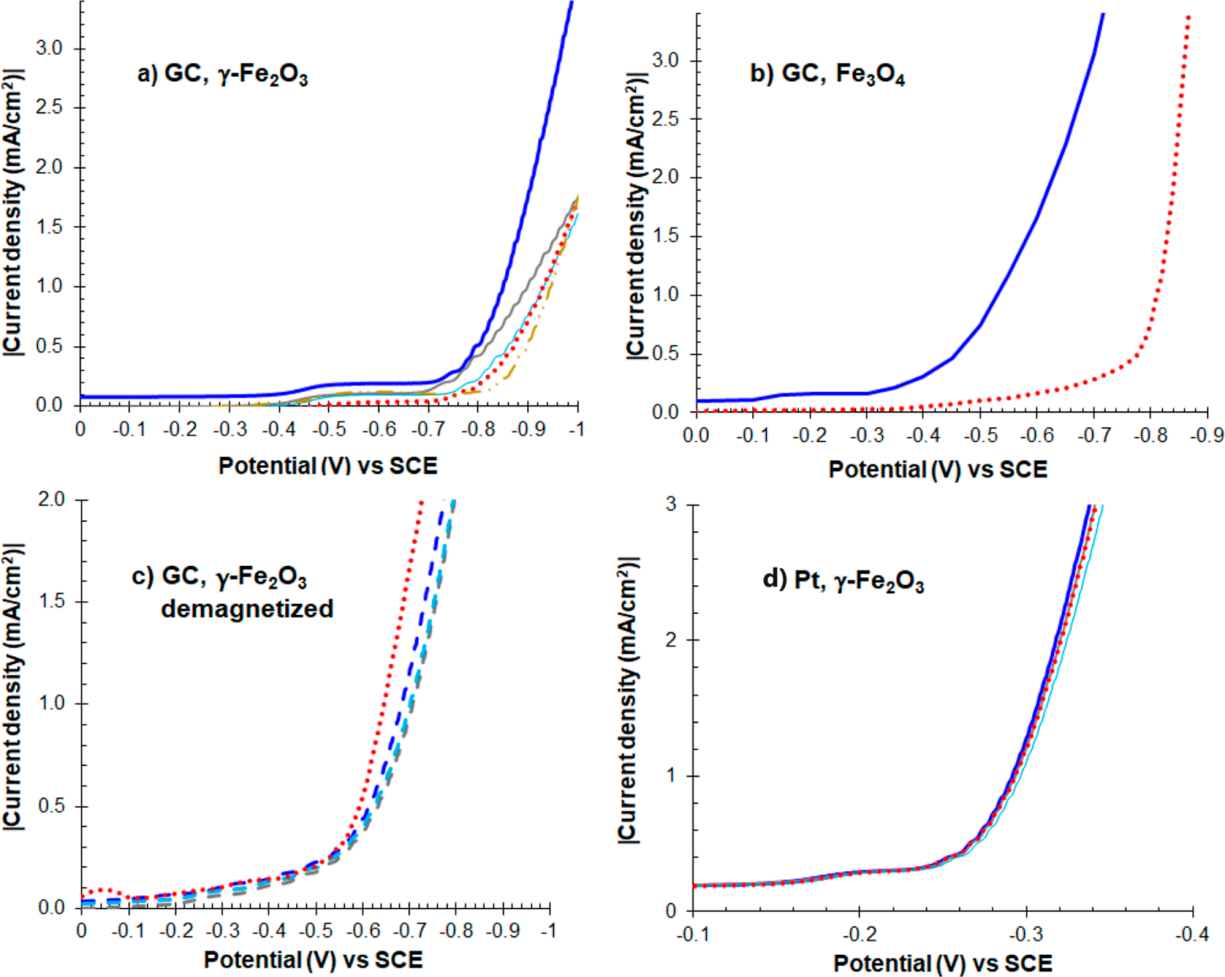
Current density (mA/cm^2^) versus potential (V) for diamagnetic glassy carbon (GC) (a–c) and paramagnetic Pt (d) electrodes modified with composites of magnetized microparticles in Nafion (solid lines), Nafion films (red dotted line), and composites of demagnetized magnetic microparticles in Nafion (dashed lines). Conditions are 0.1 M HNO_3_ with N_2_ blanket at 50 mV/s except for (b) where 1.0 M HNO_3_ at 100 mV/s is used. Composites are 15% v/v, siloxane coated, iron oxide microparticles in Nafion. Current densities are per geometric surface areas. For GC (a,b), onset potentials for magnetized composites (*E*_mag_) are positive of diamagnetic Nafion films (*E*_Naf_). In (c), onsets for demagnetized composites (*E*_demag_) are comparable to or slightly negative of *E*_Naf_. Onset potentials are measured at 0.4 mA cm^–2^, where current is limited by electron transfer. In all cases for GC, the rate is higher at magnetized composites where Δ*E* = *E*_mag_ – *E*_Naf_ > 0 (Table 1). GC is modified with 1 μm γ-Fe_2_O_3_ (CX) microparticles magnetized (a) and demagnetized (c). In (b), GC is modified with magnetized 5 μm Fe_3_O_4_ microparticles. In (a), Δ*E* scales with particle volume susceptibility as C1 (blue) > C3 (olive) > C8 (teal) (Table SI.3). For C1 in (a), Δ*E* ≈ 190 mV; in (b), Δ*E* ≈ 280 mV. Fe_3_O_4_ microparticles introduce stronger magnetic gradients to the GC surface than γ-Fe_2_O_3_ microparticles. In (a,c), the composites are chemically the same and differ only physically with the presence and absence of magnetic gradients; HER rates with magnetized microparticles (a) are higher than for demagnetized particles (c). LSVs for paramagnetic Pt (d) are collected under the same conditions as (a). For current densities of 1 mA cm^–2^ and less, the curves overlay for the Nafion film (red dot) and the magnetized composites of C1 (blue wide solid), C3 (blue medium solid), and C8 (light blue narrow solid), consistent with no impact of magnetic gradients on electron transfer rate at paramagnetic Pt.

Representative LSVs are shown in Figure 2. Data are aggregated in Table 1. Details are given in SI.4 and SI.5.

#### Externally Applied Magnetic Field

A uniform magnetic field (SI.3.3) is generated with a neodymium iron boride (NdFeB) ring magnet (o.d. = 7.6 cm, i.d. = 3.8 cm, 1.3 cm height). LSVs are recorded at 0.432 cm^2^ platinum disk electrodes placed in nitrogen sparged 0.64 mM tris(2,2′-bipyridine)ruthenium(II) dichloride Ru(bpy)_3_Cl_2_ and 0.1 M HNO_3_. Replicate measurements (SI.3.3) are made for 7 μm thick Nafion films, 7 μm thick magnetized C1 composites, 7 μm thick demagnetized C1 composites, and unmodified Pt at 20, 50, 100, and 200 mV/s. Measurements are made without the external ring magnet and repeated with the cell centered in the NdFeB ring magnet (Figure SI.2). Centered in the ring magnet, a strong, uniform magnetic field is generated and magnetic gradients are minimized.

#### Data Analysis

Voltammetric responses are dominated by electron transfer kinetics at lower current densities and by mass transport at higher current densities (SI.2.2). To estimate relative HER rates, differences in onset potentials for electrodes modified with magnetized composites *E*_mag_ and Nafion films *E*_Naf_ are determined at a low, fixed current density of 0.4 mA cm^–2^, where eq 3 applies (SI.2.2). For all diamagnetic electrodes, Δ*E* = *E*_mag_ – *E*_Naf_ > 0. Less extreme applied potentials are needed to drive HER for magnetized composites than Nafion films.

Several diagnostics to compare rates with and without magnetized microparticles are derived from Δ*E* (SI.2.2). Briefly, the energy for electron transfer is decreased as Δ*G* = −*F*Δ*E*. Relative exchange current densities are α^–1^ log[*j*_0_^mag^/*j*_0_^Naf^] = −(*f*/2.303)Δ*E* (eq SI.9). *j*_0_^mag^/*j*_0_^Naf^|_α=0.5_ denotes *j*_0_^mag^/*j*_0_^Naf^ at α = 0.5 (eq SI.10). Note, *j*_0_^mag^/*j*_0_^Naf^ = *k*_mag_^0^/*k*_Naf_^0^ (eq 4). Data are summarized in Table 1.

### HER at Magnetically Modified Diamagnetic GC and Paramagnetic Pt Cathodes

For diamagnetic GC cathodes, representative LSVs are shown in Figure 2a–c. Magnetized composites (solid lines) electrocatalyze HER more efficiently than diamagnetic Nafion films (red dots). Demagnetized composites (short dashes) on GC (Figure 2c) electrocatalyze HER at rates comparable or slightly lower than Nafion films. Magnetized composites support more facile HER electrocatalysis than chemically identical demagnetized composites on diamagnetic GC. In Figure 2d, LSVs for Pt modified with Nafion and magnetized γ-Fe_2_O_3_ (C1, C3, and C8) composites superimpose in the kinetically controlled region at low current densities. Rates on paramagnetic Pt are unchanged on introduction of magnetic gradients (Table 1 with details in the Supporting Information).

#### Diamagnetic Glassy Carbon Electrodes

Diamagnetic GC is a poor HER electrocatalyst. LSVs for modified GC electrodes are shown in Figure 2 for (a) magnetized and (c) demagnetized γ-Fe_2_O_3_ C1, C3, and C8 composites^4^ and for (b) Fe_3_O_4_ composites.^5^ Details are listed in SI.4. Hydrogen evolves at rates higher with magnetized composites than those with Nafion films (SI.4), as shown by more positive onset potentials (lower overvoltage) for fixed *j*(*E*) or higher *j*(*E*) for fixed *E*.

##### Variation of Magnetic Content of γ-Fe_2_O_3_ Microparticles

By various measures, rates at GC increase with the magnetic content of CX microparticles. For magnetized γ-Fe_2_O_3_ composites (Figure 2a), rate increases with magnetic content as C1 > C3 > C8 > Nafion film (Table SI.3). Δ*E* scales linearly with measured volume magnetic susceptibility χ_v_ (Figure SI.5, SI.3.1.2). Δ*G* decreases with magnetic content as Δ*G* (kJ/mol) = −1.14 × 10^6^ χ_v_(μcgs) with *R*^2^ = 0.998_7_. For magnetized C1, C3, and C8 composites, Δ*E* values of +190, +123 and +111 mV enhance rate *j*_0_^mag^/*j*_0_^Naf^|_α=0.5_ by 40., 11., and 8.7 fold. *j*_0_ increases exponentially with magnetic content (Figure SI.5).

##### 1 μm γ-Fe_2_O_3_ and 5 μm Fe_3_O_4_ Microparticles

Voltammetric morphologies for magnetized Fe_3_O_4_ (Figure 2b) and γ-Fe_2_O_3_ (Figure 2a) composites are similar. Magnetized C1 composites on GC yield Δ*E* = 0.191 ± 0.019 V; Δ*G* = −18.4 kJ mol^–1^; α^–1^ log(*j*_0_^mag^/*j*_0_^Naf^) ≈ 3.2; and *j*_0_^mag^/*j*_0_^Naf^|_α=0.5_ ≈ 40. See Table 1. Effects are greater for magnetite microparticles. Larger 5 μm Fe_3_O_4_ microparticles introduce more unpaired spins to the electrode surface than smaller 1 μm γ-Fe_2_O_3_ particles (SI.6.4). For magnetized Fe_3_O_4_ composites (Figure 2b), Δ*E* = 0.28 V with Δ*G* of −28 kJ mol^–1^, α^–1^ log(*j*_0_^mag^/*j*_0_^Naf^) ≈ 4.7, and *j*_0_^mag^/*j*_0_^Naf^|_α=0.5_ is 230.

In Figure 1, magnetized C1 composites substantially increase log *j*_0_ (blue arrow), but magnetized Fe_3_O_4_ increase log *j*_0_ from the log *j*_0_^diam^ line to the log *j*_0_^spin^ line (gray arrows). (Addition of GC data to Figure 1 is detailed in SI.4.1.). Magnetized Fe_3_O_4_ composites on GC sustain electrocatalysis comparable to Fe and W, with similar Φ.

##### Magnetized and Demagnetized Composites

GC electrodes are modified with composites of Nafion and γ-Fe_2_O_3_ microparticles demagnetized by gentle agitation (SI.3.1.2). LSVs are similar for Nafion and demagnetized composites (Figure 2c), but microparticles block electrode access, and a small lag (Δ*E*_demag_ = *E*_demag_ – *E*_Naf_ ≲ 0) results (SI.4). For C1 composites, *E*_demag_ is −0.828 V vs SCE. Magnetized composites (Figure 2a and Table 1) sustain higher HER rates than demagnetized composites (Figure 2c). Although the chemical composition is the same, statistical confidence is >99.9% that log *j*_0_ differs for magnetized and demagnetized composites on GC.

##### Other Diamagnetic Cathodes and Photocathodes

Other diamagnetic cathodes and photocathodes exhibit voltammetric behavior similar to that of GC. In all cases, Δ*E* > 0. Smallest enhancements are for Au.^4^ Hg pool has the largest enhancements and largest standard deviations because of mechanical instabilities.^4^ The Fe_3_O_4_ modified electrodes are GC (Table 1) and n-GaAs and p-Si photocathodes irradiated with a solar simulator at 20 mW cm^–2^. Electrodes modified with magnetized 5 μm Fe_3_O_4_ composites have similar Δ*E* values of 270–280 mV.^5^

#### Paramagnetic Platinum Electrodes

In contrast to diamagnetic electrodes, LSVs for the HER at paramagnetic platinum electrodes are the same for magnetized composites and diamagnetic Nafion films. No magnetic impacts are observed for paramagnetic Pt. Three experiments are detailed in SI.5: LSV under a nitrogen blanket, LSV under a hydrogen blanket, and the OCP measurements under a hydrogen blanket.

##### LSV at Pt under N_2_ with γ-Fe_2_O_3_

LSV in N_2_ degassed 0.10 M HNO_3_ at Pt disks modified with Nafion films and magnetized 15% v/v γ-Fe_2_O_3_ C1, C3, and C8 composites superimpose in the kinetically controlled range below 1 mA cm^–2^ (SI.5.1.1, SI.6, and Figure 2d).^4^ At higher current densities and longer times, magnetic field dependent transport in the electrolytes spreads LSV currents once the diffusion length exceeds film thickness. In Table 1 for replicate measurements, Δ*E* = *E*_mag_ – *E*_Naf_ is +(0.007 ± 0.005) V. With >90% confidence, *E*_mag_ and *E*_Naf_ are not statistically different.

##### LSV at Pt under H_2_ with Fe_3_O_4_

LSV in H_2_ degassed 1.0 M HNO_3_ at Pt disks modified with a Nafion film and a magnetized 20% v/v Fe_3_O_4_ composite overlay in the kinetically controlled range below 1.5 mA cm^–2^ (Figure SI.7 in SI.5.1.2).^5^ Once corrected for background currents and electrode area blocked by dense Fe_3_O_4_ microparticles that settle on the electrode (SI.5.1.2), *E*_Naf_ = −0.252 V and *E*_mag_ = −0.251 V vs SCE with measurement uncertainty of 1 mV. In Table 1, Δ*E* = +0.001 V, which is not statistically different from zero.

##### OCP under H_2_ with Fe_3_O_4_

Measured with no net current flow, OCPs determine the equilibrium potentials. For Nafion films and magnetized 20% (v/v) Fe_3_O_4_ composites on Pt under H_2_ in 1.0 M electrolyte, open circuit potentials of −0.255 and −0.256 V vs SCE are found. With measurement precision of 1 mV, Δ*E* = −(0.001 ± 0.001) V (Table 1 and SI.5.2).^5^

### Uniform External Magnetic Field: Negligible Impact of Ring Magnet

To differentiate impacts of magnetic fields and magnetic gradients, the electrochemical cell is centered in a hollow cylinder rare earth permanent magnet (Figure SI.2) to establish a strong uniform field perpendicular to the electrode surface.^4^ Voltammograms are collected in 0.64 mM Ru(bpy)_3_Cl_2_ and 0.1 M HNO_3_ at an unmodified Pt electrode and at Pt electrodes modified with Nafion films and composites of either magnetized or demagnetized C1 γ-Fe_2_O_3_ microparticles. Details about the experiment and voltammetry of Ru(bpy)_3_^2+^ in Nafion are given in SI.3.3, SI.6, and Table SI.6.

**Figure 3:**
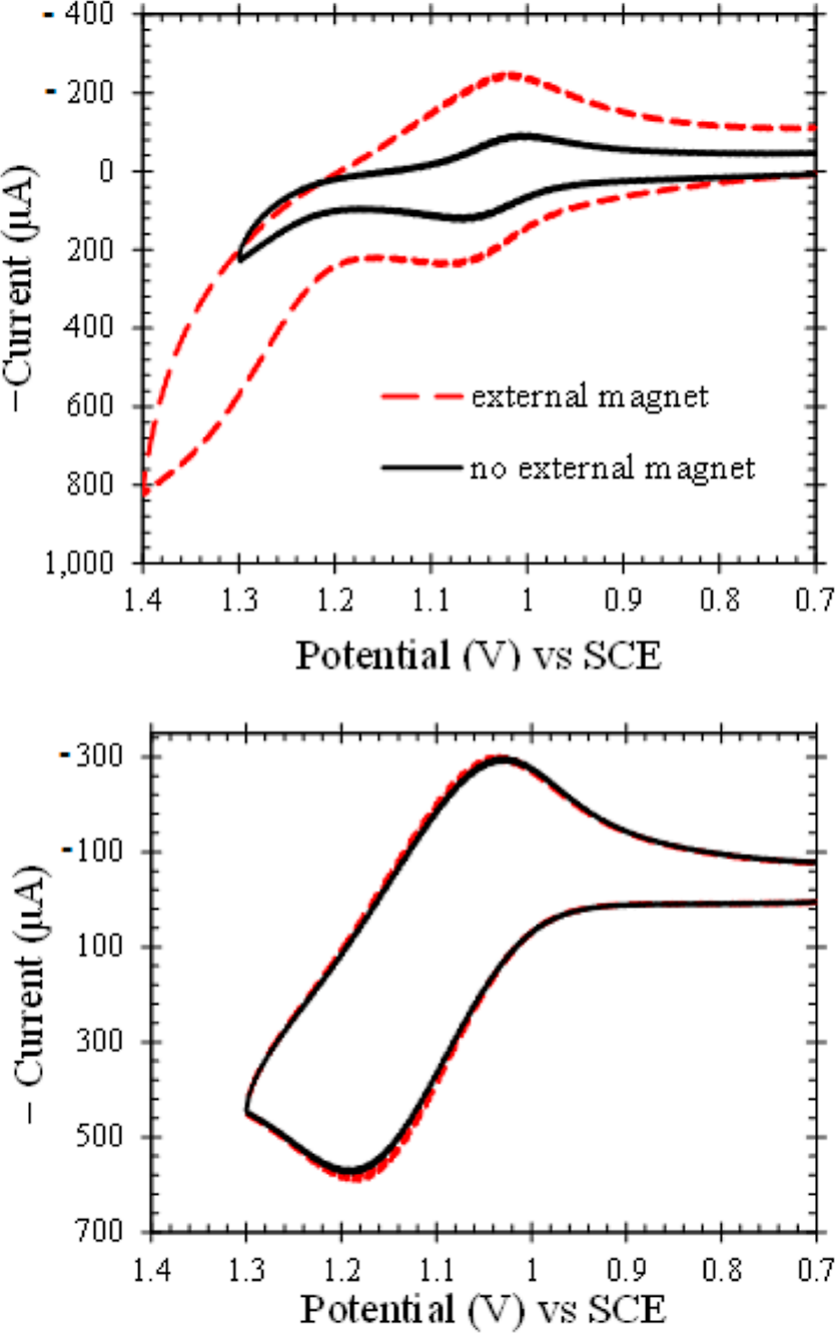
Cyclic voltammograms for 0.64 mM Ru(bpy)_3_^2+^ in 0.10 M HNO_3_ at 200 mV/s are shown for a Pt disk (0.452 cm^2^) that is unmodified (top) and modified with a Nafion film (bottom), recorded without (black) and with (red dashed) the external rare earth ring magnet. On application of the strong uniform external field, current is enhanced for the unmodified electrode consistent with magnetohydrodynamic mass transport of H^+^, NO_3_^–^, Ru(bpy)_3_^2+^, and Cl^–^ in the bulk solvent. Current at >1.2 V vs SCE is chloride oxidation. For the electrode modified with a Nafion film, the current response is unchanged on introduction of the uniform external field. The uniform magnetic field does not impact electron transfer rate or mass transport in nanostructured Nafion.

For the unmodified Pt disk, the uniform field of the external ring magnet enhances current near 1.05 V vs SCE by 65% (Figure 3 top). The uniform magnetic field interacts with ions mobile in the electrolyte to generate a Lorentz force that induces bulk fluid flow and enhances current through mass transport.^12–14^

**Figure 4:**
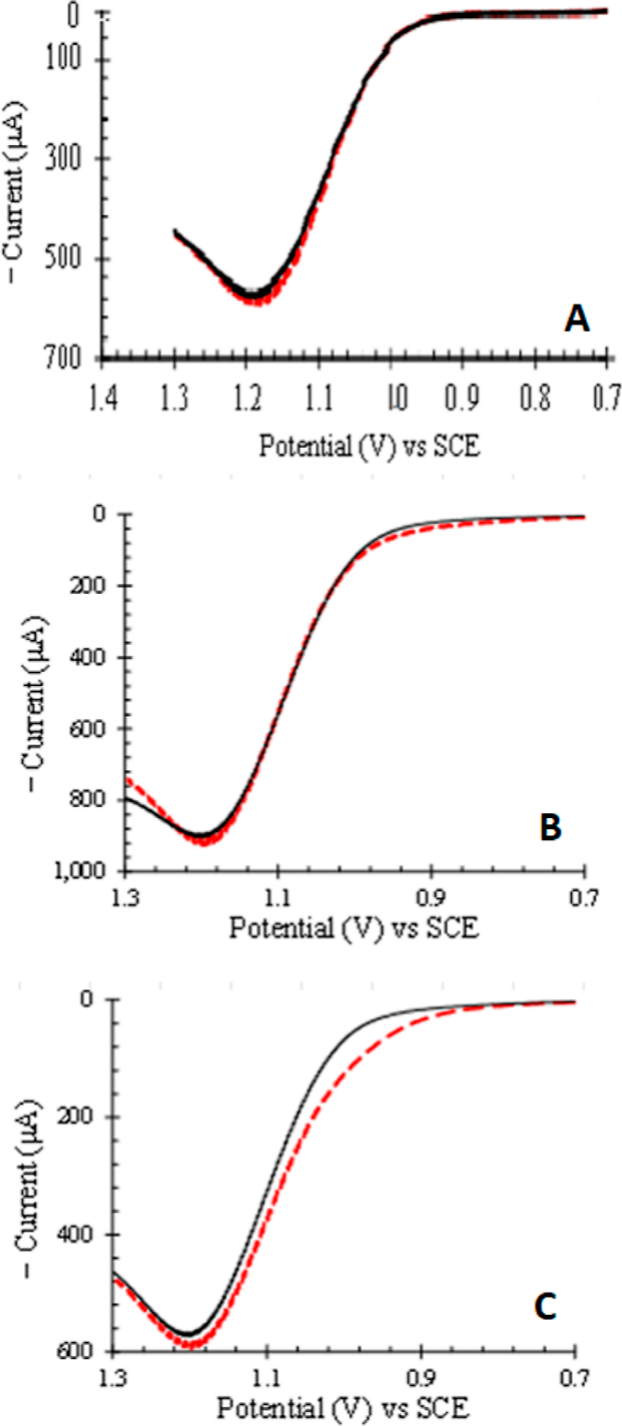
LSVs for 0.64 mM Ru(bpy)_3_^2+^ in 0.10 M HNO_3_ at 200 mV/s are shown for a Pt disk (0.452 cm^2^) modified with a Nafion film (A), a magnetized C1 composite (B), and a demagnetized C1 composite (C), without (black) and with (red dashed) the external ring magnet. With no ring magnet, peak current for the magnetized composite (B) is higher than for the Nafion film (A) and demagnetized composite (C) because of magnetic gradients about the microparticles in (B). With the external ring magnet, LSVs for Nafion film and the magnetized composite are not altered within the width of line. For the demagnetized composite, the peak current is increased ≲5%. Electron transfer rate in Nafion films and composites is not impacted by the uniform magnetic field.

For the Nafion filmed Pt electrode, CVs with and without the external magnet superimpose (Figure 3 bottom). The uniform external field does not impact the electron transfer kinetics or mass transport in the Nafion film. In Figure 4, LSVs for a Nafion film and a magnetized C1 composite on Pt are not altered by the uniform field. The higher peak currents for the magnetized composite are set by the gradient magnetic field about the microparticles and not the uniform field. For the demagnetized composite, the uniform field enhances peak current ≲5%, perhaps due to slight magnetization of the demagnetized particles by the external magnet (Figure 4). Peak currents for Nafion and the demagnetized composites are not statistically different.

## Discussion

Thermodynamically, energies of magnetic fields are negligible compared to ambient thermal energies. Magnetic effects are driven by the gradients that arise in dynamics. Dynamics include chemical reactions, mass transport, and electron transfer. Spin effects in radical reactions are well established.^15–20^ Magnetic field effects on chemical reactions have been considered by Turro and Kraeutler,^15^ Steiner and Ulrich,^20^ and Buchachenko.^16^ In electrochemistry, magnetic effects are a long established research domain.^21–32^ Magnetic effects on transport^14^ are well developed for uniform and gradient magnetic fields.^12–14,33–49^ Although a magnetic effect on electron transfer might be anticipated based on the coupling of current and electrical and magnetic fields and gradients in electromagnetic theory, magnetic effects on electron transfer are not well resolved. Careful studies in uniform magnetic fields have identified no impacts on electron transfer for electrode materials with or without unpaired electrons.^50,51^ Here, magnetic gradients are identified as a critical component in the magnetoelectrocatalysis of HER.

### Electrons, Spin, Fields, and Gradients

In the simplest view, magnetic properties and interactions are set by unpaired electron spins in materials, molecular species, and atoms. As magnetic dipoles, unpaired electron spins generate magnetic fields. In diamagnetic species where all electron spins are paired, fields of the spin up and spin down electrons cancel, and a magnetic field is not established. Paramagnetic and ferrimagnetic species have unpaired electron spins. When placed in an external magnetic field, unpaired spins align with the field. When placed in a magnetic gradient, electrons as magnetic dipoles are attracted into the gradient.^52^ Once removed from the external field, spin alignment is lost in paramagnetic species, but ferrimagnetic materials sustain alignment to form permanent magnets. Rare earth magnets are sufficiently strong to magnetize iron oxide ferrimagnets (SI.3.1.2).

Spins are aligned in magnetic fields but unpaired electrons move in magnetic gradients. Gradients are established at interfaces. At the electrode electrolyte interface, a magnetic gradient is established between a paramagnetic metal and a diamagnetic electrolyte. For a diamagnetic metal, a negligible gradient is established, but magnetized composites deployed on diamagnetic electrodes impose interfacial magnetic gradients that catalyze electron transfers.

In the elementary step of HER, an electron transfers between the electrode metal and adsorbed hydrogen species, the proton H_ads_^+^ and the atom H_ads_^•^. As spin aligns with the field, the interfacial magnetic gradients facilitate electron motion across the interface.

The simplistic view of an unpaired electron moving in a magnetic gradient underlies the observations made here. The discussion focuses on establishing that there is a magnetic effect on interfacial electron transfer and that the effect arises through the magnetic gradient rather than through the field.

### Identification of an Inherent Magnetic Effect on Electron Transfer

In Figure 1, the plot of log *j*_0_ with Φ yields two parallel lines. The stark segregation of the two parallel lines into lower rates for diamagnetic electrodes and higher rates for metals with electron spin identifies a magnetic effect on electron transfer. Unpaired electrons establish the magnetic field of the metal, where the field decays across the interface into the electrolyte. A magnetic gradient is established at the interface between the metal and the diamagnetic electrolyte. The gradient about a paramagnetic atom is steep as it drops over ≲1 nm (SI.6.4), the distance between the metal surface and adsorbed hydrogens. The difference in the intercepts for electrodes with spin log *j*_0_^spin^ relative to diamagnetic electrodes log *j*_0_^diam^ identifies *j*_0_^spin^/*j*_0_^diam^ ≈ 1000 for a given Φ. For diamagnetic electrodes, standard rate constants are 17 kJ mol^–1^ higher energy than metals with inherent spin, which corresponds to a higher overpotential tax of about 180 mV to evolve hydrogen.

Derivation of the linear relationship between log *j*_0_ and Φ finds *F*Φ directly lowers the activation energy for the elementary electron transfer step.^8^ Electron spin may similarly impact the activation energy.

From Figure 1, the magnetic impact is binary, either on (upper line) or off (lower line). There is no obvious dependence of log *j*_0_^spin^ on the number of unpaired spins in the metal.

### Magnetoelectrocatalysis Induced at Diamagnetic GC but Not Paramagnetic Pt

An inherent magnetoelectrocatalytic effect is identified in Figure 1. A magnetic effect on electron transfer is induced by modifying the surface of diamagnetic electrodes with magnetized composites. Magnetized iron oxide microparticles deposit electron spin at the electrode surface to establish an interfacial magnetic gradient that substantially increases the electrocatalytic rates. Rates invariably increased where magnetized composites modify diamagnetic electrodes of GC, Au, and Hg pool and photocathodes of n-GaAs and p-Si.^4,5^ Here, details for GC are reported.

#### Magnetized Composites on Diamagnetic GC Electrodes-Enhanced Rates

HER rates are measured by LSV at diamagnetic GC electrodes modified with diamagnetic Nafion films. Magnetized composites on GC sustain higher HER rates, as marked by decreased overpotential for the onset of H_2_ evolution (Figure 2a,b). In all cases, Δ*E* = *E*_mag_ – *E*_Naf_ > 0, summarized in Table 1 as Δ*E*, Δ*G*, and *j*_0_^mag^/*j*_0_^Naf^|_α=0.5_.

For γ-Fe_2_O_3_ microparticles, a higher iron oxide content establishes steeper interfacial gradients (χ_v_, Table SI.3 in SI.3.1.2) that increase Δ*E* as C1 > C3 > C8. Δ*E* scales linearly with χ_v_ (Figure SI.5). For magnetized C1 composites, Δ*E* is +190 mV, Δ*G* is −18._4_ kJ mol^–1^, and *j*_0_^mag^/*j*_0_^Naf^|_α=0.5_ is 40. Magnetized γ-Fe_2_O_3_ microparticles suffice to establish the interfacial gradient, but the gradient is less steep than that established about Fe_3_O_4_ microparticles.

The solid core of the 5 μm magnetite particles provide more unpaired spins than 1 μm γ-Fe_2_O_3_, in part due to density and saturation magnetization (Table SI.2, SI.6.4). For magnetized Fe_3_O_4_ composites, the HER rate is higher; Δ*E* is +280 mV, Δ*G* is −27 kJ mol^–1^, and *j*_0_^mag^/*j*_0_^Naf^|_α=0.5_ is 230. Similar enhancements are found for Fe_3_O_4_ composites on p-Si and n-GaAs photocathodes.

Magnetized composites on the GC increase *j*_0_. For Φ of 4.61 eV for diamagnetic GC,^53^ log *j*_0_^diam^ is estimated as −8.3 (SI.4.1). The relative increases of log *j*_0_ are plotted on Figure 1. Magnetized C1 γ-Fe_2_O_3_ composites (blue arrow) increase the rate substantially (≈40×). Fe_3_O_4_ composites provide a sufficient gradient to shift the rate on Nafion-modified GC from the diamagnetic line to the line for metals with unpaired electrons. With magnetized Fe_3_O_4_ composites, increases in exchange current density approach a thousand fold. Fe_3_O_4_ may approach the upper limit of enhancement to be derived from the introduction of magnetized microparticles.

In Figure 2c, demagnetized CX composites are shown relative to a Nafion film. For demagnetized composites, *E*_demag_ is comparable to or slightly less than *E*_Naf_. There is no evidence of a rate enhancement for the demagnetized composites. Microparticles can settle at the electrode interface during composite formation. Where inert particles block access to the electrode surface, current decreases and *E*_demag_ shifts slightly negative of *E*_Naf_.

Comparison of magnetized and demagnetized C1 composites is important evidence of a magnetic effect on electron transfer and electrocatalysis. Onset potential for magnetized composites *E*_mag_ is positive of the demagnetized composites *E*_demag_ by +198 mV. Hydrogen evolves at magnetized composites at 19. kJ mol^–1^ lower energy than demagnetized composites. For α of 0.5, *j*_0_^mag^ ≈ 47*j*_0_^demag^. Magnetized and demagnetized composites are chemically the same and differ physically only in the presence and absence of magnetic fields and gradients. Magnetic gradients increase the electrocatalytic rate.

#### Magnetized Composites on Paramagnetic Pt Electrodes: No Effect

No statistically significant impacts on Pt voltammetry are found for magnetized composites (Figure 2d, Table 1 and SI.5). For magnetized C1 γ-Fe_2_O_3_ composites on Pt in N_2_ sparged 0.1 M HNO_3_ (Figures 2d and SI.6, SI.5.1.5), LSV currents overlay Nafion and demagnetized composites at low current densities. In 1.0 M HNO_3_ under a hydrogen blanket, Nafion and magnetized Fe_3_O_4_ composites overlay within 1 mV (Figure SI.7, Table SI.4, SI.5.1.2). Paramagnetic Pt has unpaired spins and addition of unpaired spins with magnetized microparticles does not impact rate under these conditions (SI.5.1.2). To assess any impact on thermodynamics, a magnetized Fe_3_O_4_ composite and Nafion film are compared under a hydrogen blanket in 1.0 M HNO_3_. The equilibrium OCP measurements yield Δ*E* of −(0.001 ± 0.001) V. For these conditions, neither dynamics nor thermodynamics for HER on Pt are changed on addition of magnetized microparticles.

#### Electron Transfer: Not Mediation, Not Mass Transport

Magnetic effects arise not through thermodynamics but through the dynamics of mass transport and the kinetics of chemical reactions and electron transfer. Because there is no effect of magnetic gradients on Pt, chemical mediation and magnetically driven mass transport effects do not impact current.

##### Not Mediation

No evidence of iron leaching from the particles has been found either electrochemically or colorimetrically (SI.3.1.2). Thermodynamically, neither iron species nor oxygen can mediate formation of H_2_ from H^+^ (SI.5.3.2). If mediation by chemical species are increasing the HER rate, the effect would be observed for Pt when iron oxide composites are compared to Nafion films. If chemical mediation were increasing the rate, demagnetized composites on GC would be expected to be comparable to or better than Nafion, but in all cases, Δ*E*_demag_ ≤ 0. Chemical species in the iron oxide particles do not increase rate by mediation.

##### Not Mass Transport

Magnetic fields and gradients impact mass transport through interaction with the charge and spin of chemical species. Magnetohydrodynamics (MHD) describes interaction of magnetic fields with ion flux in solution to generate a Lorentz force that alters bulk fluid flow.^12–14^ Gradient magnetic fields are shown to interact with spin and charge to disrupt and enhance mass transport in fluids.^14,33–49^ Magnetically driven mass transport occurs in bulk solvent.^14^

Because voltammetry on Pt does not differ for Nafion and magnetized iron oxide composites, magnetically driven mass transport does not increase current. Nafion is a perfluorosulfonic acid polymer that segregates into a nanostructure of fluorocarbon and water filled domains (SI.3.1.1).^11,54,55^ Bulk fluid transport is incompatible with the nanostructure of Nafion because Nafion contains no bulk solvent. Overlay of LSV on Pt with and without magnetized microparticles discriminates against magnetically driven transport in the Nafion matrix.

### The Gradient is Critical.

The data establish a magnetic effect on electron transfer where either the electrode metal provides unpaired electron spins or magnetized microparticles introduce unpaired spins to diamagnetic electrodes. Unpaired spins establish magnetic fields and associated interfacial magnetic gradients. To differentiate effects of magnetic fields and magnetic gradients, voltammetry in a uniform external field is evaluated. The uniform field is applied with a ring magnet (see section Uniform External Magnetic Field: Negligible Impact of Ring Magnet).

In Figure 3, cyclic voltammograms for an unmodified Pt disk and a Nafion-filmed Pt disk are compared. For the unmodified electrode, the uniform field enhances current for Ru(bpy)_3_^2+^ and Cl^–^ in solution through MHD as a Lorentz force on charge induces bulk solvent motion. Nafion contains no bulk fluid, but Nafion concentrates and electrostatically binds cationic Ru(bpy)_3_^2+^ (SI.3.3.1). For the Nafion filmed electrode, voltammograms superimpose and there is no impact of the uniform external field on either mass transport or electron transfer rates.

In Figure 4, LSV for Pt modified with Nafion (A), magnetized C1 composites (B), and demagnetized C1 composites (C) are shown. Without the external ring magnet, Nafion and demagnetized composites have comparable peak currents. Magnetized composites sustain 50% higher current than Nafion (Table SI.6). In the uniform field of the ring magnet, LSVs for Nafion and the magnetized composite are unaffected (Figure 4). The demagnetized composite is slightly enhanced, perhaps as the uniform field magnetizes a small fraction of the microparticles. The results are consistent with prior studies^50,51^ that found no impact of uniform fields on electron transfer.

These results identify the magnetic gradient rather than the magnetic field as driving HER magnetoelectrocatalysis. Uniform fields align electron spins but electrons move in the gradients.

### Gradients, Electron Transfer, and Magnetoelectrocatalysis

Electron transfer is a dynamic process. At an electrode, electrons transfer between the electrode metal and redox species immediately at the electrode surface. In the presence of an interfacial magnetic gradient, the electron moves in the gradient and the electron transfer rate increases. Electrode metals with unpaired electrons generate a magnetic gradient that facilitates electron transfer. Gradients about a metal atom are steeper than those about a magnetized microparticle (SI.6.4). Addition of microparticles to a metal with unpaired electrons may have small to negligible impact on the interfacial gradient. On a diamagnetic metal with no unpaired electrons, electron transfer is slower because no gradient is available to facilitate electron transfer at the interface. Addition of magnetized microparticles generates a sufficient interfacial gradient to impact rate.

Several examples of a magnetic effect on electron transfer are noted. From the intercepts in Figure 1, the energetic advantage of the magnetic gradient for HER is 17 kJ mol^–1^ or 180 mV diminution of overpotential or a rate increase of *j*_0_ and *k*^0^ of 10^3^. In all cases, addition of magnetized microparticles to diamagnetic electrodes (GC, Au, Hg pool, and photocathodes p-Si and n-GaAs) increases HER rates.^4,5^ Effective HER electrocatalysts include Pt combined with iron, cobalt, and nickel.^6^ Magnetized microparticles added to electrochemical energy systems, such as fuel cells, batteries, and photoelectrochemical cells, increase efficiency, energy, and power, typically by ≈40%, as reported in the scientific^56–62^ and patent^63–72^ literature.

#### Magnetoelectrocatalysis

Magnetoelectrocatalysis is achieved by manipulation of a physical property of the system rather than by modification of the chemical composition of the catalyst. Despite the same chemical composition of iron oxide microparticles in Nafion on diamagnetic electrodes, the physical distinction of a magnetic field and gradient generated at magnetized microparticles enhances HER rates substantially (Figure 2a–c).

Ideas for design of better electrocatalysts for HER are extracted from Figure 1 and Table 1. From Trasatti’s data,^1^ electrocatalysis is promoted by electrodes with high work functions Φ. The energy *F*Φ is thought to lower the free energy of activation for HER.^8^ From Figure 1, the energy for HER is lower by ≈17 kJ mol^–1^, where magnetic gradients are established at the electrode electrolyte interface. A catalyst with unpaired electrons is a better choice than a diamagnetic catalyst. But, addition of magnetized microparticles to diamagnetic electrocatalysts is an effective alternative. The number of unpaired spins introduced by the magnetized microparticles may scale the magnetoelectrocatalytic impact; an upper limit of rate enhancement is likely given the binary on/off behavior of Figure 1 and common Δ*E* of Fe_3_O_4_ composites. It is noted that although high fields on the order of a few Tesla can be generated with common laboratory tools of macroscopic rare earth magnets and electromagnets, it is unlikely that such magnets can establish a sufficiently steep gradient at the electrode electrolyte interface over a thickness of ∼1 nm (SI.6.4). For HER, magnetized microparticles of iron oxide generate a sufficient magnetic field and associated gradient to substantially increase heterogeneous electron transfer rates, *j*_0_ and *k*^0^. Magnetoelectrocatalysis extends to reactions other than HER.^57,62,65^

## Conclusions

A magnetic effect on electron transfer is identified from literature data for HER.^1^ In Figure 1, the distinction of higher and lower rates (log *j*_0_) maps to the presence and absences of unpaired electrons in the electrode metal. Unpaired electrons set magnetic properties. For a given Φ, metals with electron spin sustain HER rates 10^3^ times higher than diamagnetic electrode metals.

Magnetic effects on chemical systems do not arise through equilibrium thermodynamics at ambient temperatures, but through dynamics of chemical reactions, mass transport, and electron transfer. Dynamics arise through gradients. Unpaired electron spins on the metal electrode establish magnetic gradients at the electrode electrolyte interface. Magnetic gradients increase the electron transfer rate as the electron moves in the gradient. At diamagnetic electrodes, HER rates are substantially increased by deploying magnetized microparticles in Nafion on electrode surfaces (Figure 2, Table 1). Magnetized, siloxane-coated 5 μm Fe_3_O_4_ microparticles in Nafion shift log *j*_0_ from the lower, diamagnetic line to the upper line for metals with unpaired electrons (Figure 1, up arrows). Magnetized and demagnetized 1 μm γ-Fe_2_O_3_ in Nafion are chemically the same, but differ in the presence and absence of magnetic fields and gradients. HER rates are substantially (≈47×) higher with magnetized composites than demagnetized composites. For paramagnetic Pt electrodes, no change in voltammetry or the OCP is observed on addition of magnetized microparticles; dynamics of chemical mediation and magnetically driven mass transport do not increase *j*_0_. LSVs at Nafion films and iron oxide microparticle composites are unaffected by uniform external fields (Figures 3 and 4). Enhanced rates arise through magnetic gradients about metals with unpaired electrons and magnetized microparticles.

Magnetic gradients are a missing piece of the puzzle that is magnetoelectrocatalysis. Catalytic rates are substantially higher at electrodes with unpaired spins either inherent to the metal or introduced with magnetized microparticles. Dynamics are driven by gradients. The unpaired spins establish a magnetic gradient at the electrode electrolyte interface that facilitates electron transfer. Consideration of interfacial magnetic gradients opens unexplored domains of kinetics and electron transfer theory and guides design of magnetoelectrocatalysts.

## Supporting Information Available

HER log *j*_0_ and Φ for metal electrodes; relationships between *j*_0_, *k*^0^, Δ*E*, *j*_0_^mag^/*j*_0_^Naf^, additional details about materials and methods, detailed discussion and analysis of data for GC and for Pt, discussion and data for external, uniform magnetic field (PDF)
