# Supplementary material for: Magnetoelectrocatalysis: Evidence from the Hydrogen Evolution Reaction
Source: ACS Phys Chem Au. 2024 Jan 3;4(2):148–59. doi: 10.1021/acsphyschemau.3c00039 (PMC10979484; doi:10.1021/acsphyschemau.3c00039)
Supplement: Supplementary file 1 — pg3c00039_si_001.pdf [file pg3c00039_si_001.pdf]

Supplemental Information

Magnetoelectrocatalysis: Evidence from the Hydrogen Evolution Reaction

Krysti L. Knoche Gupta, Heung Chan Lee, and Johna Leddy*

University of Iowa
Department of Chemistry
Iowa City, IA 52240 USA
johna-leddy@uiowa.edu

Abstract

Hydrogen evolution reaction (HER) rates are higher where magnetic gradients are established at the electrode surface. In comparison of literature data for metals with comparable work functions, we note 1000 $\times$ higher rates for paramagnetic metals than diamagnetic metals. With unpaired electron spins, paramagnetic and ferromagnetic metals establish interfacial magnetic gradients. At diamagnetic electrodes, gradients are induced by addition of magnetized microparticles. Onset of hydrogen evolution for magnetized γ-Fe_2O_3 microparticles in Nafion on diamagnetic glassy carbon electrodes is lower by 190 mV (-18 kJ mol^{-1}) relative to demagnetized microparticles. Chemically the same as demagnetized particles, the physical distinction of magnetic field and gradient at magnetized microparticles increases electron transfer rate. For magnetized Fe_3O_4 microparticles, the onset is lower by 280 mV (-27 kJ mol^{-1}). Paramagnetic platinum electrodes are unaffected by addition of magnetized microparticles. Magnetoelectrocatalysis is established by magnetic gradients.

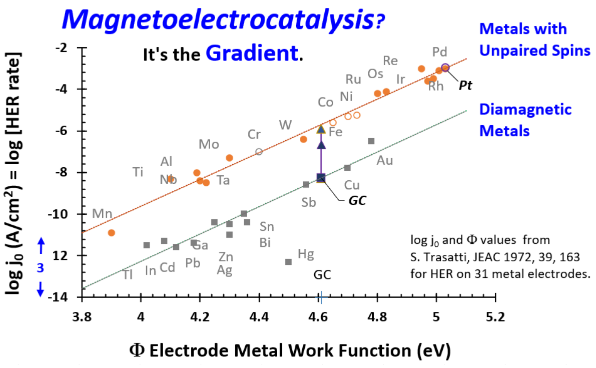

TOC Graphical Abstract

SI.1 Exchange Current Densities j_0 and Work Functions Φ as Tabulated by Trasatti [1] SI.3
SI.1.1 Other Compilations of $\log j_0$ Values . . . SI.4
SI.1.2 For $\Phi = 4.3eV$: Correlation of $\log j_0$ with Molar Magnetic Susceptibility χ_M . . . SI.4
SI.1.3 Synopsis of Section 1.1, Figure 1, and Magnetic Impact Noted in Literature Data . . . SI.5

SI.2 Derivations SI.5
SI.2.1 Development of Potential Dependent Rate Expressions for k^0 and j_0 . . . SI.5
Mechanism for HER . . . SI.5
Eyring Equation . . . SI.5
Measurements and Electrochemical Rate Parameters . . . SI.6
Exchange Current Density j_0 . . . SI.6
Analysis of Voltammetric Data . . . SI.7
SI.2.2 Relationship between ΔE and j_0^{mag}/j_0^{Naf} . . . SI.7
For Figure 2 . . . SI.7
For Figure 1 . . . SI.7
For Figure 2 and GC Data . . . SI.7

SI.3 Methods Additional Details SI.7
SI.3.1 Materials and Characterization . . . SI.8
SI.3.1.1 Nafion . . . SI.8
Calculation of c^* in Nafion cast films . . . SI.8
Nafion Nanostructure . . . SI.8
Nafion Film Modified Electrodes as Control . . . SI.8
SI.3.1.2 Magnetic Microparticles . . . SI.8
Distinctions in CX Fe_2O_3 Microparticles and In House 5 μm Fe_3O_4 Microparticles . . . SI.9
Chemical and Electrochemical Inertness . . . SI.9
Characterization by Magnetic Susceptibility, χ . . . SI.9
In-house Magnetite (Fe_3O_4) Siloxane Coating Procedure [5] . . . SI.9
Protocol to Demagnetize Magnetic Microparticles . . . SI.10
SI.3.1.3 Casting Films and Composites . . . SI.10
SI.3.2 Details of LSV Measurements . . . SI.11
SI.3.3 External Magnet NdFeB . . . SI.11
SI.3.3.1 Electrochemistry of Ru(bpy)$_3^{2+}$ in Nafion . . . SI.11
Use of Ru(bpy)$_3^{2+}$. . . . SI.11

SI.4 Results For Magnetically Modified Diamagnetic GC Electrodes SI.12
LSV Data for Magnetized and Demagnetized Composites of CX γ-Fe_2O_3 Microparticles and Nafion Films . . . SI.13
LSV Data for Magnetized Fe_3O_4 Composites and Nafion Films . . . SI.13
Comparison of Nafion Films and Magnetic Composites on GC . . . SI.13
Comparison of Magnetized Composites and Demagnetized Composites on GC . . . SI.15
Increase in rate on GC with magnetic content . . . SI.16
SI.4.1 Glassy Carbon Data on Figure 1 . . . SI.17
SI.4.2 Synopsis of Results for Diamagnetic Electrodes Modified with Nafion, Magnetized Composites, and Demagnetized Composites . . . SI.17
SI.4.2.1 Magnetic Modification of *Diamagnetic* Metal Electrodes *without Inherent Spin* Increases HER Rate . . . SI.17
Magnetized Composites and Nafion Films on Diamagnetic Electrodes . . . SI.18
Magnetized Composites and Demagnetized Composites on Diamagnetic Electrodes . . . SI.18

SI.5 Results for Paramagnetic Platinum **SI.18**
SI.5.1 LSV on Pt SI.18
SI.5.1.1 $\gamma-Fe_2O_3$ Composites under N_2 Blanket in 0.10 M HNO_3 [4]: SI.19
SI.5.1.2 Fe_3O_4 Composites under H_2 Blanket in 1.0 M HNO_3 [5] SI.19
SI.5.1.3 Electrodes Blocked by Microparticles and Electrochemical Surface Area (ECSA) SI.22
SI.5.2 Open Circuit Potential under H_2 Blanket on Pt [5] SI.22
SI.5.3 Because Pt Response Is Not Altered by Magnetized Composites Compared to Nafion SI.23
SI.5.3.1 Thermodynamics SI.23
SI.5.3.2 Not Mediated SI.24
SI.5.3.3 Not Magnetically Driven Mass Transport Enhancement SI.24
SI.5.4 Synopsis of No Magnetic Impact on HER Rate on Paramagnetic Pt SI.24

SI.6 Results for an External Applied Uniform Magnetic Field **SI.24**
SI.6.1 Unmodified Pt Disk and Nafion Film Modified Pt Disk SI.25
SI.6.2 Pt Disks Modified with Nafion Films, Magnetized C1 Microparticles Composites, and Demagnetized C1 Microparticles Composites SI.25
SI.6.3 Quantitative Results for External, Uniform Field Applied with NdFeB Ring Magnet SI.26
SI.6.4 Gradient and the Electrode Electrolyte Interface SI.27
Paramagnetic Atom SI.27
Microparticles SI.27
Uniform Field SI.28
Note on Generation of a Gradient Field with Standard Laboratory Magnets SI.28

SI.1 Exchange Current Densities j_0 and Work Functions Φ as Tabulated by Trasatti [1]

Trasatti compiled best measured exchange current densities j_0 (A/cm^2) and work functions Φ (eV) for the hydrogen evolution reaction (HER) at 31 metal electrodes that are drawn from literature data until the early 1970s. The data are for polycrystalline metals measured in aqueous electrolyte at pH 0 and room temperature. Acids are typically H_2SO_4 but a few are reported in $HClO_4$ and HCl. Trasatti's data compilation is shown in Table SI.1. Data from Trasatti's work on measured current densities (j_0) measured near equilibrium potentials and the electrochemical work functions Φ are tabulated in Table SI.1. Where $\log j_0$ is reported measured positive and negative of the potential of zero charge, values negative of the PZC are tabulated. Values are taken from Reference [1].

**Metal**	$\log \mathbf{j}_0$ **(A/cm^2)**	$\mathbf{\Phi}$ (eV)
Au	-6.5	4.78
Cu	-7.8	4.70
Sb	-8.6	4.56
Sn	-10	4.35
Bi	-10.4	4.25
Ga	-10.4	4.30
Zn	-10.5	4.30
Ag	-11	4.30
In	-11.3	4.08
Pb	-11.4	4.18
Tl	-11.5	4.02
Cd	-11.6	4.12
Hg	-12.3	4.50
Pt	-3	5.03
Re	-3	4.95
Pd	-3.1	5.01
Rh	-3.5	4.99
Ir	-3.6	4.97
Os	-4.1	4.83
Ru	-4.2	4.80
W	-6.4	4.55
Mo	-7.3	4.30
Al	-8	4.19
Ti	-8.3	4.10
Nb	-8.4	4.20
Ta	-8.5	4.22
Mn	-10.9	3.90
Ni	-5.25	4.73
Co	-5.3	4.70
Fe	-5.6	4.65
Cr	-7	4.40

Table SI.1: Trasatti's Compilation of $log j_0$ and Electrochemical Work Function Φ. Data are shown segregated into diamagnetic metals, paramagnetic metals, and ferromagnetic metals with Cr antiferromagnetic.

Trasatti's work built on work by Bockris [73], Parsons [74], and Conway and Bockris [75] first noted the correlation of $\log j_0$ with Φ for a limited number of electrode metals. Work functions measure the energy to remove an electron from the surface of a material to a point outside the material, typically to vacuum. Trasatti used the work function as the estimate of the energy to move an electron from the surface of the electrode to

a redox species in solution positioned immediately at the electrode electrolyte interface. This estimate yields the plot in Figure 1 where $\log j_0$ varies linearly with Φ. Two parallel lines are found from the data. For a given Φ, data on the lower line have j_0 values for HER 1000 fold lower than j_0 values on the upper line. Trasatti labeled the metals on the lower rate line as *sp* metals and metals on the upper rate line as *d* metals. It is noted that Al is included with the *d* metals.

Here, we identify all the metals on the lower rate line as diamagnetic with no unpaired electrons, no net spin, and no inherent magnetic field and magnetic gradients. All metals on the higher rate line have unpaired electrons, net spin, and the ability to establish magnetic fields and magnetic gradients. Al is paramagnetic.

SI.1.1 Other Compilations of $\log j_0$ Values

Trasatti's $\log j_0$ values for polycrystalline metals remain largely unchanged over time. Two other compilations of $\log j_0$ values for HER are considered. For polycrystalline electrodes, Nørskov et al. [76] summarizes data drawn from [1] and Bockris and Reddy [77] as single values of $\log j_0$. Petrii and Tsirlina [78] report ranges for $\log j_0$. Most of Trasatti's $\log j_0$ values are within ± 0.2 of the values cited in reference [76] and fall within the ranges in reference [78]. The exceptions are Nb, W, and Au in Nørskov (Bockris and Reddy) and Mn, Cu, Pb, and Tl in Petrii and Tsirlina. Only Nb, Au, and Pb differed by more than ± 1 and all differed by less than ± 1.8. More details about the compiled data and impact on regression statistics are provided in SI.2 and SI.5, respectively.

The regression statistics based on Trasatti's data are negligibly impacted by $\log j_0$ values in References [76–78]. The large number of *d* and *sp* metals, the limited number of values with more than ± 0.2 differences in $\log j_0$, the small magnitude of the differences (≤ 1.7), and a single significant figure in Trasatti's data all contribute to statistically appropriate modeling based on data in Reference [1] that suffices for the current discussion.

SI.1.2 For $\Phi = 4.3$ eV: Correlation $-\log j_0$ with Molar Magnetic Susceptibility χ_M

For the three metals with the same electrochemical work function, Φ, variation of $\log j_0$ with the molar magnetic susceptibility χ_M (cm^3/mol) of the metal electrode is linear as shown in Figure SI.1. Ag and Zn are diamagnetic and Mo is paramagnetic, with j_0 10^3 to 10^4 higher on Mo. For a given work function, the rate of reaction is shown to increase with magnetic properties of the metal electrode. [4] From Table SI.1, only this single case of three metals with the same Φ is available. From Figure 1, where paramagnetic metals and diamagnetic metals have the same Φ (4.18 $\pm$ 0.005: Al, Pb; 4.55 $\pm$ 0.005 W, Sb; 4.11 $\pm$ 0.01 Ti, Cd) the electrodes with inherent net spin support about 10^3 higher exchange current densities.

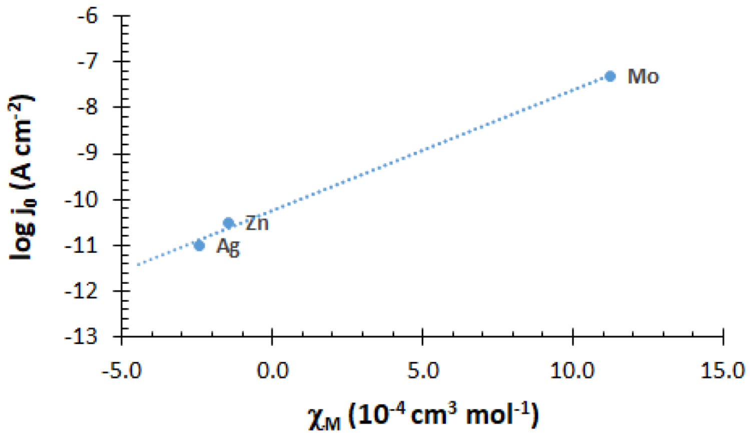

Figure SI.1: For three metals with the same electrochemical work function $\Phi = 4.3$ eV (Ag, Zn, and Mo), $\log j_0$(A/cm^2) is linear with the molar magnetic succeptibility, χ_M.(10^{10} cm^3/mol) of the metals. $\log j_0$(A/cm^2) $=$ $(2.6_3 \pm 0.1_5) \times 10^{-4} \chi_M (cm^3 mol^{-1}) - (10.2 \pm 0.1)$, for $R^2 = 0.997$. Zn and Ag are diamagnetic and Mo is paramagnetic.

SI.1.3 Synopsis of Section 1.1, Figure 1, and Magnetic Impact Noted in Literature Data

From Figure 1, the inherent spin of the metal electrode strongly impacts the HER rate. For a given Φ, metal electrodes with inherent electron spin support HER rates j_0^{spin} that are a thousand fold higher than diamagnetic electrodes without unpaired electrons. This corresponds to an energetic advantage $\Delta G = -17$ kJ/mol and a decrease in overpotential that shifts the onset potential for hydrogen evolution by about +180 mV.

Rates of HER electrocatalysis are higher on paramagnetic and ferromagnetic metal electrodes than on diamagnetic electrodes (Section 1.1, Figure 1). Paramagnetic and ferromagnetic metals have unpaired electron spins; in diamagnetic metals, all spins are paired. For the 31 electrodes in Figure 1, two parallel data sets are shown as linear variation of $\log j_0$ with Φ. For a given work function Φ, electrodes with inherent electron spin support exchange current densities a thousandfold higher than the electrodes with no inherent electron spin; $j^{spin} \approx 10^3 j^{diam}$. Here with no exceptions, the two data sets are starkly discriminated by electrode magnetic properties. Further, the magnetic impact on $\log j_0$ is binary, either on or off, set by whether the electrode metal has inherent net spin or not. For a given Φ in Figure 1, the energy difference is estimated as -17 kJ/mol; for a given $\log j_0$, the difference in Φ is -0.48 eV or -46 kJ/mol. Supplemental evidence of a magnetic effect on the HER rate is shown in Figure SI.1 where for fixed $\Phi = 4.3$ eV (Ag, Zn, Mo) $\log j_0$ varies linearly with the magnetic susceptibility of the metal. A substantial magnetic impact on rates of HER electrocatalysis is identified.

No pattern of $\log j_0$ with the magnetic properties of the metals in Table SI.1 is found other than segregation into diamagnetic metals and metals with inherent spin. For the metals with inherent spin, no pattern is observed with the number of unpaired electrons. From Table SI.1, $\log j_0$ for $\Phi = 4.30$ is linearly correlated with the molar magnetic susceptibility of the metals (Figure SI.1). For GC, $\Phi = 4.61$ eV [53] and magnetized Fe_3O_4 composites yield similar ΔE.

SI.2 Derivations

Derivations are for single electron transfer $(n = 1)$ reactions in $A + ne \rightleftharpoons B$ at formal potential $E^{0\prime}$. Formal potential embeds experimental conditions that may differ from the standard conditions required at standard potential E^0. Current perspectives on electron transfer events conclude that electron transfers can be sequential with the first electron transfer slow and the second electron transfer fast for an apparent two electron transfer $(n = 2)$. But, the first and independent electron transfer event is a single, rate determining electron transfer.

SI.2.1 Development of Potential Dependent Rate Expressions for k^0 and j_0

Mechanism for HER HER is not simple kinetically. The most common (VHT) mechanism [8] names steps for Volmer, Heyrovsky, and Tafel.

$$\mathrm{H}^+_{\mathrm{soln}} \rightleftharpoons \mathrm{H}^+_{\mathrm{ads}} \quad \text{(Step 1)}$$
$$\mathrm{H}^+_{\mathrm{ads}} + e \rightleftharpoons \mathrm{H}^\bullet_{\mathrm{ads}} \quad \text{(Step 2)}$$
$$2\mathrm{H}^\bullet_{\mathrm{ads}} \rightleftharpoons \mathrm{H}_{2,\mathrm{ads}} \quad \text{(Step 3)}$$
$$\mathrm{H}_{2,\mathrm{ads}} \rightleftharpoons \mathrm{H}_{2,\mathrm{soln}} \quad \text{(Step 4)}$$

Proton H^+ adsorbs to the electrode and undergoes electron transfer to form adsorbed hydrogen radical $H^\bullet_{ads}$ (Volmer), with Steps 1 and 2 in either sequence or concert. In Step 3, two $H^\bullet_{ads}$ combine to form adsorbed dihydrogen $H_{2,ads}$ from either a single metal atom (Heyrovsky) or adjacent metal sites (Tafel). The Volmer step is often rate determining.

Eyring Equation The Eyring equation expresses a generic rate constant k as exponentially dependent on the Gibbs free energy of activation $\Delta G^\ddagger$ with pre-exponential factor A'. Along the reaction coordinate of transition state theory, $\Delta G^\ddagger$ is the maximum energy difference between the reactants and transition state. $\ln k$ evaluated with temperature as T^{-1} yields $\Delta G^\ddagger$ and A'.

$$k = A' \exp\left[-\frac{\Delta G^\ddagger}{RT}\right] \quad \text{(SI.1)}$$

Analysis by the Eyring equation typically measures rate as a function of temperature to determine A' and $\Delta G^{\ddagger}$. The Eyring equation alone does not identify the rate determining step(s).

Electrochemical rate constants are also expressed in Eyring form. To determine rate constants, current density $j\left(E\right)$ (A/cm^2) is measured as a function of potential applied to the electrode, E (V). E is applied relative to the thermodynamic characteristic for the reaction, the standard potential E^0, or under experimental conditions specified other than standard conditions, relative to the formal potential $E^{0'}$.

Measurements and Electrochemical Rate Parameters For a simple electron transfer at the electrode electrolyte interface, potential dependent rate constants $k_f\left(E\right)$ and $k_b\left(E\right)$ are assigned.

$$O + e \underset{k_b(E)}{\overset{k_f(E)}{\rightleftharpoons}} R \tag{SI.2}$$

Concentrations of O and R immediately at the electrode surface $C_O\left(E\right)$ and $C_R\left(E\right)$ also vary with E. Electron flux at the electrode solution interface $j\left(E\right)/F$ (mole cm^{-2}s^{-1}) is characterized phenomenologically by the Butler Volmer equation, which applies where there are no mass transport limitations and no chemical steps. [9] For $f = F/RT$,

$$\frac{j\left(E\right)}{F} = k_f C_O\left(E\right) - k_b C_R\left(E\right) \tag{SI.3}$$

$$= k^0 \left[\begin{array}{c} C_O(E)\exp\left[-\alpha f\left(E - E^{0'}\right)\right] - \\ C_R(E)\exp\left[\left(1-\alpha\right)f\left(E - E^{0'}\right)\right] \end{array}\right] \tag{SI.4}$$

Rate constants $k_f\left(E\right) = k^0 \exp\left[-\alpha f\left(E - E^{0'}\right)\right]$ and $k_b\left(E\right) = k^0 \exp\left[\left(1-\alpha\right)f\left(E - E^{0'}\right)\right]$ are potential dependent. The standard heterogeneous rate constant k^0 (cm/s) is measured at $E = E^{0'}$. Transfer coefficient α describes partition of the potential dependent component of the activation free energy $\Delta G^{\ddagger}$ between $O + e$ and R. Specifically, $\alpha = F^{-1}\partial\Delta G^{\ddagger}/\partial E$, where $0 \le \alpha \le 1$ with α typically ≈ 0.5. Heterogeneous electron transfer is characterized by k^0 and α.

For total concentration of reactant O and product R present in the electrolyte at concentration c^* (mol cm^{-3}), surface concentrations $C_O\left(E\right) + C_R\left(E\right) = c^*$ as $E - E^{0\prime}$ varies. For only O in the electrolyte, measurements made where $E - E^{0\prime} \gtrsim 0$ and changes in $E - E^{0\prime}$ are not extreme, $C_O\left(E\right)$ is little perturbed ($C_O\left(E\right) \to c^*$) and equation SI.4 is simplified.

$$\frac{j\left(E\right)}{Fc^*} = k^0 \exp\left[-\alpha f\left(E - E^{0'}\right)\right] \tag{SI.5}$$

Equation SI.5 is in Eyring form where k^0 is potential independent A' and the potential dependent component of $\Delta G^{\ddagger}$ equals $\alpha F\left(E - E^{0'}\right)$. Electrochemical rate measurements avoid temperature studies as potentiostatic control of $E - E^{0'}$ titrates $\Delta G^{\ddagger}$.

Exchange Current Density j_0 $j\left(E\right)$ is often more readily measured relative to the equilibrium potential E_{eq} rather than $E^{0\prime}$. E_{eq} is measured at the open circuit potential where there is no current flow. Concentrations and $E^{0\prime}$ are incorporated in E_{eq} but not measured explicitly. Analogous to equation SI.5, $j\left(E\right)$ is measured against overpotential $E - E_{eq}$. Rate is characterized by α and the exchange current density j_0. At $E = E_{eq}$, $j\left(E_{eq}\right) = j_0$.

$$j(E) = j_0 \exp\left[-\alpha f\left(E - E_{eq}\right)\right] \tag{SI.6}$$

j_0 is measured at $E = E_{eq}$ whereas k^0 is measured at $E = E^{0\prime}$. Equating electrochemical flux $j\left(E\right)/F$ of equations SI.5 and SI.6, k^0 and j_0 characterize electrocatalytic rate.

$$j_0 = Fc^* k^0 \exp\left[-\alpha f\left(E_{eq} - E^{0\prime}\right)\right] \tag{SI.7}$$

Analysis of Voltammetric Data In voltammetric measurements, E controls $\Delta G^{\ddagger}$ and rate is measured as $j(E)$. Rates are compared as either $j(E)$ at fixed E or E at fixed $j(E)$. Here, measurements are made at a fixed current density to find differences in potential or overpotential that determine relative rate constants and energies. To compare two rates with common E_{eq} and α, E values measured at fixed $j(E)$ yield potential difference ΔE. From ΔE, rate constants and energy are found. Energy difference $\Delta G = -F\Delta E$ (J/mol). From equation SI.6, $\Delta \ln j_0 = -\alpha f \Delta E$, where $\Delta \ln j_0$ yields the ratio of j_0 values (SI.2.2). At fixed c^*, j_0 ratios equal k^0 ratios (equation 4). The relationship between ΔE and rate constants an energy are defined as follows.

SI.2.2 Relationship between ΔE and j_0^{mag}/j_0^{Naf}

To compare the rates at a magnetized composite and a Nafion film, $\Delta E = E_{mag} - E_{Naf}$ is determined. The potentials E_{mag} and E_{Naf} are measured at a fixed current density $j(E)$ for the magnetized composite and the Nafion film. Voltammograms are measured at a common c^*, scan rate, and temperature.

From equation 3 at a fixed potential $j(E)$,

$$j(E) = j_0^{mag} \exp\left[-\alpha f \left(E_{mag} - E_{eq}\right)\right] = j_0^{Naf} \exp\left[-\alpha f \left(E_{Naf} - E_{eq}\right)\right] \tag{SI.8}$$

For common α,

$$\log \frac{j_0^{mag}}{j_0^{Naf}} = -\frac{\alpha f}{2.303}\Delta E = \log \frac{k_{mag}^0}{k_{Naf}^0} \tag{SI.9}$$

Note that under these conditions, measurement of ΔE yields $\log\left[j_0^{mag}/j_0^{Naf}\right] = \log\left[k_{mag}^0/k_{Naf}^0\right]$. Equation SI.9 is re-expressed as follows

$$\frac{j_0^{mag}}{j_0^{Naf}} = 10^{-\frac{\alpha f}{2.303}\Delta E} = \frac{k_{mag}^0}{k_{Naf}^0} \tag{SI.10}$$

At 25 °C, $\alpha f/2.303 = \alpha\left(0.05916\ V\right)^{-1}$; for $\alpha = 0.5$, ΔE of $+118$ mV corresponds to $j_0^{mag} \approx 10 j_0^{Naf}$.

For Figure 2 For all diamagnetic electrodes, H_2 evolves more easily at electrodes modified with magnetized composites than diamagnetic Nafion films (Figure 2 and Table 1); $\Delta E > 0$ and $j_0^{mag}/j_0^{Naf} > 1$.

For Figure 1 From an Eyring perspective on Figure 1, energy of the magnetic interactions is estimated as $\Delta \log j_0 = \log\left(j_0^{spin}/j_0^{diam}\right) = \log\left(k_{spin}^0/k_{diam}^0\right)$ that captures impacts of electrode spin on electrocatalytic rate. Express j_0^{spin} and j_0^{diam} in Eyring form ($k = A' \exp[-\Delta G^{\ddagger}/(RT)]$) and allow impacts of spin ascribed to free energy of activation terms $\Delta G_{spin}^{\ddagger}$ and $\Delta G_{diam}^{\ddagger}$ with comparable pre-exponential factors ($A'_{spin} \approx A'_{diam}$). Then, $\Delta \log j_0 = -(2.303RT)^{-1}\left(\Delta G_{spin}^{\ddagger} - \Delta G_{diam}^{\ddagger}\right)$.

For Figure 2 and GC Data In Figure 2 GC data are added as up arrows to show the increase in rate with magnetized composites as compared to diamagnetic Nafion films. ΔE determines the change in $\log j_0$ on magnetic modification of diamagnetic electrodes. The magnitude of the shift in $\log j_0$ is found from equation SI.9.

$$\log j_0^{mag} = \log j_0^{Naf} - \frac{\alpha f}{2.303}\Delta E \tag{SI.11}$$

Equivalently for the standard heterogeneous rate,

$$\log k_{mag}^0 = \log k_{Naf}^0 - \frac{\alpha f}{2.303}\Delta E \tag{SI.12}$$

Measured ΔE finds $\log j_0^{mag}$ calculated from equation SI.11, given α.

SI.3 Methods - Additional Details

Additional experimental details and minor variants are provided.

SI.3.1 Materials and Characterization

Properties and characterization of various materials are described.

SI.3.1.1 Nafion

Nafion® (DuPont) is a perfluorinated cation exchange polymer that maintains high flux of cations (proton) and sets the concentration of cations at the electrode surface. Nafion has sufficient mechanical strength to hold the magnetized microparticles at the electrode surface. Nafion suspension (5 w/v %) in aliphatic alcohols and water has a nominal equivalent weight of 1100 g of polymer per mole of sulfonate.

The density of Nafion has been reported. [79, 80] For proton exchanged Nafion, the density is 1.8 g/cm^3.

Calculation of c^* in Nafion cast films The concentration of proton in Nafion c^* (mol/cm^3) is calculated across the bulk film as

$$\frac{1.8 \text{ g/cm}^3}{1100 \text{ g/mol}} = 1.6_4 \text{ mmol/cm}^3 = 1.6 \text{ M} \tag{SI.13}$$

Nafion Nanostructure Nafion forms a nanostructured, biphasic matrix of aquated (density 1.00 g/cm^3) and fluorocarbon domains (density 2.00 g/cm^3). From the density, the water fraction of Nafion is 0.43. In the water filled domains, the proton concentration is 3.8 M. [55, 81]

Hydrated Nafion is forms a heterogeneous nanostructure because the fluorocarbon and aquated phases segregate. [13, 54, 55] Domains in Nafion are on submicrometer lengths. There are no bulk fluid domains in Nafion.

Nafion Film Modified Electrodes as Control In Figure 2a at an unmodified electrode (tan -···), H_2 evolves at potentials negative of Nafion (red dots) because Nafion concentrates proton ($c^*_{Naf} \approx$ 1.6 M) [55] as compared to 0.1 M HNO_3 electrolyte that yields higher current densities at lower overpotentials for Nafion films compared to unmodified electrodes.

To maintain the same proton concentration, composites are compared to Nafion films rather than unmodified electrodes.

SI.3.1.2 Magnetic Microparticles

The commercial and in-house made magnetic microparticles used here are ferrimagnets that respond to an external magnetic field, can be magnetized with a stronger rare earth magnet (NdFeB), and can be demagnetized on prolonged agitation. Agitation should be gentle to avoid fracturing the brittle ferrimagnets. Compared to maghemite ($\gamma{-}Fe_2O_3$, orange), magnetite (Fe_3O_4, black) has higher iron fraction and slightly higher density and supports slightly higher saturation magnetization. On magnetization of comparably sized ferrimagnets, the field about Fe_3O_4 magnets is stronger than about Fe_2O_3.

Some properties of magnetite and maghemite are shown in Table SI.2. [82]

Ferrimagnets retain a magnetic field provided they are of sufficient size to maintain spin alignment after magnetization. If the particle size is too small to maintain alignment, then the particle behaves like a paramagnetic material. That is, spin is aligned in the external field but loses magnetization (alignment) once removed from the external field. A particle is labeled superparamagnetic if particle composition is a ferrimagnetic compound but domain size is too small to maintain alignment outside the field. From Table SI.2, the 1 μm $\gamma{-}Fe_2O_3$ and 5 μm Fe_3O_4 used here are sufficiently large that the particles behave as permanent ferrimagnets. These particles are not superparamagnetic.

Loading, particle size, and magnetic material impact the magnetic field and gradient at the electrode surface. A balance of magnetization M, particle radius r, and distance from the surface of the particle Δr will determine whether sufficient gradient is established at the electrode electrolyte interface. Further brief discussion is in SI.6.4. At higher loadings, microparticles can block assess to the electrode surface area. Coatings on microparticles ensure chemical and electrochemical inertness, but coatings should be thin to establish a field gradient outside the particle coating.

	Magnetite Fe_3O_4	Maghemite $\gamma-Fe_2O_3$	Units and Notes
Molecular Weight	231.553	159.687	g mol^{-1}
Density ρ	5.18	4.90	g cm^{-3}
Saturation magnetization	90-92	70-80	A m^{2}kg^{-1} at room temperature
Neel temperature T_N	575-585	600	°C
$T > T_N$ ferrimagnetic to paramagnetic transition			
size superparamagnetic to ferrimagnetic transition (single domain)	≈25	≈32	nm [83, 84]

Table SI.2: Some Bulk Properties of Magnetite (Fe_3O_4) and Maghemite ($\gamma - Fe_2O_3$). [82]

Distinctions in CX Fe_2O_3 Microparticles and In House 5 μm Fe_3O_4 Microparticles The bulk properties of Fe_3O_4 and γ-Fe_2O_3 do not differ substantially (Table SI.2). Composites all contain 15 to 20 % particles and thicknesses of the composites and Nafion films are all 3 to 5 μm. The difference may arise from properties of the microparticles themselves. Magnetic fields and gradients vary with magnet geometry and the number of unpaired spins in the magnet volume. The C1 particles are spherical formed by capturing small γ-Fe_2O_3 microparticles in a siloxane matrix. The particles are spherical with a density of about 2 g cm^{-3}. The volume of a 1 μm C1 particle is ~5 × 10^{-13} cm^3 with ~2 × 10^8 C1 particles and 3 μmol of total iron in a 5 μm thick composite. The in house Fe_3O_4 microparticles are a magnetite core shrouded in siloxane. The ball milled particles are about 5 μm in diameter and not spherical. If approximated as spherical, a 5 μm Fe_3O_4 particle volume is ~7 × 10^{-11} cm^3 with ~2 × 10^6 particles and 7 μmol of total iron in a 5 μm thick composite. Density of Fe_3O_4 microparticles is higher than C1 particles and Fe_3O_4 particles are less well suspended in Nafion. Both Fe_3O_4 and C1 microparticles establish magnetic gradients at electrode surfaces to impact rates of HER on diamagnetic electrodes, but sources of the differences are not quantified.

Chemical and Electrochemical Inertness Both commercial and in-house coated particles are chemically and electrochemically inert. If the particles are not inert, iron leached from the magnetic particles might be suspected to mediate electron transfer reactions (SI.5.3.2). Several methods verify that the particles are chemically and electrochemically inert. The particles are soaked for extended periods (days to months) in concentrated HNO_3 and no iron is found in the supernatant colorimetrically with phenanthroline. No iron signal is ever observed in the voltammetry at electrodes modified with microparticle composites. Composites formed with demagnetized particles do not increase in current as they would if iron mediated electron transfer processes.

Characterization by Magnetic Susceptibility, χ Magnetic content of C1, C3, and C8 SiMag microparticles in Nafion composites are measured as volume magnetic susceptibility χ_V (cm^3 mol^{-1}) in a Guoy balance. [4] Values of χ_v for the 15 % (vol/vol) composites are shown in Table SI.3. The microparticle composites are attracted to the magnetic field of the Guoy balance, which confirms the particles have unpaired spins. For the commercial SiMag CX particles, χ_v decreases as the length of the siloxane chain increases. The measured χ_v for the microparticle composites tracks the net aligned, unpaired spins introduced to the electrode surface in magnetized composites.

Volume susceptibility is also measured for Nafion without magnetic particles. For Nafion, masses changed slightly on application of the magnetic field in the Guoy balance and Nafion is slightly repelled by the field. The response marks Nafion as diamagnetic. As a perfluorocarbon sulfonate polymer, Nafion is anticipated to be diamagnetic. The small measured value for Nafion is at the limit of sensitivity and serves only as an estimate of χ_v for Nafion films.

For methyl-siloxane (SiMag-C1), propyl-siloxane (SiMag-C3), and octyl-siloxane (SiMag-C8) coated particles, volumetric magnetic susceptibility of microparticles suspended in Nafion was determined by Guoy balance to decrease with chain length. [4] Relative χ_v values reported in the Table are values normalized by the χ_v for C1 composites. Also shown in the Table are $\Delta E = E_{mag} - E_{Naf}$ measured at 0.4 mA cm^{-2} for glassy carbon electrodes modified with CX particles.

In-house Magnetite (Fe_3O_4) Siloxane Coating Procedure [5] The inhouse coating procedure for the ferrimagnetic magnetite particles is as follows and follows procedures previously outlined [12, 64, 85]. Magnetite

	χ_v (10^{-6} cgs)	χ_v (μB)	rel. χ_v	$\Delta E = E_{mag} - E_{Naf}$ (V)
SiMag				
C1	16.1 ± 0.8	$2.4_8 \pm 0.1_2$	1.00	0.191 ± 0.019
C3	10.2 ± 0.8	$1.8_5 \pm 0.1_4$	0.63_5	0.123 ± 0.022
C8	$8.7_8 \pm 0.1_2$	1.44 ± 0.02	0.54_7	0.111 ± 0.028
Nafion	-0.1		-0.006	0

Table SI.3: Measured χ_v and ΔE for composites of 15 % (vol/vol) C1, C3, and C8 Magnetized Microparticles in Nafion on GC. Relative χ_v values are normalized by χ_v for C1 particles. ΔE is measured at 0.4 mA cm^{-2} at CX modified glassy carbon (GC) electrodes. Data for Nafion films are also noted.

(Fe_3O_4, Aldrich) particles (Aldrich, $\lesssim 5$ μm) are ball milled in hexane for 20 minutes. Magnetite particles (1 g), toluene (10 mL) and (3-aminopropyl)trimethoxysilane (10 mL; Alfa Aesar) in a 20 mL screw cap vial are mixed by slow rotation of the vial for 4 hours. Supernatant is decanted and particles are washed with toluene (3 $\times$ 20 mL). The particles are dried overnight in a vacuum oven at 70 ^{o}C. Ethylene glycol diglycidyl ether (10 mL) and deionized water (10 mL) are added into the vial of dried particles and agitated by slow rotation ($\sim$30 rpm) for 4 hours. Particles are rinsed with deionized water (3 $\times$ 20 mL), dried overnight in a vacuum oven at 70 ^{o}C, and stored in a capped vial. Thickness of the encapsulation is controlled by quantities of added reagents. As prepared here, the siloxane encapsulated magnetic particles are $\lesssim$ 5 μm in diameter. Sizes of magnetic particles are little changed by the siloxane coating of 1 to 3 nm as determined by thermal gravimetric analysis (TGA) [12]. Properties, coating thickness, and particle size vary from batch to batch and coating thickness and size of the particle can vary by batch. However, scanning electron microscope (SEM) images find typical diameters $\sim$ 4 - 5 μm.

SAFETY WARNING: In a slight variant of the siloxane coating procedure, approximately 1 cc of coated Fe_3O_4 particles were dried in an oven overnight at 40 °C. While collecting the particles from the scintillation vial, the particles detonated. Use appropriate precautions when working with freshly coated Fe_3O_4 microparticles.

Protocol to Demagnetize Magnetic Microparticles Magnetic microparticles can be demagnetized by heat and agitation. Because magnetic materials are typically brittle, gently agitation is needed to avoid fracturing the particles. To demagnetize the microparticles, several milliliters the suspension of microparticles in Nafion are placed in capped vial. The vial is rotated slowly for several hours with a drill at speeds controlled with a voltage regulator.

SI.3.1.3 Casting Films and Composites

To cast a Nafion film, Nafion suspension (Aldrich, 5 % (w/v) in water aliphatic alcohol mixture, 1100 EQWT) is pipetted on to the electrode surface. The volume pipetted is calculated from the electrode area and final film thickness and density. [80] For example, a 7 μm thick Nafion film is formed in acid electrolyte on a 0.4525 cm^2 electrode from 5.0 μl of suspension.

For γ-Fe_2O_3 CX particles, microparticles are mixed with Nafion suspension in a 1:20 volumetric ratio in a microcentrifuge tube. Suspension is vortexed for 5 seconds immediately before casting. This ratio yields a 6 % (w/w) loading of microparticles in the dry Nafion film. The micropipetted volume yields a composite of thickness 7 μm in acid solution with 15 % (v/v) microparticles. For demagnetized composites, the microparticles are demagnetized before casting. For magnetized composites, the electrode is centered in a NdFeB ring magnet (o.d. = 7.6 cm, i.d. = 3.8 cm) as casting solvents evaporate. [4]

Casting solvents are allowed to evaporate for $\geq$ 24 hours before electrochemical measurements.

Fe_3O_4 composites are similarly formed. Films and composites are 6 μm thick with either 15 % or 20 % (v/v) microparticles. [5]

All composites and films are evaluated in acidic aqueous electrolyte.

SI.3.2 Details of LSV Measurements

For the electrodes modified with commercial maghemite SiMag CX particles, solutions are degassed and run under a blanket of nitrogen. Electrodes are equilibrated in the electrolyte for 24 hours before measurement and an hour is allowed to re-equilibrate between voltammetric perturbations. A CH Instruments 760B potentiostat is used. All reported scan rates are 50 mV/s.

For electrodes modified with in-house magnetite particles, 1.0 M HNO_3 electrolytes are not degassed for GC and degassed with H_2 for Pt. A CH Instruments 760B potentiostat/galvanostat is used at scan rate of 100 mV/s.

All voltammetry is carried out with a platinum mesh counter electrode and an SCE reference electrode. For all LSV, potential is sweep to negative potentials at a scan rate of 50 or 100 mV/s. Electrolyte is 0.1 or 1.0 M HNO_3.

Except for Fe_3O_4 composites under a hydrogen blanket SI.5.1.2, data are extracted from LSV data without background subtraction.

SI.3.3 External Magnet NdFeB

Cyclic voltammetric studies are undertaken at 0.452 cm^2 Pt disk electrodes unmodified and modified with a 7 μm thick Nafion film. The Nafion film is cast and dried for 24 hours to yield a film 7 μm in acid. [80] Cyclic voltammograms are first undertaken without a ring magnet and then repeated with the neodymium iron boride NdFeB ring magnet (o.d. = 7.6 cm, i.d. = 3.8 cm, 1.3 cm height) encircling the cell with the plane of the Pt disk at the center height of the NdFeB and the electrode shaft centered in the ring magnet, as shown in Figure SI.SI.2. The redox probe tris(2,2'-bipyridine)ruthenium(II) Ru(bpy)$_3^{2+}$ is added as the dichloride salt at a concentration of 0.640 mM in 0.10 M aqueous nitric acid HNO_3. All electrodes equilibrated in the electrolyte for $\gtrsim 24$ hours. All solutions are nitrogen sparged. Three replicates are made for each the Nafion filmed Pt and the unmodified Pt electrodes. Each electrodes is evaluated at 20, 50, 100, and 200 mV/s, each in triplicate scan rates. An hour is allowed between sweeps to re-equilibrate Ru(bpy)$_3^{2+}$ in the Nafion film. Nafion is cation selective and excludes chloride ion. The reference is saturated calomel electrode SCE with a large Pt screen counter electrode. Measurements are made with CH Instruments 760B. NdFeB magnets sustain high fields. Remanence or residual magnetization of NdFeB magnets is about 1 Tesla (10^4 Gauss). In the center of large NdFeB ring magnets, the magnetic field is large and uniform, and gradients in the magnetic field are minimized.

SI.3.3.1 Electrochemistry of Ru(bpy)$_3^{2+}$ in Nafion

Ru(bpy)$_3^{2+}$ concentrates into the hydrated domains in Nafion until the dications are separated by $\approx$ 0.1 nm. [80,81] The diameter of Ru(bpy)$_3^{2+}$ is $\delta = 1.37$ nm. [86] Because the ruthenium complexes are almost touching, the high self exchange rate k_{11} for the Ru(bpy)$_3^{2|3+}$ couple leads to enhanced electron flux in Nafion. The enhanced electron flux manifests as the diffusion coefficient in Nafion D_{Naf}. For Ru(bpy)$_3^{2+}$ concentration in Nafion c^*, $D_{Naf} \approx k_{11}c^*\delta^2/6$. [81, 87, 88] From Marcusian models and appropriate ratios of cation-cation a cation-surface interactions, heterogeneous standard rate k^0 is proportional to k_{11} as $k^0 \sim k_{11}^{1/2}$. [9,89] We have determined that Ru(bpy)$_3^{2+}$ in Nafion demonstrates increased k_{11} and k^0 at Pt and GC electrodes modified with magnetized CX composites. [5, 12, 90]

Knoche et al., determined that flux of Ru(bpy)$_3^{2+}$ in Nafion and aqueous electrolyte are equal where the solution concentration of Ru(bpy)$_3^{2+}$ is 0.64 mM. [91] To eliminate gradients at the film solution interface for these experiments, the concentration of Ru(bpy)$_3$Cl$_2$ is 0.64 mM.

Use of Ru(bpy)$_3^{2+}$. Use of Ru(bpy)$_3^{2+}$ in these experiments offers several advantages. Cyclic voltammetry can be undertaken with Ru(bpy)$_3^{2+}$. There is a cleanly defined peak current i_p in CV and LSV that is easily extracted from voltammetry and quantified. In the CV for Ru(bpy)$_3^{2+}$ on Pt (Figure 3), the peak splitting is 170 mV, which is consistent with heterogeneous electron transfer rates that are not fast. With quasireversible to irreversible electron transfer rates, the uniform external field can impact k^0 as well as k_{11}, although no impact was found (Figures 3 and 4). Prior experience with transition metal complexes in Nafion facilitates experimental design so that diffusion length is less than Nafion thickness and that k^0 and k_{11} are both impacted by magnetic gradients. This provides a good test matrix to see if either electron transfer rate is impacted by the uniform field.

For $Ru(bpy)_3Cl_2$ in solution, impacts of MHD (magnetohydrodynamics) on transport are readily observed. This confirms that field is established at the electrode surface and that the field can impact mass transport. The concentration of $Ru(bpy)_3^{2+}$ in solution is sufficiently low that electron hopping (self exchange) between $Ru(bpy)_3^{2+}$ and $Ru(bpy)_3^{3+}$ does not impact diffusion. The increased currents for $Ru(bpy)_3^{2+}$ and Cl^- observed in Figure 3 top arise solely from MHD effects.

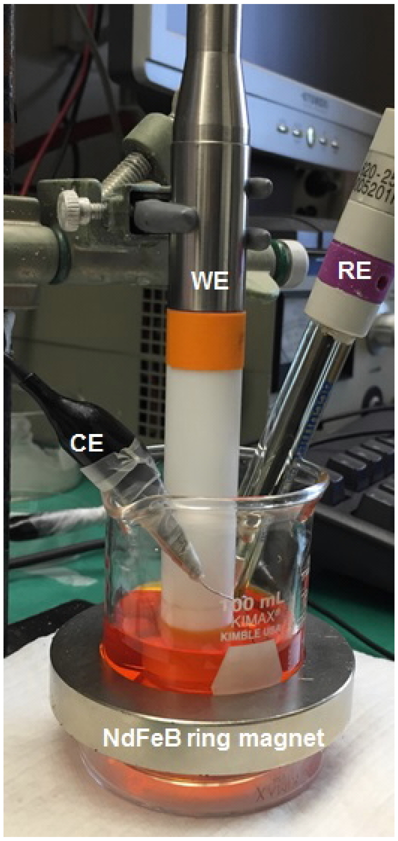

Figure SI.2: Photograph of the three electrode cell in an open beaker. The working electrode (WE) is a Pt disk; the counter electrode is a Pt mesh; and the reference electrode (RE) is a calomel reference (SCE). The solution is $Ru(bpy)_3Cl_2$. The cell is shown with the external rare earth magnet encircling the beaker to establish a uniform magnetic field at disk WE. The NdFeB ring magnet is only placed around the beaker when evaluating uniform fields. In all other experiments, the ring magnet is not used.

SI.4 Results For Magnetically Modified Diamagnetic GC Electrodes

More detailed data and data analysis are presented here for experimental results outlined in the main text in Section 2.

Composites formed of C1 ferrimagnets in Nafion can be magnetized or demagnetized. Demagnetized and magnetized composites have the same chemical composition but a different physical state for magnetized compared to demagnetized. Differences in behavior derived from comparison of magnetized and demagnetized composites do not arise from differences in the chemical properties. For electrodes modified with magnetized composites, the rate of HER is higher than the corresponding Nafion film. For demagnetized composites, the rate is the same as Nafion or worse. This is consistent with no rate enhancement without a magnetic gradient imposed by a magnetized microparticle. For demagnetized composites, the response is no better than a Nafion film and usually slightly worse. Microparticles block access to the electrode surface and so can reduce flux to the electrode and so current density. This occurs with magnetically modified electrodes as well but the rate enhancement is sufficient to overcome any diminution in current density associated with microparticles that block the electrode surface.

Plots for the individual electrodes discussed in the text are shown. Representative voltammograms are shown throughout. Composites are compared to Nafion films rather than unmodified electrodes to maintain the same proton concentrations (equation 4).

Magnetically modified electrodes evolve hydrogen at higher rates than Nafion films as shown by more positive onset potentials (lower overvoltage) for fixed $j(E)$ and by higher $j(E)$ for fixed E. Measurements of potential are made at the low current density of 0.4 mA cm^{-2} because electron transfer kinetics dominate at lower current densities. Mass transport dominate at higher current densities. Throughout, for a given diamagnetic electrodes, hydrogen is more easily evolved at magnetized composites than Nafion films, where at fixed current density, $\Delta E = E_{mag} - E_{Naf} > 0$. For demagnetized composites, $\Delta E_{demag} = E_{demag} - E_{Naf} \lessapprox 0$.

Voltammetry at glassy carbon electrodes is evaluated at Nafion films and composites formed with each 1 μm γ-Fe_2O_3 (CX) microparticles and in house made 5 μm γ-Fe_3O_4 microparticles. LSVs for GC electrodes are shown in Figure 2a, b, and c for Nafion films, magnetized composites, and demagnetized composites. Magnetized γ-Fe_2O_3 C1 (blue solid), C3 (olive solid), and C8 (teal solid) composites are shown in Figure 2a [4]. Analogous demagnetized composites are shown in Figure 2c. Nafion and larger 5 μm, magnetized Fe_3O_4 composites are shown [5] in Figure 2b.

LSV Data for Magnetized and Demagnetized Composites of CX γ-Fe_2O_3 Microparticles and Nafion Films. Linear sweep voltammograms are shown at 50 mV/s at 0.452 cm^2 glassy carbon electrodes in nitrogen degassed 0.1 M HNO_3. Electrodes are unmodified (yellow - •• -) and modified with Nafion films (red dots) and composites of Nafion and CX microparticles that are either magnetized (solid lines) or demagnetized long dashes. The CX particles are 1 μm in diameter and contain $\gamma-Fe_2O_3$ and alkyl siloxane For CX, X is the number of carbons in the siloxane. In the figure, C1 methyl (blue), C3 propyl (olive), and C8 octyl (teal).

From the Figure SI.3, all magnetized composites catalyze HER with greater efficiency than Nafion film. All demagnetized composites are comparable or slightly less efficient than Nafion films. The volume magnetic susceptibilities (Table SI.3) of the microparticles rank as C1 $>$ C3 $>$ C8. Nafion is diamagnetic. The current density and electrocatalytic rate increases with volume magnetic susceptibility for the magnetized particles.

In Figure SI.3, the top panel reports current density with potential for magnetized C1, C3, and C8 ferrimagnets, all of which have higher rates of electron transfer than Nafion. The bottom panel shows the corresponding results when the C1, C3, and C8 microparticles are demagnetized. For demagnetized particles, rate is not enhanced and performance is comparable to slightly worse than Nafion films.

With higher magnetic content, rates of HER increase and $\Delta E > 0$ for the three magnetized composites. For demagnetized particles, $\Delta E_{demag} < 0$ consistent with a decrease in current density with the demagnetized particles. The 3 demagnetized samples are coincident once corrected for background, consistent with the microparticles blocking access to the electrode in a similar manner for all three demagnetized particles. Demagnetized particles have little impact on the current density other than to decrease current density below Nafion films because access to the electrode is blocked.

LSV Data for Magnetized Fe_3O_4 Composites and Nafion Films. Linear sweep voltammograms are shown at 100 mV/s at 0.452 cm^2 glassy carbon electrodes in nitrogen degassed 0.1 M HNO_3. Approximately 5 μm diameter Fe_3O_4 microparticles are silane coated in house (SI.3.1.2). Composites contain 15 % by volume microparticles in Nafion.

In Figure SI.4, electrodes are modified with Nafion films (red dots) and composites of Nafion and magnetized Fe_3O_4 microparticles (blue solid lines).

From Figure SI.4, the magnetized composite catalyze HER with greater efficiency than Nafion film as shown by the onset of hydrogen evolution at $+280$ mV for the magnetic composite relative to the Nafion film at 0.4 mA/cm^2. The reported current densities are normalized by the geometric area of the electrode. Because heavy Fe_3O_4 microparticles settle on the electrode to block electrode access, the electrochemical surface area (ECSA) is less than the geometric surface area (SI.5.1.3). If current were normalized by ECSA, reported current density would be higher. If corrected to ECSA, ΔE maybe $\gtrsim$0.3 V.

Comparison of Nafion Films and Magnetic Composites on GC For γ-Fe_2O_3 composites, rate scales as C1 $>$ C3 $>$ C8 $>$ Nafion film, which is consistent with increase of HER rates with magnetic content. Voltammetric

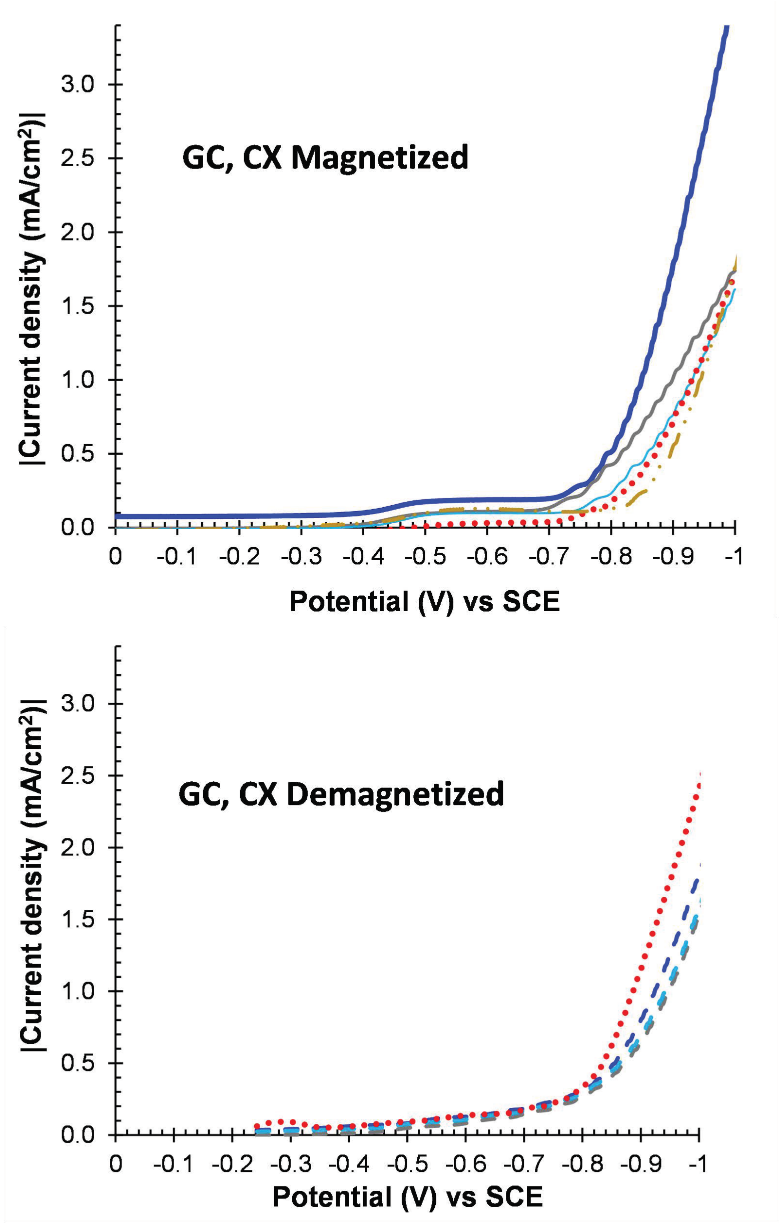

Figure SI.3: Linear sweep voltammograms at 50 mV/s for 0.452 cm^2glassy carbon electrodes modified with with 15 % magnetized γ-Fe_2O_3 microparticles are shown in the top pane for Nafion C1(bright blue wide solid), C3 (olive medium solid), and C8 (light blue narrow solid); a Nafion film (red dot); and an unmodified Pt in 0.1 M HNO_3 (orange dash). HER is catalyzed at the magnetically modified electrodes as compared to Nafion composites. In the lower pane, HER at Nafion film GC electrodes is comparable to or slightly more facile than at composites formed with magnetized and demagnetized materials. The magnetized and demagnetized composites are chemically the same; the presence of the magnetic field gradient increases the rate of HER.

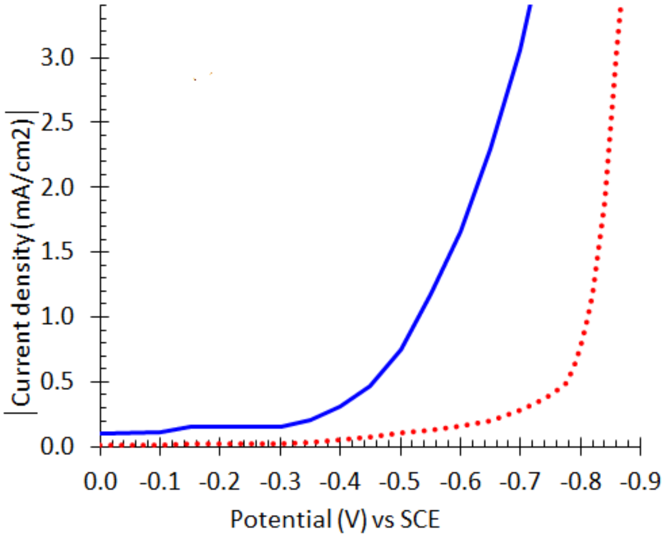

Figure SI.4: Cyclic voltammograms at 100 mV/s for 0.45 cm^2 glassy carbon electrodes modified with 15 % 5 μm magnetized Fe_3O_4 ferromagnetic microparticles in Nafion (red short dash) and Nafion films (green long dash). Electrolyte is degassed 0.1 M HNO_3. At 0.4 mA/cm^2, $\Delta E = 280$ mV.

morphologies for magnetized Fe_3O_4 (Figure 2b) and γ-Fe_2O_3 (Figure 2a) composites are similar, but HER rates are higher for the Fe_3O_4 composites.

To estimate relative rates of HER electrocatalysis, differences in potential for electrodes modified with magnetized composites E_{mag} and Nafion films E_{Naf} are determined at a fixed current density of 0.4 mA cm^{-2} (SI.2.2). For $\Delta E = E_{mag} - E_{Naf} > 0$, electrocatalytic rate is higher for magnetized composites. For example in Figure 2a, the potential to drive HER is less extreme for magnetized C1 composites than Nafion films and E_{mag} is positive of E_{Naf}.

Free energy ΔG relates to ΔE as $\Delta G = -F\Delta E$ at a fixed current density. For replicate measurements on at least three electrodes, magnetized C1 composites and Nafion films yield $\Delta E = E_{mag} - E_{Naf} = 0.19_1 \pm 0.01_9$ V. Dihydrogen evolves more readily at magnetically modified GC electrodes, where ΔG is -18.$_4$ kJ mol^{-1}, which corresponds to an increase in HER rate of $\approx 10^2$ fold. For the magnetized Fe_3O_4 composite (Figure 2b), $\Delta E = 0.28$ V with corresponding ΔG of -28 kJ mol^{-1} and rate is increased $\approx 10^{4.7}$ fold.

The magnetic content of γ-Fe_2O_3 composites are characterized by χ_v (SI.3.1.2). Nafion is diamagnetic and χ_v ranks C1 > C3 > C8 for the composites (SI.4). In Figure SI.5, ΔE increases linearly with χ_v (μcgs) as $\Delta E = (1.180 \pm 0.003) \times 10^4\ \chi_v + (2.9_4 \pm 3.2) \times 10^{-2}$ with $R^2 = 0.998_7$. Or, ΔG scales with χ_v as $\Delta G = -1.14 \times 10^9 \chi_v$, so ΔG decreases linearly with χ_v.

For rate constant j$_0$, ΔE of 0.19 V and 0.28 V and $\alpha = 0.5$ estimate j_0^{mag}/j_0^{Naf} of 40 and 230 for magnetized C1 and magnetized Fe_3O_4 composites relative to Nafion films (equationSI.9). Addition of magnetized microparticles to Nafion on diamagnetic GC electrodes increases j_0 by more than an order of magnitude and decreases overpotential by $\gtrsim 0.19$ V. See Table 1. For magnetized C3 and C8 composites (Table SI.3 and Figure SI.5), ΔE values are +123 and +111 mV and for $\alpha = 0.5$, j_0^{mag}/j_0^{Naf} is 11.0 and 8.7, respectively. j_0 increases exponentially with magnetized content.

Comparison of Magnetized Composites and Demagnetized Composites on GC Microparticles are demagnetized by gentle agitation of the microparticle suspension for several hours (SI.3.1.2). GC electrodes are modified with composites of Nafion and demagnetized microparticles. Demagnetized composites evolve H_2 no better than Nafion films, Figure 2c. Current densities and HER rates for demagnetized composites are slightly lower than for Nafion at more extreme potentials as the microparticles partially block access to the electrode surface. Magnetized particles similarly block electrode access, but the magnetic gradient augments electron transfer rate sufficiently to yield net enhanced HER rate.

The chemical matrix for magnetized composites and demagnetized composites is the same; the distinction is physical with the presence and absence of magnetic gradients. In Figure 2c, three composites formed with demagnetized particles, C1-demag (dashed blue), C3-demag (dashed olive), and C8-demag (dashed teal) are chemically the same as the magnetized C1, C3, and C8 composites of Figure 2a. Magnetized composites all yield higher HER rates than Nafion. Demagnetized composites, all yield slightly lower HER rates than Nafion $(\Delta E_{demag} = E_{demag} - E_{Naf} \leq 0)$. $\log j_0$ for magnetized and demagnetized composites differ with statistical confidence $> 99.9\%$. The magnetized and demagnetized composites are chemically the same but differ by the presence and absence of magnetic gradients.

Increase in rate on GC with magnetic content The magnetic susceptibilities measured for composites of 15 % (vol/vol) SiMag C1, C3, and C8 microparticles and Nafion films are shown in Table SI.3. Relative χ_v^{rel} are normalized by volume magnetic susceptibility χ_v (cm^3 mol^{-1}) for the the C1 composites. The values of $\Delta E = E_{mag} - E_{Naf}$ measured at 0.4 mA cm^{-2} on GC are also shown. The measured χ_v for the microparticle composites estimates the net aligned, unpaired spins on the electrode surface in magnetized composites.

A plot of ΔE with χ_v^{rel} is shown in Figure SI.5. Regression yields $\Delta E\,(V) = (0.190 \pm 0.005)\,\chi_v^{rel} + (0.002_9 \pm 0.003_2)$ with $R^2 = 0.9987$. Then, $\Delta E\,(V) = 0.190\chi_v^{rel}$ or $\Delta E\,(V) = 0.0118\chi_v(cgs)$ within a few percent. The electrocatalytic impact on rate measured as ΔE increases linearly with χ_v. As $\Delta G = -F\Delta E$, the free energy also decreases linearly with χ_v. Similarly, $\log j_0^{mag}/j_0^{Naf}$ increases linearly with χ_v or j_0^{mag}/j_0^{Naf} increases exponentially with the unpaired spins introduced to the electrode with magnetic microparticles.

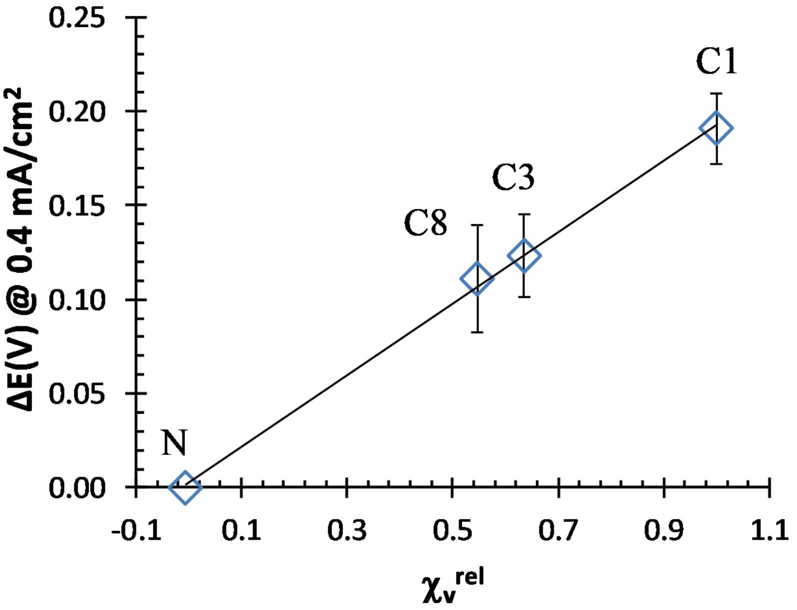

Figure SI.5: A plot of $\Delta E = E_{mag} - E_{Naf}$ with magnetic content of the CX ferri-micromagnets on glassy carbon is linear with volume magnetic susceptibility of the microparticles χ_V. Nafion is on also shown. Regression yields $\Delta E = (1.180 \pm 0.003) \times 10^4\ \chi_v + (2.9_4 \pm 3.2) \times 10^{-2}$ V with $R^2 = 0.9987$. Error bars are standard deviations. Because ΔE is linear with magnetic content of the microparticles, j_0 increases exponentially.

Fe_3O_4 composites formed at 15 % (vol/vol) on glassy carbon, ΔE of 0.28 V is found. Although χ_v^{rel} for the Fe_3O_4 composites is not measured, the regression $\Delta E\,(V) = 0.190\chi_v^{rel}$, would estimate χ_v^{rel} for the Fe_3O_4 composites of about 1.5. The saturation magnetization of Fe_3O_4 is slightly higher than that of $\gamma-Fe_2O_3$ (1.125 to 1.3 fold). As manufactured, the smaller Si-Mag microspheres have a reported density of 2.25 g cm^{-3} +[Chemicell product literature http://www.chemicell.com/products/microparticles/docs/PI_SiMAG-Silanol_1101.pdf] and given the density of 4.90 g cm^{-3} for $\gamma-Fe_2O_3$, the microspheres likely have substantial non-ferrimagnetic content. For in-house coated particles, the core is Fe_3O_4. The Fe_3O_4 microparticles may introduce more magnetic material to the electrode surface than the $\gamma-Fe_2O_3$ microspheres.

SI.4.1 Glassy Carbon Data on Figure 1

The impact of magnetized composites on HER rate is overlaid on Figure 1 as a shift in $\log j_0$ from the diamagnetic line. The increase in $\log j_0$ is shown as upward arrows for γ-Fe_2O_3 (blue) and Fe_3O_4 (gray).

From the literature, the work function for GC is 4.61 eV. [53]. To estimate $\log j_0$ for the diamagnetic GC, the regression line for the diamagnetic metals is used, $\log j_0^{diam} = (6.5_6 \pm 0.5_6)\,\Phi\,(eV) - (38._5 \pm 2._4)$. This yields $\log j_0$ for GC of $\approx$ -8.3.

Modification with magnetized composites of γ-Fe_2O_3 and Fe_3O_4 increases the rate as marked by $\Delta E = E_{mag} - E_{Naf}$. From equation SI.11, the shift of $\log j_0^{mag}$ relative to the diamagnetic line is estimated. Transfer coefficient α is estimated as 0.5.

For C1 γ-Fe_2O_3, ΔE is $+0.19$ V, which shifts $\log j_0$ up by 1.60 units (blue arrow).

For Fe_3O_4 composites, ΔE is $+0.28$ V, which shifts $\log j_0$ up by 2.36 units (gray arrow).

Magnetized C1 composites suffice to increase HER rate by 40 times as compared to diamagnetic Nafion films.

Magnetized Fe_3O_4 composites provide sufficient gradient that HER rate shifts from the diamagnetic line to the line for metals with unpaired spins. The is consistent with magnetic modification of diamagnetic electrodes increasing electrocatalytic HER rates to values comparable to electrodes with inherent spin. Shifts of $+0.27$ to $+0.28$ V have also been observed for p-Si and n-GaAs modified with magnetized Fe_3O_4 composites.

Paramagnetic Pt modified with magnetized C1 composites does not differ statistically from the Nafion modified electrode. This is marked as an open black circle.

From as yet unpublished data, it is noted that j_o for unmodified GC electrodes is higher than calculated from literature work function by about a factor of ten. (Joshua Coduto, University of Iowa).

SI.4.2 Synopsis of Results for Diamagnetic Electrodes Modified with Nafion, Magnetized Composites, and Demagnetized Composites

The noted magnetic impact on $\log j_0$ in the literature data of Section 1.1 and Figure 1 prompted experimental studies (Section 2.1). The experiments were undertaken to characterize and exploit magnetic impacts on electron transfer and electrocatalysis. Electrodes modified with Nafion films, composites of magnetized microparticles in Nafion, and demagnetized composites are compared. Four experimental outcomes are noted. Electrocatalytic HER rates are substantially increased at diamagnetic cathodes and photocathodes modified with magnetized composites as compared to Nafion films and demagnetized composites (Section 2, Figure 2, Table 1).

Experiments are undertaken to determine if HER rates are increased by introduction of electron spin to the surface of electrodes with magnetized microparticles. For fixed current density, potentials at electrodes modified with Nafion films E_{Naf} and magnetized E_{mag} and demagnetized E_{demag} composites are compared as $\Delta E = E_{mag} - E_{Naf}$ Nafion in the films and composites maintains the same proton concentration. For $\Delta E > 0$, introduction of magnetized microparticle composites increases rate as compared to the diamagnetic Nafion film with energetic advantage $\Delta G = -F\Delta E$. For diamagnetic cathodes and photocathodes, introduction of electron spin and magnetic gradients substantially increases HER rate (Table 1 and Figure 2).

It is important to note that magnetized and demagnetized composites are chemically the same; the magnetic effect arises from the physical impact of the magnetic gradient (Figure 2a and c).

SI.4.2.1 Magnetic Modification of Diamagnetic Metal Electrodes without Inherent Spin Increases HER Rate

To determine if the magnetoelectrocatalytic effect of unpaired electron spins can be induced at diamagnetic electrodes, magnetic gradients are imposed on diamagnetic cathodes and photocathodes (Section 2). Glassy carbon, gold, and mercury pool cathodes and n-GaAs and p-Si photocathodes are modified with either a Nafion film or a composite of magnetized microparticles in Nafion. Siloxane coating renders the microparticles chemically and electrochemically inert. Microparticles are either magnetized or demagnetized. For all diamagnetic electrodes, HER electrocatalysis is more efficient at diamagnetic electrodes modified with magnetize composites than modified with Nafion films. See for examples, Table 1, Figure 2, and SI. At low current density (4 mA cm^{-2}), demagnetized composites evolve hydrogen at potentials similar to but no greater than Nafion films (Figure 2c and d). Because demagnetized microparticles partially block access to the electrode surface, current density is decreased slightly as compared to Nafion. That rates are different on magnetized and demagnetized composites that are chemically the same highlights the physical impact caused by the magnetic gradients.

Addition of magnetized microparticles to the surface of diamagnetic electrodes introduces electron spin and magnetic properties that catalyze electron transfer for HER (Figure 2 and Table 1).

The response scales with micromagnet properties. For the γ-Fe_2O_3 CX particles, ΔE scales linearly with the measured volume magnetic susceptibility of the particles, χ_V for GC (Figure SI.5) and Au. [4]

Magnetized Composites and Nafion Films on Diamagnetic Electrodes The magnetic gradient about the microparticles enhances HER rates. Impacts vary with the microparticle (Fe_3O_4 $>$ C1 $>$ C3 $>$ C8 $>$ Nafion $\gtrsim$ demag-C1, demag-C3, demag-C8). In Table 1, $\Delta E = E_{mag} - E_{Naf} > 0$ in all cases for magnetize C1 ($\gamma$$-$$Fe_2O_3$) and Fe_3O_4 composites as compared to Nafion films. For GC, Hg pool, n-GaAs, and p-Si, $0.19 \leq \Delta E \lesssim 0.4$ V and ΔG changes by -18 to -42 kJ/mol with $\alpha^{-1} \log j_0^{mag}/j_0^{Naf}$ of 3.2 to 7.4. For α estimated as 0.5, $j_0^{mag}/j_0^{Naf}|_{\alpha=0.5}$ is also reported in Table 1. Magnetized microparticles on diamagnetic electrodes consistently and substantially increases HER rates by magnetoelectrocatalysis. Of the electrodes studied, two (Au and Hg) are metals, two are semiconductors (n-GaAs and p-Si), and GC is perhaps best described as a semi-metal.

The focus here is on diamagnetic glassy carbon electrodes.

At GC for magnetized C1 composites, $\Delta E = +0.19$ V, $\Delta G = -18$ kJ/mol, and $j_0^{mag}/j_0^{Naf}|_{\alpha=0.5} = 40$. Enhancements in HER rates with magnetized C1 microparticles on GC electrodes are comparable to the differences in j_0^{spin} and j_0^{diam} at a given Φ in Figure 1. For GC modified with magnetized Fe_3O_4 composites, magnetic impacts are larger with $\Delta E = 0.28$ V, $\Delta G = -27$ kJ/mol, and $j_0^{mag}/j_0^{Naf}|_{\alpha=0.5} = 230$. GC cathodes and n-GaAs and p-Si photocathodes that are modified with magnetized Fe_3O_4 composites have largely invariant enhancements with ΔE of $+270$ to $+280$ mV, $\Delta G = -27$ kJ/mol, and $j_0^{mag}/j_0^{Naf}|_{\alpha=0.5} = 210$ to 230. For all diamagnetic electrodes, that magnetized composites enhance HER rates as compared to Nafion films is vetted with statistical confidence of 90 % to > 99.9 %.

Magnetized Composites and Demagnetized Composites on Diamagnetic Electrodes A critical comparison in these studies is the comparison of magnetized composites with demagnetized composites because the composites have the same chemical properties but distinct physical properties.

To avoid fracture of brittle magnetic materials, microparticles are demagnetized by gentle agitation in Nafion suspension to prevent liberating iron ions into the composite matrix. Electrodes are modified with either magnetized or demagnetized composites. Once formed, composites are not switched between the magnetized and demagnetized states to avoid mechanical disruption of the Nafion matrix.

Compared to Nafion, demagnetized composites yield E equal to or slightly negative of E for Nafion films. To the extent that microparticles block access to the electrode surface, current density is decreased. For fixed current density, $\Delta E_{demag} = E_{demag} - E_{Naf} \lesssim 0$ and no rate enhancements are observed at diamagnetic electrodes modified with demagnetized films. Voltammetric morphologies are similar to Nafion films, consistent with no iron ions free in the composite to mediate HER.

For all diamagnetic electrodes, comparison of magnetized and demagnetized composites invariably yields $E_{mag} - E_{demag} > 0$ with values equal to or a few millivolts greater than $\Delta E = E_{mag} - E_{Naf}$.

That $E_{mag} - E_{demag} > 0$ and comparable to ΔE is critical. The magnetized and demagnetized composites have the same chemical matrix and differ only in the physical distinction of the presence and absence of a magnetic field and gradient. In the presence of the magnetic field and gradient, rate of HER increases. $E_{mag} - E_{demag} > 0 \gtrsim \Delta E$ establishes that the magnetic properties of the composite enhance electrocatalysis of HER.

SI.5 Results for Paramagnetic Platinum

Data for HER at paramagnetic Pt modified with Nafion and magnetized composites are evaluated. Within experimental uncertainty, electrocatalysis and thermodynamics are unaffected by magnetized composites on Pt. .LSV experiments are reported for γ-FeO C1 composites under a nitrogen blanked and FeO composites under a hydrogen blanket. Open circuit potentials are also measured under an H_2 blanket.

SI.5.1 LSV on Pt

LSV experiments are undertaken for Nafion films and magnetized composites of 1 μm $\gamma$$-$$Fe_2O_3$ microparticles under a nitrogen blanket in 0.10 M HNO_3 and 5 μm Fe_3O_4 microparticles under a hydrogen blanket in 1.0 M

HNO_3.

In LSV measurements, electron transfer kinetics dominate voltammetric response at low current densities. At higher current densities and longer times, proton concentration in solution is perturbed from its initial value as the diffusion length extends beyond the thickness of the modifying layer. At higher current densities, current density may be impacted by mass transport in the electrolyte, where transport effects may be magnetically dependent. Magnetohydrodynamics (MHD) is an example of transport driven by a Lorenz force. [14–16]. For diffusion length confined within the modifying layer, the nanostructure of Nafion precludes the fluid flow required for MHD transport. No magnetic effects on transport are observed at low current densities for modified Pt.

SI.5.1.1 $\gamma-Fe_2O_3$ Composites under N_2 Blanket in 0.10 M HNO_3 [4]:

LSV for Pt modified with Nafion films and 15 % vol/vol magnetized C1, C3, and C8 composites in N_2 degassed 0.1 M HNO_3 are shown in Figures 2d and SI.6. [4] Electron transfer kinetics dominate voltammetric response at low current densities below ~ 1 mA/cm^2. No evidence of magnetically facilitated electron transfer are found at low current densities, consistent with superimposed voltammograms for the varied magnetic content in the composites and Nafion film. In Table 1 for 0.4 mA cm^{-2}, replicate measurements for magnetized C1 composites yield $\Delta E = E_{mag} - E_{Naf}$ of $+(0.007 \pm 0.005)$ V. With > 90 % confidence, there is no statistical difference in E_{mag} and E_{Naf} . Kinetics on paramagnetic Pt are not enhanced by introduction of magnetized microparticles to the electrode surface. At higher current densities, mass transport affects current density (SI.5).

In Figure SI.6, data are shown for Pt under a nitrogen blanket in 0.1 M HNO_3 at a scan rate of 0.050 V/s. [4] Composites are formed with 15 % magnetized γ-Fe_2O_3 (C1, C3, and C8) 1 μm microparticles in Nafion. Examination of C1, C3, and C8 composites checks for variation in response with magnetic content. The current density is lower for the unmodified Pt electrode than the Nafion electrode because the 0.1 M proton concentration in the electrolyte is below the estimated 1.6 M proton in Nafion. [55, 80, 81] The upper panel includes current densities up to 20 mA cm^{-2}, where at high current densities $\gtrsim$10 mA cm^{-2}, there is some dispersion likely due to disruption of the solution boundary layer by H_2 bubble formation and perhaps other mass transport effects. The nanostructure inside Nafion is on the order of 5 nm [92, 93], which precludes the bulk solvent motion need for mass transport to deliver convection by magnetohydrodynamics.

Mass transport effects are observed at higher current densities but electron transfer kinetics are assessed at low current densities. The lower panel and Figure SI.6 are the same data enlarged to view the lower current density data below 5 mA cm^{-2}. For current density of $\lesssim 2$ mA/cm^2, the superimposed voltammograms for Nafion and magnetized composites disallow magnetically induced transport effects. The overlay of the Nafion film and magnetized γ-Fe_2O_3 composite curves is consistent with no magnetic impacts near the formal potential $E^{0'}$ for HER on Pt. This is consistent with the anticipated independence of HER thermodynamics on Pt electrode on magnetic modification.

SI.5.1.2 Fe_3O_4 Composites under H_2 Blanket in 1.0 M HNO_3 [5]:

From LSVs at Pt modified with a Nafion film and a 20 % vol/vol magnetized Fe_3O_4 composite in H_2 degassed 1.0 M HNO_3, the magnetized composite does not enhance HER rate on Pt as compared to a Nafion film (Figure SI.7 and Table 1). Larger 5 μm magnetite microparticles also partially block access to the Pt electrode to lower current density. Again, $\Delta E = +0.001$ V with experimental uncertainty of 1 to 2 mV.

In Figure SI.7, composites of 20 vol/vol % of 5 μm Fe_3O_4 (black solid line) are compared to Nafion films (blue dots) at Pt electrodes in 1 M HNO_3 under hydrogen gas. LSV are also shown for unmodified Pt electrodes (red dash). The currents for the unmodified Pt are higher here than in Figure SI.6 because the proton concentration in the electrolyte is higher. The 1 M proton in the electrolyte approaches the estimated 1.6 M proton in Nafion *[12, 55]*, which yields currents at the unmodified Pt only slightly lower than the currents for the Nafion film on Pt.

Voltammograms are reported as current (mA) evolved as potential E vs SCE is swept negative to drive dihydrogen evolution on 0.452 cm^{-2} Pt electrodes. As detailed below for current measured from background, currents for the magnetized composite are consistently and proportionally lower than currents for Nafion films. These results are consistent with HER electrocatalysis on Pt that is not enhanced on introduction of magnetized Fe_3O_4 microparticles. No impact on magnetic modification is the same outcome as found for $\gamma-Fe_2O_3$ composites on Pt under nitrogen. Because Pt is paramagnetic, Pt inherently has unpaired spins that support electrocatalysis. HER kinetics on Pt are fast and addition of electron spins from magnetized microparticles does not further

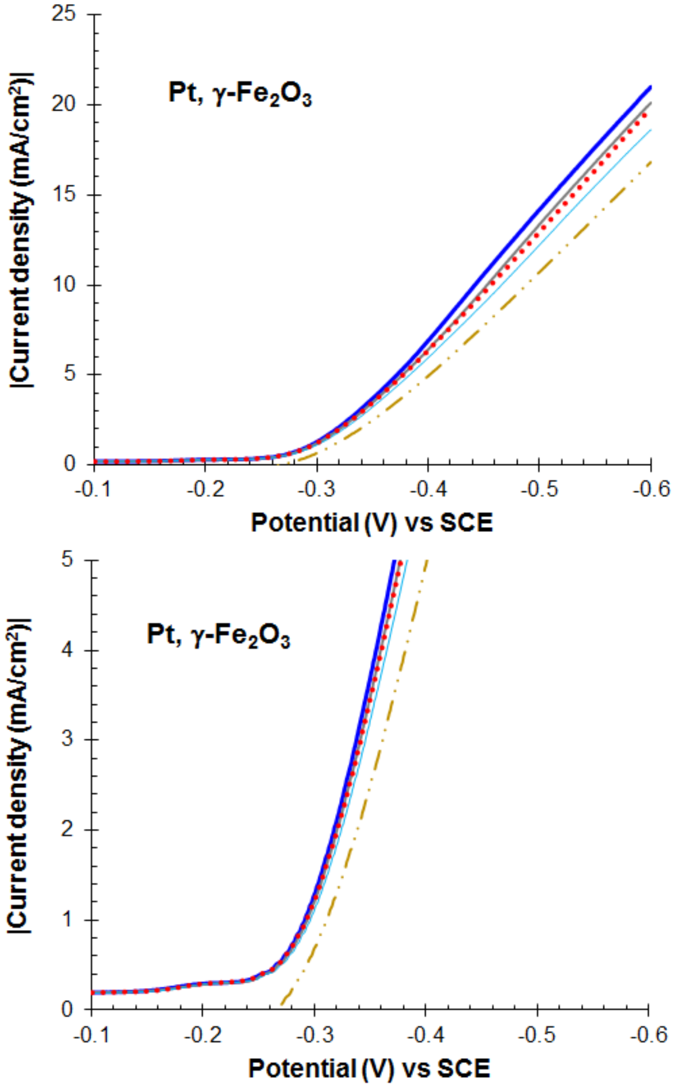

Figure SI.6: Linear sweep voltammograms at 50 mV/s for 0.452 cm^2 paramagnetic platinum electrodes modified with 15 % magnetized ferrimagnetic γ-Fe_2O_3 magnetized microparticles in Nafion C1 (blue wide solid), C3 (blue medium solid), and C8 (light blue narrow solid); a Nafion film (red dot); and an unmodified Pt in 0.1 M HNO_3 (orange dash) under a nitrogen blanket. The upper plot includes higher current densities where mass transport effects can be observed above $\gtrsim$ 10 mA cm^{-2}. The lower plot is shown for lower current density $\lesssim$ 5 mA cm^{-2}. For current densities $\lesssim$ 2 mA cm^{-2} , the curves overlay for the Nafion film and all the magnetic composites, which is consistent with no impact on HER rate about the formal potential.

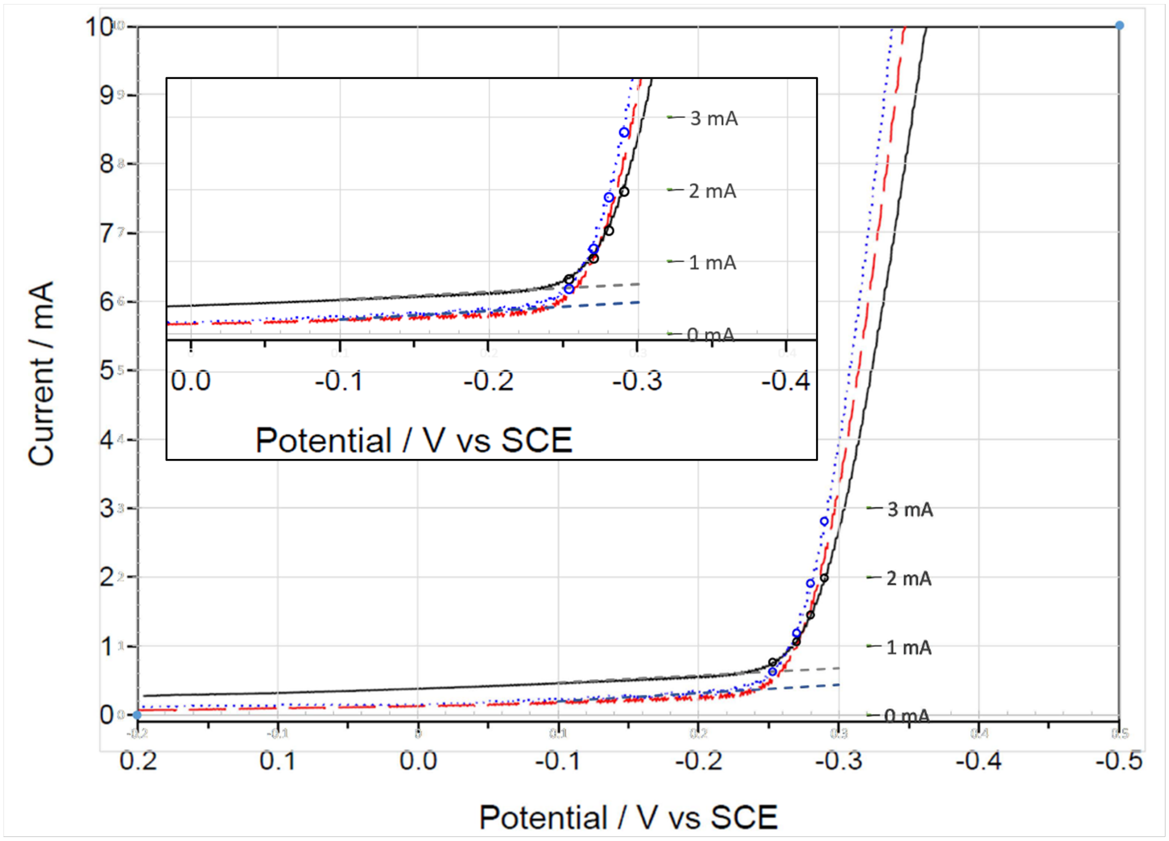

Figure SI.7: LSVs of hydrogen evolution on 0.45 cm^2 Pt electrode that is unmodified (dashed red), modified with a Nafion film (dotted blue) and with magnetized composite of a 20 % (v/v) Fe_3O_4 in Nafion (solid black). Electrolyte is 1.0 M HNO_3 under a hydrogen blanket at 100 mV/s. The data are enlarged in the inset. Background currents for the composite (gray dash) and Nafion film (blue dash) are shown. Currents measured at potentials between -0.25 to -0.29 V vs SCE are corrected for background to yield a fixed current ratio, $i^{mag}/i^{Naf} = 0.530 \pm 0.01_6$. The current ratio constant across several potentials indicates 47% of the active electrode surface area is blocked by Fe_3O_4 particles and the exchange current density j_0 is the same for the Nafion film and the magnetized composite. Unlike diamagnetic electrodes, addition of magnetized microparticles to paramagnetic Pt does not impact electron transfer rate.

accelerate the rate. HER on Pt may not be under sufficient kinetic control to make the measurement under these conditions. Alternatively, the gradient about the microparticles may be negligible compared to Pt atoms (SI.6.4).

Fe_3O_4 Microparticles Block Electrode Surface. Fe_3O_4 particles are dense and can settle on the electrode during casting to impact electrochemical surface area (ECSA). Currents are measured from background for both Fe_3O_4 composites and Nafion films on Pt. Extrapolations of the background current i_{bckgnd} are shown in Figure SI.7 as blue dashed lines for Nafion ($i^{Naf}_{bckgnd}(E) = -1.15E + 0.085$) that lies just below the grey dashed lines for Fe_3O_4 composites ($i^{Fe_3O_4}_{bckgnd}(E) = -1.05E + 0.365$). The magnetite composite has a higher background current than the Nafion films. Currents i_{meas} are measured at 0.253, 0.27, 0.28, and 0.29 V vs SCE and the background currents are calculated at E and subtracted to yield the background corrected currents $i^{Naf}_{corr}(E) = i^{Naf}_{meas}(E) - i^{Naf}_{bckgnd}(E)$ and $i^{mag}_{corr}(E) = i^{mag}_{meas}(E) - i^{mag}_{bckgnd}(E)$. Measured currents are shown as blue circles for Nafion and black circles for Fe_3O_4 composites. Data are tabulated in Table SI.4 for the background corrected current i_{corr}. Consistently, $i^{Naf}_{corr} > i^{mag}_{corr}$ consistent with no increase in rate on introduction of magnetic particles.

A magnetically enhanced rate would increase the current at the magnetically modified electrode relative to Nafion.

From Equation 3,

$$j(E) = j_0 \exp\left[-\alpha f\left(E - E_{eq}\right)\right] \quad ((7))$$

Potential	Nafion			Fe$_3$O$_4$			
(E vs SCE)	i^{Naf}_{meas} (mA)	i^{Naf}_{bckgnd} (mA)	i^{Naf}_{corr} (mA)	i^{mag}_{meas} (mA)	i^{mag}_{bckgnd} (mA)	i^{mag}_{corr} (mA)	$i^{mag}_{corr}/i^{Naf}_{corr}$
-0.253	0.62	0.38	0.24	0.76	0.63	0.13	0.54
-0.270	1.18	0.40	0.78	1.05	0.65	0.40	0.51
-0.280	1.90	0.41	1.49	1.44	0.66	0.78	0.52
-0.290	2.80	0.42	2.38	1.98	0.67	1.31	0.55
-0.258				0.82	0.63_6	0.18_4	
-0.252	0.56	0.37_5	0.18_5				

Table SI.4: Measured $i(E)$ and E for Nafion and composites of 20 % (vol/vol) Magnetized Fe_3O_4 Microparticles in Nafion. Current measured at 0.4 mA cm^{-2} is corrected to background. The ratio of corrected currents $i^{mag}_{corr}/i^{Naf}_{corr}$ estimates the microparticles block 50 to 55 % of the electrode surface.

if two reactions have similar E_{eq} and α values but different j_0, as $E - E_{eq}$ is more extreme, $j\left(E\right)$ escalates more rapidly for the faster j_0. For fixed values of E where electron transfer limits the current, the ratio of current densities increases as $E - E_{eq}$ is more negative. But in Table SI.4 where both electrodes are 0.45 cm^2, the ratio $i^{mag}_{corr}/i^{Naf}_{corr} = 0.53 \pm 0.01_6$, is invariant with E. For magnetized Fe$_3$O$_4$ composites on Pt, the current density ratio is fixed and < 1 because the electroactive surface area is partially blocked by Fe$_3$O$_4$ microparticles settled on the electrode surface. For $i^{mag}_{corr}/i^{Naf}_{corr} = 0.53 \pm 0.01_6$, access to 47% of the electrode surface is blocked. The exchange current densities for the Pt modified with Nafion and with magnetized Fe$_3$O$_4$ composites are the same.

The data in Table SI.4 are current density ratios measured at fixed values of E.The data are converted to E_{mag} and E_{Naf} at fixed current density of 0.4 mA cm^{-2} of active electrode area (electrochemical surface area, ECSA) as follows. For Pt electrodes with geometric area of 45 cm^2, 0.181 mA corresponds to current density of 0.4 mA cm^{-2}. From Figure SI.7 and Table SI.4, this estimates $E_{Naf} = -0.252$ V and $E_{mag} = -0.258$ V for the geometric area. For the Nafion modified electrode, the geometric area well approximates ECSA, but for the magnetized Fe$_3$O$_4$ composite, $i^{mag}_{corr}/i^{Naf}_{corr} = 0.53 \pm 0.01_6$ estimates ECSA for the composite as 0.45 cm^2 $(0.53) = 0.24$ cm^2. For the Fe$_3$O$_4$ composite with ECSA of 0.24 cm^2, current of 0.096 mA corresponds to 0.4 mA cm^{-2}. From Figure SI.7 and Table SI.4, 0.096 mA that corresponds to fixed current density of 0.4 mA cm^{-2} yields $E_{mag} = -0.251$ V for the composites. Given the uncertainties in the measurement of about 1 mV, $E_{mag} = -0.251$ V does not differ from $E_{Naf} = -0.252$ V vs SCE as measured by LSV.

SI.5.1.3 Electrodes Blocked by Microparticles and Electrochemical Surface Area (ECSA)

The ratio $i^{mag}_{corr}/i^{Naf}_{corr}$ is fixed over the potentials in the low current range as $0.53 \pm 0.01_6$. The ratio below 1 is thought to arise because the heavier, larger 5 μm Fe$_3$O$_4$ microparticles in a 6 μm thick composite likely settle to the electrode surface when the composite is cast. The settled, large and dense Fe$_3$O$_4$ microparticles block proton access to the electrode interface. The smaller, less dense γ-Fe$_2$O$_3$ microparticles are better suspended in the casting solvents and less electrode area is blocked as shown in, for example, Figure 2c where for a given potential the current densities for the demagnetized composites are only slightly lower than for the Nafion film in the low current range below 0.5 mA cm^{-2}.

The Fe$_3$O$_4$ microparticles restrict flux to the electrode surface as the cross section of the microparticles disrupts ("blocks") proton diffusion to the electrode.

The electrode geometric area for these electrodes is 0.452 cm^2. The geometric area is the planar area of the disk. Electrochemical surface area ECSA measures how much electrode area can be accessed by the electroactive species, here proton. Flux to the Nafion modified electrode is well characterized by the geometric surface area. ECSA measures accessible surface, which is the current ratio $i^{mag}_{corr}/i^{Naf}_{corr}$ of 0.53 for the larger Fe$_3$O$_4$ microparticles.

Equilibrium measurements such as open circuit potentials (OCPs) are not impacted by ECSA.

SI.5.2 Open Circuit Potential under H$_2$ Blanket on Pt [5]

Open circuit potentials (OCPs) are measured at equilibrium where there is no current flow (no net electron transfer reaction). To compare Nafion films and 20 % magnetized 5 μm Fe$_3$O$_4$ composites on Pt, OCPs (V$_{oc}$) are measured in 1.0 M HNO$_3$ under an H$_2$ blanket. For Nafion and the Fe$_3$O$_4$ composites, V$_{oc}$ of -0.255 and -0.256 V vs SCE

are found. Within the precision of the measurement ($\pm$ 1 mV), ΔE does not differ from zero, consistent with no impact of magnetic fields and gradients on the equilibrium and thermodynamic energies of proton reduction. Results for OCP and LSV measurements on Pt are consistent for paramagnetic Pt, $\Delta E = (0.000 \pm 0.001)$ V

Formal potential $E^{0\prime}$ characterizes the electrochemical system under conditions other than standard conditions relative to the standard potential E^0. Activities of H_2 and H^+ are introduced.

$$E^{0\prime} = E^0 - \frac{2.303RT}{F} \log \frac{a_{H_2}^{1/2}}{a_{H^+}} \tag{SI.14}$$

Values of a_{H^+} and a_{H_2} are expected to differ in 1.0 M HNO_3 and Nafion. The proton concentration in 1.0 M HNO_3 is lower than the estimated 1.6 M in Nafion. [55,80,81] Activity coefficients for proton in different high ionic strengths will also vary. As activity is the product of concentration and activity coefficient, a_{H^+} is expected to be different in Nafion than in solution. For dihydrogen, the activity coefficient is 1, but the saturation concentration of hydrogen in Nafion (~ 10 mM) is higher than in aqueous electrolyte (~ 0.75 mM) [94] and a_{H_2} will also differ. The open circuit potentials for the unmodified Pt electrode and the electrode modified with a Nafion film are expected to differ. For the composites, the electrolyte is the same Nafion matrix as the Nafion film, and the open circuit potentials are not expected to differ.

Open circuit potentials V_{oc} are determined for Pt modified with a Nafion film and a magnetized composite of 20 % Fe_3O_4 microparticles under a hydrogen blanket in 1.0 M HNO_3. [5] Unmodified Pt was also evaluated in the same electrolyte. The uncertainty for open circuit measurements is ± 1 mV. The values for Nafion film, magnetized magnetic composite, and unmodified Pt are shown in Table SI.5.

Electrode	V_{oc} vs SCE	V_{oc} vs NHE
Pt unmodified	-0.251	-0.009
Pt|Nafion film	-0.255	-0.013
Pt|Fe_3O_4+Nafion composite	-0.256	-0.014

Table SI.5: Open circuit potentials under H_2 blanket in 1.0 M HNO_3 for Pt electrodes that are unmodified, modified with a Nafion film, and modified with magnetized Fe_3O_4 Nafion composites.

The open circuit potential for the unmodified Pt and the Nafion film differ by 4 mV. With approximate proton (1.0 M and 1.6 M) and dihydrogen concentrations (0.75 mM and 10 mM) in 1.0 M HNO_3 and Nafion, the activity coefficient for proton in the aqueous electrolyte may be about 40% higher than in Nafion. Activity coefficients can vary widely in high ionic strength ($\gtrsim 1$ M) environments.

Under equilibrium conditions, the measured V_{oc} for Nafion film and magnetized Fe_3O_4 composites are the same within the experimental error of 1 mV. There is no evidence of a magnetic effect on the thermodynamics of the reaction in Reaction 1. Under the hydrogen blanket, magnetic modification does not alter the thermodynamic potentials anticipated on platinum.

SI.5.3 Because Pt Response Is Not Altered by Magnetized Composites Compared to Nafion

At low current densities where current density is limited by the rate of electron transfer, the response at Pt electrodes modified with magnetized composites and Nafion films is the same, from which several observations are noted.

SI.5.3.1 Thermodynamics

Magnetic effects on equilibrium and thermodynamics at room temperature are not expected as magnetic energies are negligible compared to thermal energies. Magnetic effects on chemical systems arise through dynamics and their associated gradients. Dynamics includes chemical kinetics and transport. Measurements at OCP are equilibrium measurements.

OCP measurements are made at Pt in 1 M proton under a hydrogen blanket. The measured open circuit potentials differ by 1 mV, the uncertainty in the measurement. Statistically, the OCP potentials do not differ.

SI.5.3.2 Not Mediated

Because low current densities overlay for Nafion films and magnetized composites on Pt, there is no evidence of chemical mediation by chemical components in the iron oxide particles. The microparticles are chemically and electrochemically inert. No evidence of iron chemistry or electrochemistry has been observed in any of these studies.

The microparticles are either Fe_2O_3 or Fe_3O_4, where the iron species are either Fe^{2+} or Fe^{3+}.In acid electrolyte, Fe^0 is not stable. The standard potentials for the iron species and proton reduction are:

$$Fe^{\dot{3}} + e \rightleftharpoons Fe^2 \qquad E^0 = 0.771 \text{ V vs NHE} \tag{SI.15}$$

$$H^+ + e \rightleftharpoons \frac{1}{2}H_2 \qquad E^0 = 0.000 \text{ V vs NHE} \tag{SI.16}$$

$$Fe^2 + 2e \rightleftharpoons Fe^0 \qquad E^0 = -0.44 \text{ V vs NHE} \tag{SI.17}$$

Thermodynamically, neither Fe^2 nor Fe^3 can mediate proton reduction and H^+ is stable in the presence of Fe^2 and Fe^3. In the presence of Fe^3, Fe^0 reacts spontaneously to form Fe^2. In the presence of acid, Fe^0 reacts spontaneously with acid to form Fe^2 and H_2. Dihydrogen reacts spontaneously with Fe^3 to revert back to proton and Fe^2. Thermodynamically, no net mediation by iron species generates dihydrogen.

Oxygen can react with H_2 and Fe^0.

$$O_2 + 4H^+ + 4e \rightleftharpoons 2H_2O \qquad E^0 = 1.229 \text{ V vs NHE} \tag{SI.18}$$

Reaction with Fe^0 forms Fe^{2+} and then Fe^{3+}. Oxygen reacts with H_2 to reform H^+. Oxygen does not mediate dihydrogen formation, but consumes H_2. With exception of Fe_3O_4 on GC, all solutions are degassed with either nitrogen or hydrogen, and O_2 is displaced.

If HER were mediated by a component introduced with the microparticles, Nafion and composite responses on Pt at low current density would differ. On GC, demagnetized composites do not electrocatalyze proton reduction.

Mediation does not increased hydrogen production rate in these systems.

SI.5.3.3 Not Magnetically Driven Mass Transport Enhancement

On Pt, MHD (magnetohydrodynamics) enhanced current at higher current densities but in the electron transfer controlled regime, currents under voltammetric perturbation are the same for Nafion and magnetized composites. Because of the nanostructure of Nafion, there is no bulk fluid at the electrode surface so that bulk fluid motion is disabled. [16] No magnetically driven mass transport effects are observed.

SI.5.4 Synopsis of No Magnetic Impact on HER Rate on Paramagnetic Pt

Pt: Magnetized composites on paramagnetic Pt electrodes do not impact either HER rates or OCP (Section 2.2.2, Figures 1d and SI.7, and Table 1).

- Thermodynamics of hydrogen at Pt is preserved, as anticipated because magnetic energies are negligible compared to ambient thermal energies.
- There is no evidence of chemical mediation as Pt LSV data are the same with and without microparticles.
- There is no evidence of magnetically driven mass transport as Pt LSV data are the same for Nafion and magnetized iron oxide composites. There is no bulk fluid in Nafion.

SI.6 Results for an External Applied Uniform Magnetic Field

Throughout the studies of magnetized composites on electrodes, a question arose as to whether the magnetic field or the magnetic gradient impacts electron transfer. Magnetic fields of permanent magnets are established by net alignment of electron spins. The magnetized microparticles suffice to sustain magnetic fields, but as the fields dissipate from the microparticle surface, steep, interfacial magnetic gradients are established into the surrounding (Nafion) media. Typically, when magnetic effects are introduced to an electrochemical system, an external magnet

is used and mass transport is enhanced. [14, 15, 35, 37, 40–46, 95]. To differentiate impacts of magnetic fields and magnetic gradients in the composites, an external, uniform magnetic field is applied to modified and unmodified electrodes.

The uniform external field is applied with a hollow cylinder rare earth (neodymium iron boride) ring magnet. Rare earth magnets provide magnetic fields up to 2 T (1 Tesla $= 10^4$ Gauss). Inside the ring, the magnet provides a strong uniform field but minimal field gradient.

The electrochemical cell is snugged into a hollow cylinder magnet with the surface of the Pt disk electrode centered in the magnet at half height to provide a uniform magnetic field with little to no magnetic gradients (Figure SI.2). The experiments are undertaken in 0.64 mM tris(bipyridine) ruthenium (II) chloride $Ru(bpy)_3^{2+}$ (Sigma) and 0.10 M nitric acid HNO_3 (Fischer Chemical) [4]. The redox probe $Ru(bpy)_3^{2+}$ extracts from the electrolyte into Nafion and is immobilized at high concentration by electrostatic binding to sulfonate in Nafion. [79, 80] Under voltammetric perturbation, high probe concentration in Nafion allows electron self exchange (electron hopping) between $Ru(bpy)_3^{2+}$ and $Ru(bpy)_3^{3+}$ that increases current in Nafion. [12, 81] The high concentration of sulfonates in Nafion excludes chloride and nitrate ions. At unmodified electrodes $Ru(bpy)_3^{2+}$, redox moieties are well separated and self exchange does not enhance current. Measurements are made at an unmodified Pt disks and Pt disks modified with Nafion films, magnetized γ-Fe_2O_3 C1 composites, and demagnetized C1 composites. Voltammograms for electrodes with and without the external field of the ring magnet are compared. All Pt electrodes are 0.45 cm^2 geometric area.

SI.6.1 Unmodified Pt Disk and Nafion Film Modified Pt Disk

In Figure SI.3, representative cyclic voltammograms recorded at 200 mV/s are shown for an unmodified platinum disk (top) and for a disk modified with a Nafion film (bottom) for 0.64 mM $Ru(bpy)_3Cl_2$ in 0.10 M HNO_3. Data with no external ring magnet are shown as black lines. The current for the Nafion film is higher than for the unmodified electrode because Nafion concentrations $Ru(bpy)_3^{2+}$ dication well above the solution concentration. Flux in Nafion is facilitated by electron hopping (self exchange) between $Ru(bpy)_3^{2+}$ and $Ru(bpy)_3^{3+}$ that is reflected in higher currents. [81, 87, 88]

Current recorded when the external ring magnet encircles the disk electrodes is shown as a red dashed line. For the unmodified electrode, the current for $Ru(bpy)_2^{2+}$ (~1.05 V vs SCE) increases by ~ 65 % by magnetohydrodynamics. The current at more positive potentials is likely enhanced by magnetohydrodynamic transport effects of the chloride anion in $Ru(bpy)_3Cl_2$. The enhancements at the unmodified electrode are consistent with the strong and uniform magnet field established at the disk surface that interacts with the solution ions to increase bulk solvent motion through magnetic effects on transport .

For the Nafion filmed electrode, the voltammograms with and without the ring magnet superimpose within the width of lines. There is little to no impact of the external magnet on the current measured for the Nafion film electrode. The external ring magnet does not impact the dynamics of either mass transport or electron transfer. Magnetohydrodynamics impacts mass transport by inducing net motion of the bulk solvent. Nafion forms a heterogeneous, micellar matrix of fluorocarbon and aquated domains where the length scale of the biphasic nanostructure is on the order of 5 nm. [55, 92, 93] In the confined nanostructure of Nafion, the volumes and electrostatic interactions within the hydrated domains preclude bulk solvent [55] and its motion. In Nafion and magnetohydrodynamics is not effective.

SI.6.2 Pt Disks Modified with Nafion Films, Magnetized C1 Microparticles Composites, and Demagnetized C1 Microparticles Composites

Magnetized microparticles of iron oxides establish smaller magnetic fields than NdFeB ring magnets, but the magnetic gradients established about the micromagnets are significantly larger. The magnetic field or magnetic flux density $B(r)$ about a magnetized sphere of radius r_0 decays with radial distance $r > r_0$ as $B(r) \propto M(r/r_0)^{-3}$ where the gradient $\partial B(r)/\partial r \propto Mr^{-4}r_0^3$. Magnetization M is the magnetic dipole moment per volume. Near the surface of a magnetized sphere where $0.5 \lesssim r_0 \lesssim 3$ μm, magnetic gradients are far steeper than can be established on the macroscopic scale of a ring magnet or between the plates of common electromagnets.

In Figure SI.8, the LSV at 200 mV s^{-1} for a Nafion film modified electrode for $Ru(bpy)_2^{2+}$ is shown (left) and compared to composites of Nafion with magnetized C1 particles (center) and demagnetized C1 particles (right) without (solid line) and with (dashed line) the uniform field of the external ring magnet. As for Nafion films, the

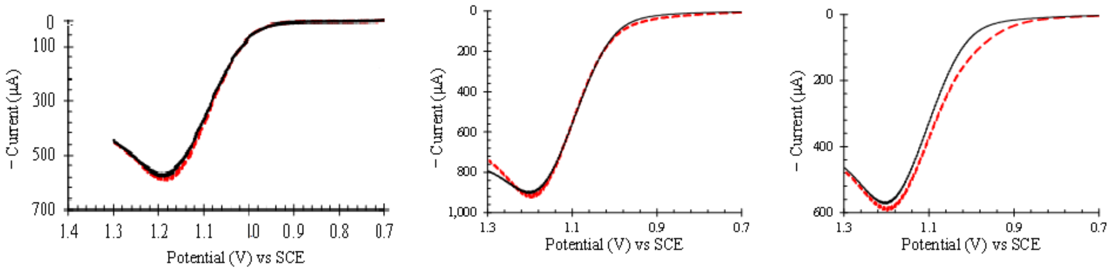

Figure SI.8: LSVs for 0.64 mM Ru(bpy)$_3^{2+}$ in 0.10 M HNO_3 at 200 mV/s are shown for a Pt disk (0.452 cm^2) modified with a Nafion film (left), a magnetized C1 composite (middle) and a demagnetized C1 composite (right) without (black) and with (red dash) an external NdFeB hollow cylinder magnet. For Nafion film and the magnetized composite, application of the strong uniform external field does not alter the voltammogram within the width of line. For the demagnetized composite, the current is increased $\lesssim 5$ %. Voltammogram for the electrodes modified with a Nafion film, the current response is unchanged on introduction of the uniform external field. The uniform magnetic field does not impact electron transfer rate in Nafion films and composites.

presence of the external ring magnet has little to no impact on the observed LSV for the composites, consistent with no impact of the uniform magnetic field on either mass transport or electron transfer. . There is no evidence that magnetohydrodynamics impacts the current response. For the magnetized microparticles, the curves with and without the external ring magnet are nearly superimposed. For the demagnetized composite, the voltammetric peak current with the external magnet is about 5 % greater than without the external uniform field. The small increase may reflect slight magnetization of the demagnetized microparticles once centered in the hollow cylinder magnet. The peak currents for Nafion and demagnetized composites are not statistically different.

Current for Ru(bpy)$_3^{2+}$ is higher for the magnetized composite than the Nafion film. The higher current for the magnetized composite is consistent with the magnetic gradient about the microparticles enhancing the electron self exchange (hopping) rate of the Ru(bpy)$_3^{2|3}$. The effect of the gradient magnetic field is present independent of the uniform field.

Magnetized and demagnetized composites are chemically the same and differ only in the presence and absence of the magnetic gradient. The uniform external field does not effect the voltammetric responses for the Nafion film or the magnetized composite, consistent with the magnetic gradient rather than the magnetic field driving the increase in electron transfer rate. The impact of the magnetic gradient is not altered by the presence of the higher but uniform external field of the NdFeB magnet.

SI.6.3 Quantitative Results for External, Uniform Field Applied with NdFeB Ring Magnet

Results are summarized in Table SI.6 where data are recorded for three replicate electrodes, each recorded at three replicates of four scan rates (20, 50, 100, 200 mV/s). Voltammograms are first recorded with no ring magnet and then for the same electrode with the ring magnet. Solution are N_2 degassed and peak currents for Ru(bpy)$_3^{2+}$ are reported for the reduction on the forward sweep near 1.1 V vs SCE. Results are shown for unmodified Pt disks, Nafion films on Pt disk, and composites of magnetized C1 in Nafion and demagnetized C1 in Nafion. The C1 particles are magnetized and demagnetized before introduction into Nafion suspension and composites are cast and dried on the electrode surface without an external magnet.

In the Table, the second to fourth columns are peak currents with no ring magnet, $\left|i_p^f\right|^{no\_Ring}$. the second column reports the magnitude of the peak current $\left|i_p^f\right|^{no\_Ring}$ at 200 mV/s with standard deviations of the replicates, where the unmodified electrode yields the lowest current, Nafion and demagnetized composite are comparable, and the magnetized composites yield higher current than Nafion and demagnetized composites. The third column is the ratio of the average peak current as compared to Nafion films at 200 mV/s. The fourth column is the ratio to Nafion films across all scan rates. The fifth column reports the impact of the ring magnet as the percent enhancement in peak currents with the magnet $\left|i_p^f\right|^{Ring}$ as compared to no ring magnet at 200 mV/s,

$100\left(\left(|i_p|^{Ring}\,/\,|i_p|^{no\_Ring}\right)-1\right)$. For Nafion and magnetized composites, there is no observed enhancement with the ring magnet. For the demagnetized composite, the enhancement is $\lesssim 5\%$. For the unmodified electrode, the ring magnet increases the peak current for Ru(bpy)$_3^{2+}$ by about 2/3.

A few specific comments relate to the data in the Table where no ring magnet is used. (1) For the magnetized C1 composite, the highest scan rate of 200 mV s^{-1} ensures the diffusion length does not exceed the film and is likely a more reliable estimate of enhancement than the value reported across all scan rates. (2) It is noted that at 200 mV s^{-1}, the peak splitting in Figure 3 bottom is about 170 mV for the Nafion film, consistent with a quasireversible to irreversible charge transfer. The interfacial electron transfer rate is slow compared to the rate of voltage perturbation. There is opportunity for magnetic effects from the uniform field to impact electron transfer rate, but none are observed. (3) The peak current for the unmodified electrode is 23 % of the Nafion film value. The 23% is comparable to the 27 ± 5 % estimated from known concentrations and diffusion coefficients of Ru(bpy)$_3^{2+}$ in Nafion. [55, 80, 81] and the electrolyte.

Results are consistent with negligible enhancements to the peak current of Nafion and composites with the uniform field of the external ring magnet. The field of the iron oxide microparticles is substantially weaker than that of the NdFeB, but the field gradient of the magnetized microparticles is dramatically steeper. Data in Table SI.6 are consistent with the observed rate enhancements driven by field gradients about the microparticles that enhance rates of electron transfer.

	$\left|i_p^f\right|^{no\_Ring}$ $(200\ mV/s)$ No NdFeB Ring at 200 mV/s	% Nafion No NdFeB Ring at 200 mV/s	% Nafion No NdFeB Ring across all scan rates	$100\frac{|i_p|^{Ring}-|i_p|^{no\_Ring}}{|i_p|^{no\_Ring}}$ NdFeB Ring 200 mV/s
Unmodified	130±4	22.8±0.8%	19±3%	+65%
Nafion	570±20	-	-	-
Mag. C1 Composite	890±90	156±17%	195±32%	no
Demag. C1 Composite	600±50	105±10%	104±2%	$\lesssim +5\%$

Table SI.6: Peak Currents and Peak Current Ratios for Ru(bpy)$_3^{2+}$ at 0.452 cm^2 Pt Disks Unmodified and Modified with Nafion, Nafion + Magnetized C1, or Nafion + Demagnetized C1 with and without Uniform Magnetic Field Applied with an External NdFeB Ring Magnet. Columns 2 to 4 desscribe data without the ring magnet. Column 5 shows the impact of the ring magnet. Details are provided in the SI text.

SI.6.4 Gradient and the Electrode Electrolyte Interface

Initial discussion of the relationships between magnetization M, particle radius r_0 and distance from the particle surface Δr is provided in SI.3.1.2.

For a sphere with magnetization M (dipole moment per volume) magnetic sphere of radius r_0 and and a distance Δr from the surface where $r = r_0 + \Delta r$, the magnetic field at r is proportional to $-Mr_0^3r^{-3}$ and the gradient at r is its derivative, $3Mr_0^3r^{-4}$. [96] As the volume of the sphere and so r_0 increase, the field increases but the gradient decreases. As $\Delta r/r_0 \longrightarrow 0$, $3Mr_0^3r^{-4} \longrightarrow 3Mr_0^{-1}$.

At the electrode electrolyte interface, the elementary electron transfer event occurs immediately at the interface where $\Delta r \lesssim 1$ nm. The electrolyte is diamagnetic. The magnitude of the gradient is set by $\Delta r/r_0$ and the length scale of r_0.

Paramagnetic Atom For a paramagnetic metal atom of magnetization M, r_0 and Δr are similar. Consider $r_0 = \Delta r$, then the field is $-M$, but the gradient is very steep because r_0 is of the order of tenth nanometers, $3Mr_0^3r^{-4} = 3Mr_0^3\left(r_0+\Delta r\right)^{-4} \longrightarrow 3Mr_0^3\left(2r_0\right)^{-4} = (3/16)\,Mr_0^{-1}$.

In diamagnetic atoms, $M \lessapprox 0$.

Microparticles In studies of magnetized composite, r_0 is on the order of micrometers, $\Delta r/r_0 \longrightarrow 0$. The magnetization sets the field as $-M$. The gradient is set by the particle radius as $3Mr_0^{-1}$. At microparticles, the

gradient is shallow compared to a paramagnetic atom. The steepness of the gradient is set by M and r_0, which is on the order of micrometers for the microparticles. A balance of M and r_0 sets the gradient where magnetized microparticles are used.

Uniform Field In uniform fields, the gradient is zero and the field is constant. Mathematically, in spherical coordinates, uniform field and zero gradient is achieved where r_0 and r are comparable and large, on the order of centimeters.

Note on Generation of a Gradient Field with Standard Laboratory Magnets The gradients on a micrometer or nanometer length scale are very steep because the distances r_0 and Δr are small. Standard laboratory magnets such as electromagnets and macroscopic rare earth magnets generate strong magnetic fields, on the order of several Tesla. But because r_0 is large and Δr remains small, generation of a sufficiently steep gradient over Δr with a standard laboratory magnet is not anticipated.

References

[1] S. Trasatti, "Work function, electronegativity, and electrochemical behavior of metals. iii. electrolytic hydrogen evolution in acid solution," *Electroanalytical Chemistry and Interfacial Electrochemistry*, vol. 39, pp. 163–184, 1972.

[2] S. Trasatti, "Work function, electronegativity and electrochemical behaviour of metals. i. selection of experimental values of work function," *Chimica e Industria*, vol. 53, pp. 559–564, 1971.

[3] S. Trasatti, "Work function, electronegativity, and electrochemical behavior of metals. ii. potentials of zero charge and electrochemical work functions," *J. Electroanal. Chem.*, vol. 33, pp. 351–378, 1971.

[4] K. L. Knoche, "Density gradient films, lanthanide electrochemistry, and magnetic field effects on hydrogen evolution, oxygen reduction, and lanthanide electrochemistry," Ph.D. dissertation, University of Iowa, 2015, 2015.

[5] H. C. Lee, "Magnetic field effects on photoelectrochemical hydrogen evolution, heterogeneous and self exchange reactions," Ph.D., University of Iowa, 2011, 2011.

[6] O. Devos, O. Aaboubi, J.-P. Chopart, A. Olivier, C. Gabrielli, and B. Tribollet, "Is there a magnetic effect on electrochemical kinetics?" *J. Phys. Chem. A*, vol. 104, pp. 1544–1548, 2000.

[7] S. Koehler and A. Bund, "Investigations on the kinetics of electron transfer reactions in magnetic fields," *Journal of Physical Chemistry B*, vol. 110, pp. 1485–1489, 2006.

[8] Y. Jiao, Y. Zheng, M. Jaroniecb, and S. Z. Qiao, "Design of electrocatalysts for oxygen- and hydrogen-involving energy conversion reactions," *Chem. Soc. Rev.*, vol. 44, pp. 2060–2086, 2015.

[9] A. Bard and L. Faulkner, *Electrochemical Methods*, 2nd ed. New York: John Wiley & Sons, Inc., 2001.

[10] K. S. Dadallagei, D. L. Parr IV, J. R. Coduto, A. Lazicki, S. DeBie, C. D. Haas, and J. Leddy, "New Perspectives from Classical Transition State Theory: The Hydrogen Evolution Reaction (HER) on Metal Electrodes," *J. Electrochem. Soc.*, vol. 107, 086508, 2023.

[11] O. Markovitch and N. Agmon, "Structure and energetics of the hydronium hydration shells," *J. Phys. Chem. A*, vol. 111, pp. 2253–2256, 2007.

[12] W. L. Gellett, "Magnetic microparticles on electrodes: Polymer electrolyte membrane fuel cells, carbon monoxide oxidation, and transition metal complex electrochemistry," Ph.D., University of Iowa, 2004, 2004.

[13] K. A. Mauritz and R. B. Moore, "State of understanding of nafion," *Chem. Rev.*, vol. 104, pp. 4535–4585, 2004.

[14] T. Fahidy, "Magnetoelectrolysis," *J. Appl. Electrochem.*, vol. 13, pp. 553–563, 1983.

[15] T. Z. Fahidy, "The effect of magnetic fields on electrochemical processes," in *Modern Aspects of Electrochemistry*, 1999, vol. 32, pp. 333–354.

[16] M. C. Weston, M. D. Gerner, and I. Fritsch, "Magnetic fields for fluid motion," *Analytical Chemistry*, vol. 82, no. 9, pp. 3411–3418, Apr 2010.

[17] N. Turro and B. Kraeutler, "Magnetic field and magnetic isotope effects in organic photochemical reactions. a novel probe of reaction mechanisms and a method for enrichment of magnetic isotopes," *Accounts of Chemical Research*, vol. 13, pp. 369–377, 1980.

[18] A. Buchachenko, "Magnetic effects in chemical reactions," *Russ. Chem. Rev.*, vol. 45, pp. 375–390, 1976.

[19] R. Sagdeev, K. Salikhov, and Y. M. Molin, "The influence of the magnetic field on processes involving radicals and triplet molecules in solutions," *Russ. Chem. Rev.*, vol. 46, pp. 297–315, 1977.

[20] P. Atkins, "Magnetic field effects," *Chemistry in Britian*, vol. 12, pp. 214–228, 1976.

[21] P. Atkins and T. Lambert, "The effect of a magnetic field on chemical reactions," *Annual Report of Progress in Chemistry*, vol. 72A, pp. 67–88, 1975.

[22] U. Steiner and T. Ulrich, "Magnetic field effects in chemical kinetics and related phenomena," *Chem. Rev.*, vol. 89, pp. 51–147, 1989.

[23] T. Z. Fahidy, "Magnetoelectrolysis," *Journal of Applied Electrochemistry*, vol. 13, no. 5, pp. 553–563, 9 1983, [Online; accessed 2023-07-23].

[24] L. M. Monzon and J. Coey, "Magnetic fields in electrochemistry: The Lorentz force. A mini-review," *Electrochemistry Communications*, vol. 42, pp. 38–41, 5 2014, [Online; accessed 2023-07-23].

[25] J. M. Coey, "Magnetoelectrochemistry," *Europhysics News*, vol. 34, no. 6, pp. 246–248, 11 2003, [Online; accessed 2023-07-23].

[26] V. Gatard, J. Deseure, and M. Chatenet, "Use of magnetic fields in electrochemistry: A selected review," *Current Opinion in Electrochemistry*, vol. 23, pp. 96–105, 10 2020, [Online; accessed 2023-07-23].

[27] I. Willner and E. Katz, "Magnetic Control of Electrocatalytic and Bioelectrocatalytic Processes," *Angewandte Chemie International Edition*, vol. 42, no. 38, pp. 4576–4588, oct 6 2003, [Online; accessed 2023-07-23].

[28] S. Raj K A and C. S. Rout, "Recent developments, challenges and future prospects of magnetic field effects in supercapacitors," *Journal of Materials Chemistry A*, vol. 11, no. 11, pp. 5495–5519, 2023.

[29] C. Biz, J. Gracia, and M. Fianchini, "Review on magnetism in catalysis: From theory to pemfc applications of 3d metal pt-based alloys," *International Journal of Molecular Sciences*, vol. 23, no. 23, p. 14768, Nov 2022.

[30] S. Luo, K. Elouarzaki, and Z. J. Xu, "Electrochemistry in Magnetic Fields," *Angewandte Chemie International Edition*, vol. 61, no. 27, may 25 2022, [Online; accessed 2023-07-23].

[31] A. Kumar, P. Mondal, and C. Fontanesi, "Chiral Magneto-Electrochemistry," *Magnetochemistry*, vol. 4, no. 3, p. 36, aug 18 2018, [Online; accessed 2023-07-23].

[32] Y. Zhang, C. Liang, J. Wu, H. Liu, B. Zhang, Z. Jiang, S. Li, and P. Xu, "Recent advances in magnetic field-enhanced electrocatalysis," *ACS Applied Energy Materials*, vol. 3, no. 11, pp. 10 303–10 316, Nov 2020.

[33] L. Zhang, D. Wu, and X. Yan, "Applications of magnetic field for electrochemical energy storage," *Applied Physics Reviews*, vol. 9, no. 3, p. 031307, Sep 2022.

[34] K. Wang, Q. Yang, H. Zhang, M. Zhang, H. Jiang, C. Zheng, and J. Li, "Recent advances in catalyst design and activity enhancement induced by a magnetic field for electrocatalysis," *Journal of Materials Chemistry A*, vol. 11, no. 15, pp. 7802–7832, 2023, [Online; accessed 2023-07-23].

[35] P. U. Arumugam, E. A. Clark, and I. Fritsch, "Use of paired, bonded ndfeb magnets in redox magnetohydrodynamics," *Anal. Chem.*, vol. 77, pp. 1167–1171, 2005.

[36] Z. P. Aguilar, P. Arumugam, and I. Fritsch, "Study of magnetohydrodynamic driven flow through ltcc channel with self-contained electrodes," *Journal of Electroanalytical Chemistry*, vol. 591, pp. 201–209, 2006.

[37] E. C. Anderson and I. Fritsch, "Factors influencing redox magnetohydrodynamic-induced convection for enhancement of stripping analysis," *Analytical Chemistry*, vol. 78, pp. 3745–3751, 2006.

[38] P. U. Arumugam, E. S. Fakunle, E. C. Anderson, S. R. Evans, K. G. King, Z. P. Aguilar, C. S. Carter, and I. Fritsch, "Characterization and pumping," *Journal of The Electrochemical Society*, vol. 153, pp. E185–E194, 2006.

[39] E. A. Clark and I. Fritsch, "Anodic stripping voltammetry enhancement by redox magnetohydrodynamics," *Anal. Chem.*, vol. 76, pp. 2415–2418, 2004.

[40] S. R. Ragsdale, J. Lee, X. Gao, and H. S. White, “Magnetic field effects in electrochemistry. voltammetric reduction of acetophenone at microdisk electrodes,” *The Journal of Physical Chemistry*, vol. 100, no. 14, pp. 5913–5922, 1996.

[41] S. Ragsdale, K. Grant, and H. White, “Electrochemically generated magnetic forces. enhanced transport of a paramagnetic redox species in large, nonuniform magnetic fields,” *J. Am. Chem. Soc.*, vol. 120, pp. 13 461–13 468, 1998.

[42] J. Lee, X. Gao, L. D. A. Hardy, and H. S. White, “Influence of magnetic fields on the voltammetric response of microelectrodes in highly concentrated organic redox solutions,” *Journal of the Electrochemical Society*, vol. 142, pp. L90–L92, 1995.

[43] N. Leventis and X. Gao, “Nd-fe-b permanent magnet electrodes. theoretical evaluation and experimental demonstration of the paramagnetic body forces,” *J. Am. Chem. Soc.*, vol. 124, pp. 1079–1088, 2002.

[44] N. Leventis and X. Gao, “Magnetohydrodynamic electrochemistry in the field of nd-fe-b magnets. theory, experiment, and application in self-powered flow delivery systems,” *Anal. Chem.*, vol. 73, pp. 3981–3992, 2001.

[45] N. Leventis and A. Dass, “Demonstration of the elusive concentration-gradient paramagnetic force,” *Journal of the American Chemical Society*, vol. 127, pp. 4988–4989, 2005.

[46] S. Legeai, M. Chatelut, O. Vittori, J.-P. Chopart, and O. Aaboubi, “Magnetic field influence on mass transport phenomena,” *Electrochimica Acta*, vol. 50, pp. 51–57, 2004.

[47] K. Tschulik, J. A. Koza, M. Uhlemann, A. Gebert, and L. Schultz, “Effects of well-defined magnetic field gradients on the electrodeposition of copper and bismuth,” *Electrochemistry Communications*, vol. 11, no. 11, pp. 2241–2244, Nov 2009.

[48] J. Linnemann, K. Kanokkanchana, and K. Tschulik, “Design strategies for electrocatalysts from an electrochemist’s perspective,” *ACS Catalysis*, vol. 11, no. 9, pp. 5318–5346, Apr 2021.

[49] K. Ngamchuea, K. Tschulik, and R. G. Compton, “Magnetic control: Switchable ultrahigh magnetic gradients at fe3o4 nanoparticles to enhance solution-phase mass transport,” *Nano Research*, vol. 8, no. 10, pp. 3293–3306, Sep 2015.

[50] P. Cignoni, N. Blanc, and K. Tschulik, “Why standard electrokinetic analysis often fails for nanostructured electrodes – reviewing inhomogeneous electroactivity,” *Current Opinion in Electrochemistry*, vol. 38, p. 101225, Apr 2023.

[51] C. Rurainsky, D.-R. Nettler, T. Pahl, A. Just, P. Cignoni, K. Kanokkanchana, and K. Tschulik, “Electrochemical dealloying in a magnetic field – tapping the potential for catalyst and material design,” *Electrochimica Acta*, vol. 426, p. 140807, Sep 2022.

[52] R. T. Weidner and R. L. Sells, *Elementary Modern Physics*, 2nd ed. Allyn and Bacon, 1973.

[53] O. E. Glukhova and M. M. Slepchenkov, “Electronic properties of the functionalized porous glass-like carbon,” *J. Phys. Chem C*, vol. 120, pp. 17 753–17 758, 2016.

[54] T. Gierke and W. Hsu, “The cluster-network model of ion clustering in perfluorosulfonated membranes,” in *Perfluorinated Ionomer Membranes*, H. Yeager and A. Eisenberg, Eds., vol. 180. Washington, D.C.: American Chemical Society, 1982, pp. 283–307.

[55] J. Leddy, “Modification of nafion membranes: Tailoring properties for function,” in *Nanomaterials for Energy*, ser. ACS Symposium Series, J. Liu, S. Bashir, and L.-D. Chen, Eds. American Chemical Society, 2015, vol. 1213, pp. 99–133.

[56] W. Gellett, D. Dunwoody, and J. Leddy, “Self-hydrating polymer electrolyte fuel cells,” in *41st Power Sources Proceedings*, Philadelphia, PA, 2004, pp. 251–254.

[57] D. Dunwoody, W. Gellett, H. Chung, and J. Leddy, “Magnetic modification of proton exchange membrane fuel cells for improved carbon monoxide tolerance,” in *40th Power Sources Conference*, Cherry Hill, NJ, 2002, pp. 262–265.

[58] J. Leddy and H. Chung, “Magnetically modified fuel cells,” in *39th Power Sources Proceedings*, 2000, pp. 144–147.

[59] G. G. Lee, J. Leddy, and S. D. Minteer, "Enhancing alcohol electrocatalysis with the introduction of magnetic composites to nickel electrocatalysts," *Chem. Comm.*, vol. 48, pp. 11 972–11 974, 2012.

[60] P. Zou and J. Leddy, "Magnetized nickel electrodes for improved charge and discharge rates in nickel metal hydride and nickel cadmium batteries," *Electrochemical and Solid-State Letters*, vol. 9, pp. A43–A45, 2006.

[61] J. P. Tesene and J. Leddy, "Magnetically modified alkaline batteries - improved primaries and attractive secondaries," pp. 161–163, 2006.

[62] G. G. Lee and J. Leddy, "Magnetically modified dye sensitized solar cells," *ECS Transactions*, vol. 41, pp. 83–91, 2011.

[63] J. Leddy, W. L. Gellett, and D. C. Dunwoody, "Self-hydrating membrane electrode assemblies for fuel cells,", US Patent 8,227,134, 2012.

[64] S. Amarasinghe, S. Minteer, L. A. Zook, D. C. Dunwoody, C. Spolar, H. Chung, and J. Leddy, "Magnetically enhanced composite materials and methods for making and using the same," US Patent 6,355,166, 2002.

[65] J. Leddy, W. L. Gellett, and D. C. Dunwoody, "Methods for increasing carbon monoxide tolerance in fuel cells," US Patent Application 2010/0291415, 2010.

[66] J. Leddy and P. Zou, "Magnetically modified electrodes as well as methods of making and using the same," US Patent 6,890,670, 2005.

[67] J. Leddy and P. Zou, "Methods for forming magnetically modified electrodes and articles produced thereby," US Patent 7,709,115, 2010.

[68] J. Leddy and J. Tesene, "Batteries and battery components with magnetically modified manganese dioxide," US Patent 8,231,988, 2012.

[69] J. Leddy and P. N. Motsegood, "Magnetized battery cathodes," US Patent Application 2012/0088148, 2012.

[70] J. Leddy and J. J. Reed, "Magnetically modified metals and metal alloys for hydride storage," US Patent Application 2014/0378016, 2014.

[71] J. Leddy and G. G. Lee, "Magnetically modified manganese dioxide electrodes for asymmetric supercapacitors," US Patent Application 2013/0308248, 2013.

[72] J. Leddy and H. C. Lee, "Magnetically modified semiconductor electrodes for photovoltaics, photoelectrosynthesis, and photocatalysis," US Patent Application 2011/0214997, 2011.

[73] J. O. Bockris, "Electrolytic polarisation—I. The overpotential of hydrogen on some less common metals at high current densities. Influence of current density and time," *Trans. Faraday Soc.*, vol. 43, pp. 417–429, 1947, [Online; accessed 2022-06-04].

[74] R. Parsons, "The rate of electrolytic hydrogen evolution and the heat of adsorption of hydrogen," *Transactions of the Faraday Society*, vol. 54, p. 1053, 1958, [Online; accessed 2022-06-04].

[75] B. E. Conway and J. O. Bockris, "Electrolytic Hydrogen Evolution Kinetics and Its Relation to the Electronic and Adsorptive Properties of the Metal," *The Journal of Chemical Physics*, vol. 26, no. 3, pp. 532–541, 3 1957, [Online; accessed 2022-06-05].

[76] J. K. Nørskov, T. Bligaard, A. Logadottir, J. R. Kitchin, J. G. Chen, S. Pandelov, and U. Stimming, "Trends in the exchange current for hydrogen evolution," *Journal of The Electrochemical Society*, vol. 152, pp. J23–J26, 2005.

[77] J. O. Bockris and A. K. N. Reddy, *Modern Electrochemistry*, 1970, vol. 2.

[78] O. A. Petrii and G. A. Tsirlina, "Electrocatalytic activity prediction for hydrogen electrode reaction: Intuition, art, science," *Electrochimica Acta*, vol. 39, pp. 1739–1747, 1994.

[79] L. A. Zook and J. Leddy, "Density and solubility of nafion: Recast, annealed, and commercial films," *Anal. Chem.*, vol. 68, pp. 3793–3796, 1996.

[80] K. J. Oberbroekling, D. C. Dunwoody, S. D. Minteer, and J. Leddy, "Density of nafion exchanged with transisiton metal complexes and tetramethyl ammonium, ferrous, and hydrogen ions: Commercial and recast films," *Anal. Chem.*, vol. 74, pp. 4794–4799, 2002.

[81] W. L. Gellett, K. L. Knoche, N. P. W. Rathuwadu, and J. Leddy, "Electron hopping of tris (2,2'-bipyridyl) transition metal complexes m(bpy)$_3^{2/3}$ in nafion," *Journal of The Electrochemical Society*, vol. 163, pp. H588–H597, 2016.

[82] C. P. Hunt, B. M. Moskowitz, and S. K. Banerjee, "Magnetic properties of rocks and minerals," 1995. [Online]. Available: https://agupubs.onlinelibrary.wiley.com/doi /10.1029/RF003p0189

[83] K. M. Krishnan, "Biomedical nanomagnetics: A spin through possibilities in imaging, diagnostics, and therapy," *IEEE Transactions on Magnetics*, vol. 46, no. 7, pp. 2523–2558, Jul 2010.

[84] A. Kolhatkar, A. Jamison, D. Litvinov, R. Willson, and T. Lee, "Tuning the magnetic properties of nanoparticles," *International Journal of Molecular Sciences*, vol. 14, no. 8, pp. 15 977–16 009, Jul 2013.

[85] S. D. Minteer, "Magnetic field effects on electron transfer reactions," Ph.D., University of Iowa, 2000, 2000.

[86] B. Chance, D. DeVault, H. Frauenfelder, R. Marcus, J. Schrieffer, and N. Sutin, *Tunneling in Biological Systems*. New York: Academic Press, 1979.

[87] H. Dahms, "Electronic conduction in aqueous solution," *J. Phys. Chem.*, vol. 72, pp. 362–364, 1968.

[88] I. Ruff and V. Friedrich, "Transfer diffusion. i. theoretical," *J. Phys. Chem.*, vol. 75, pp. 3297–3302, 1971.

[89] R. A. Marcus, "The theory of oxidation-reduction reactions involving electron transfer. v. comparison and properties of electrochemical and chemical rate constants," *Journal of Physical Chemistry*, vol. 67, pp. 853–857, 1963.

[90] C. D. Haas, "Experimental electrochemistry: Magnetoelectrocatalysis and electrochemical identification of redox-active transition metal complexes," Ph.D., University of Iowa, 2023.

[91] K. L. Knoche, C. Hettige, P. D. Moberg, S. Amarasinghe, and J. Leddy, "Cyclic voltammetric diagnostics for inert, uniform density films," *Journal of the Electrochemical Society*, vol. 16, pp. H285–H293, 2013.

[92] T. Gierke, G. Munn, and F. Wilson, "The morphology in nafion* perfluorinated membrane products, as determined by wide- and small- angle x-ray studies," *Journal of Polymer Science Polymer Physics Edition*, vol. 19, pp. 1687–1704, 1981.

[93] F. I. Allen, L. R. Comolli, A. Kusoglu, M. A. Modestino, A. M. Minor, and A. Z. Weber, "Morphology of hydrated as-cast nafion revealed through cryo electron tomography," *ACS Macro Letters*, vol. 4, pp. 1–5, 2014.

[94] Z. Ogumi, T. Kuroe, and Z. ichiro Takehara, "Gas permeation in spe method. ii. oxygen and hydrogen permeation through nafion," *J. Electrochem. Soc.*, vol. 132, pp. 2601–2605, 1985.

[95] P. U. Arumugam, E. S. Fakunle, E. C. Anderson, S. R. Evans, K. G. King, Z. P. Aguilar, C. S. Carter, and I. Fritsch, "Characterization and pumping. redox magnetohydrodynamics in a microfluidic channel," *Journal of the Electrochemical Society*, vol. 153, pp. E185–E194, 2006.

[96] R. Fitzpatrick, "Uniformly magnetized sphere," Jun 2014. [Online]. Available: http://farside.ph.utexas.edu/teaching/jk1/ lectures/node61.html
